# Supplementary material for: Drosophila Larval Brain Neoplasms Present Tumour-Type Dependent Genome Instability
Source: G3 (Bethesda). 2018 Feb 26;8(4):1205–14. doi: 10.1534/g3.117.300489 (PMC5873911; doi:10.1534/g3.117.300489)
Supplement: Supplementary file 1 [file 1205FileS1.pdf]

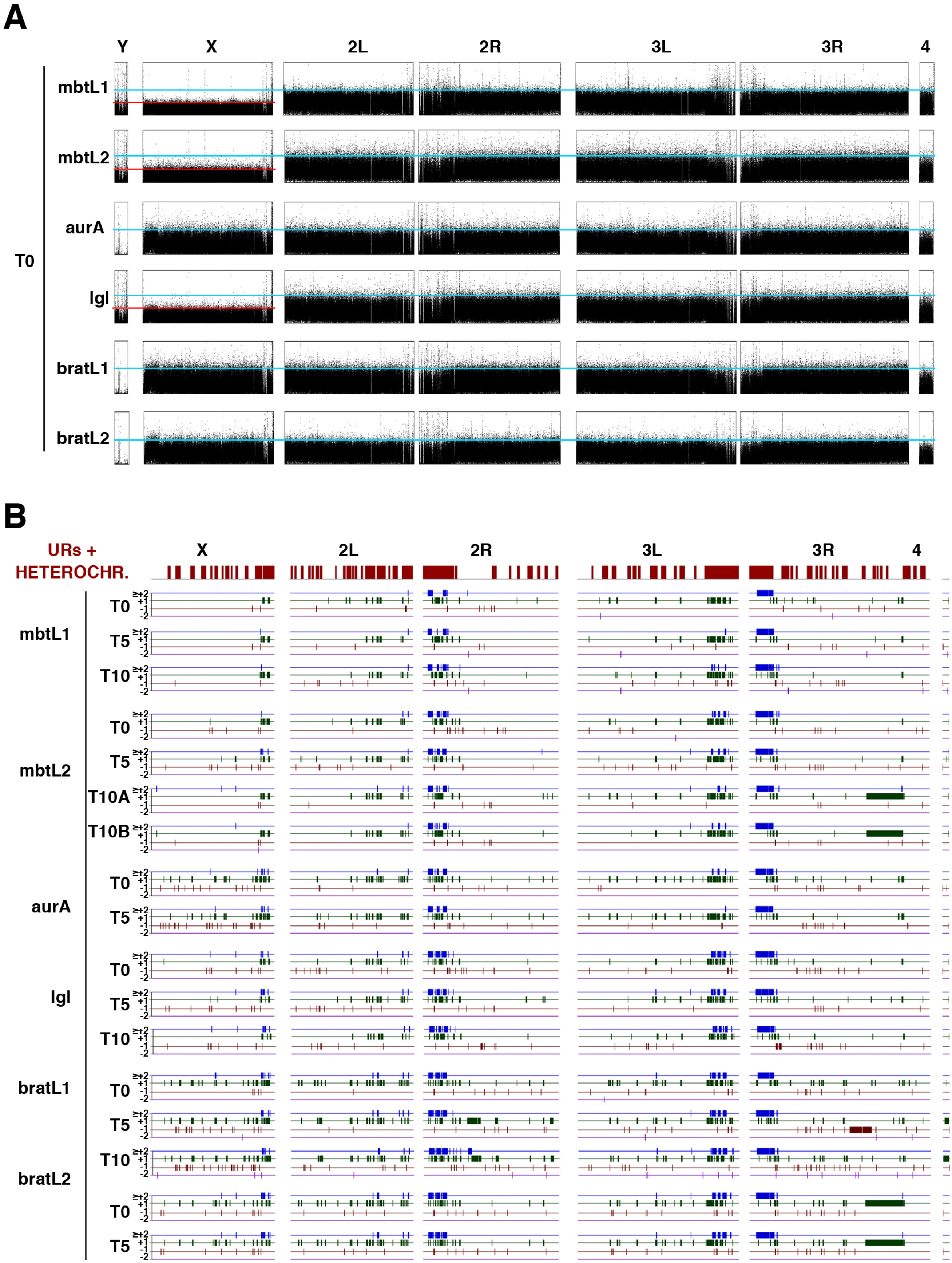
A
Y
X
2L
2R
3L
3R
4
mbtL1
mbtL2
aurA
lgl
bratL1
bratL2
T0
B
URs +
HETEROCHR.
X
2L
2R
3L
3R
4
mbtL1
mbtL2
aurA
lgl
bratL1
bratL2
T0
T5
T10
T10A
T10B
≥+2
+1
-1
-2

Figure S1

Figure S1. Sequence coverage and first draft map of CNVs. A) Overview of sequence coverage over the genome at the first round of allograft (T0). The halved coverage of the X chromosome compared to that of the autosome arms and the significant coverage of Y chromosome specific sequences in mtbtL1, mbtL2, and lgl indicates that these lines originated in male larvae. B) Map of CNVs identified in different lines at different time points. Copy number gains ($\geq$+2 , blue; +1, green) and losses (-1, red; and $\leq$-2, purple) are mapped along chromosome arms X, 2L, 2R, 3L, 3R, and 4th. The heterochromatic Y chromosome is omitted. A very significant fraction of copy number gains map on under-replicated regions (URs and heterochromatin; shown in brown at the top of the map).

sample	chromosome	start	end	CNV size	CNV
aura T0	chr2L	3193001	3251000	58000	1
aura T0	chr2L	4019001	4162000	143000	1
aura T0	chr2L	4513001	4781000	268000	1
aura T0	chr2L	8773001	8890000	117000	1
aura T0	chr2L	18276001	18391000	115000	1
aura T0	chr2R	8272001	8273000	1000	1
aura T0	chr2R	8274001	8384000	110000	1
aura T0	chr2R	8776001	8787000	11000	1
aura T0	chr3L	1040001	1138000	98000	1
aura T0	chr3L	9102001	9116000	14000	1
aura T0	chr3L	22121001	22245000	124000	1
aura T0	chr3L	22275001	22278000	3000	1
aura T0	chr3L	22312001	22316000	4000	1
aura T0	chr3R	7321001	7413000	92000	1
aura T0	chr3R	12512001	12566000	54000	1
aura T0	chr3R	17850001	17982000	132000	1
aura T0	chr3R	26293001	26387000	94000	1
aura T0	chr3R	27474001	27521000	47000	1
aura T0	chr3R	27709001	27906000	197000	1
aura T0	chr3R	28269001	28289000	20000	1
aura T0	chr3R	28429001	28523000	94000	1
aura T0	chrX	1203001	1221000	18000	-1
aura T0	chrX	7172001	7237000	65000	1
aura T0	chrX	8326001	8331000	5000	1
aura T0	chrX	8344001	8353000	9000	1
aura T0	chrX	8749001	8838000	89000	1
aura T0	chrX	11108001	11119000	11000	-1
aura T0	chrX	12391001	12430000	39000	1
aura T0	chrX	12948001	13032000	84000	1
aura T0	chrX	15147001	15239000	92000	1
aura T0	chrX	16557001	16568000	11000	-1
aura T0	chrX	20392001	20411000	19000	-1
aura T0	chrX	21445001	21458000	13000	1
aura T0	chrX	22584001	22629000	45000	1
aura T5	chr2L	4018001	4173000	155000	1
aura T5	chr2L	5014001	5018000	4000	-1
aura T5	chr2L	5018001	5030000	12000	1
aura T5	chr2L	5030001	5036000	6000	-1
aura T5	chr2L	7614001	7618000	4000	1
aura T5	chr2L	8779001	8876000	97000	1
aura T5	chr2L	12617001	12659000	42000	1
aura T5	chr2L	15150001	15718000	568000	1
aura T5	chr2R	6501001	6599000	98000	1
aura T5	chr2R	7259001	7280000	21000	-1
aura T5	chr2R	8274001	8384000	110000	1
aura T5	chr2R	15076001	15085000	9000	1
aura T5	chr2R	15963001	15979000	16000	-1
aura T5	chr2R	17796001	17846000	50000	1
aura T5	chr3L	97001	142000	45000	1
aura T5	chr3L	607001	648000	41000	1
aura T5	chr3L	1041001	1167000	126000	1
aura T5	chr3L	9102001	9109000	7000	1

aura T5	chr3L	12744001	12793000	49000	-1
aura T5	chr3L	12996001	13242000	246000	-1
aura T5	chr3L	19994001	20028000	34000	-1
aura T5	chr3L	22080001	22250000	170000	1
aura T5	chr3R	4476001	4481000	5000	1
aura T5	chr3R	9158001	9181000	23000	-1
aura T5	chr3R	11372001	11377000	5000	-1
aura T5	chr3R	12512001	12602000	90000	1
aura T5	chr3R	13410001	13423000	13000	-1
aura T5	chr3R	27474001	27521000	47000	1
aura T5	chr3R	28269001	28289000	20000	1
aura T5	chr3R	28431001	28523000	92000	1
aura T5	chr4	1189001	1227000	38000	-1
aura T5	chrX	1434001	1480000	46000	-1
aura T5	chrX	1998001	2003000	5000	-1
aura T5	chrX	2688001	2692000	4000	-1
aura T5	chrX	4618001	4628000	10000	-1
aura T5	chrX	4681001	4686000	5000	-1
aura T5	chrX	4724001	4906000	182000	1
aura T5	chrX	5531001	5569000	38000	1
aura T5	chrX	6436001	6455000	19000	1
aura T5	chrX	8747001	8838000	91000	1
aura T5	chrX	10941001	10948000	7000	-1
aura T5	chrX	11073001	11077000	4000	-1
aura T5	chrX	11110001	11123000	13000	-1
aura T5	chrX	11587001	11598000	11000	-1
aura T5	chrX	12390001	12430000	40000	1
aura T5	chrX	15148001	15269000	121000	1
aura T5	chrX	16011001	16048000	37000	-1
aura T5	chrX	16558001	16568000	10000	-1
aura T5	chrX	19778001	19782000	4000	-1
aura T5	chrX	19852001	19872000	20000	-1
aura T5	chrX	20392001	20411000	19000	-1
aura T5	chrX	21352001	21357000	5000	-1
aura T5	chrX	21423001	21458000	35000	1
aura T5	chrX	22548001	22629000	81000	1
bratL1 T0	chr2L	1250001	1271000	21000	1
bratL1 T0	chr2L	2962001	2986000	24000	-1
bratL1 T0	chr2L	4016001	4140000	124000	1
bratL1 T0	chr2L	7726001	7744000	18000	-1
bratL1 T0	chr2L	8745001	8888000	143000	1
bratL1 T0	chr2L	13899001	14157000	258000	1
bratL1 T0	chr2L	18275001	18421000	146000	1
bratL1 T0	chr2R	6501001	6586000	85000	1
bratL1 T0	chr2R	8270001	8366000	96000	1
bratL1 T0	chr2R	18332001	18382000	50000	1
bratL1 T0	chr2R	22718001	22723000	5000	-1
bratL1 T0	chr2R	23359001	23383000	24000	-1
bratL1 T0	chr3L	1075001	1137000	62000	1
bratL1 T0	chr3L	1290001	1293000	3000	-1
bratL1 T0	chr3L	1758001	1791000	33000	-1
bratL1 T0	chr3L	10495001	10555000	60000	1
bratL1 T0	chr3L	10742001	10835000	93000	1

bratL1 T0	chr3L	14253001	14482000	229000	1
bratL1 T0	chr3L	22106001	22216000	110000	1
bratL1 T0	chr3R	7275001	7428000	153000	1
bratL1 T0	chr3R	12166001	12186000	20000	1
bratL1 T0	chr3R	12515001	12586000	71000	1
bratL1 T0	chr3R	26273001	26396000	123000	1
bratL1 T0	chr3R	27324001	27517000	193000	1
bratL1 T0	chr3R	28429001	28484000	55000	1
bratL1 T0	chrX	1588001	1606000	18000	1
bratL1 T0	chrX	4447001	4519000	72000	1
bratL1 T0	chrX	4721001	4911000	190000	1
bratL1 T0	chrX	5082001	5283000	201000	1
bratL1 T0	chrX	6374001	6462000	88000	1
bratL1 T0	chrX	7142001	7278000	136000	1
bratL1 T0	chrX	7298001	7631000	333000	1
bratL1 T0	chrX	8285001	8378000	93000	1
bratL1 T0	chrX	8730001	8835000	105000	1
bratL1 T0	chrX	9376001	9395000	19000	1
bratL1 T0	chrX	10947001	11043000	96000	1
bratL1 T0	chrX	12390001	12421000	31000	1
bratL1 T0	chrX	12678001	12680000	2000	-1
bratL1 T0	chrX	12688001	12693000	5000	-1
bratL1 T0	chrX	12977001	13029000	52000	1
bratL1 T0	chrX	14194001	14242000	48000	-1
bratL1 T0	chrX	14632001	14719000	87000	1
bratL1 T0	chrX	14720001	14739000	19000	1
bratL1 T0	chrX	15146001	15257000	111000	1
bratL1 T0	chrX	15258001	15279000	21000	1
bratL1 T0	chrX	18915001	19112000	197000	1
bratL1 T0	chrX	22548001	22629000	81000	1
bratL1 T10	chr2L	1242001	1271000	29000	1
bratL1 T10	chr2L	3901001	4145000	244000	1
bratL1 T10	chr2L	4145001	4161000	16000	1
bratL1 T10	chr2L	4962001	5544000	582000	1
bratL1 T10	chr2L	6976001	6985000	9000	-1
bratL1 T10	chr2L	8760001	8890000	130000	1
bratL1 T10	chr2L	11991001	12002000	11000	-1
bratL1 T10	chr2L	18275001	18392000	117000	1
bratL1 T10	chr2R	7894001	7919000	25000	1
bratL1 T10	chr2R	7919001	8366000	447000	2
bratL1 T10	chr2R	8368001	8609000	241000	2
bratL1 T10	chr2R	8609001	9863000	1254000	1
bratL1 T10	chr2R	10019001	10526000	507000	1
bratL1 T10	chr2R	11333001	11356000	23000	-1
bratL1 T10	chr2R	13482001	13991000	509000	1
bratL1 T10	chr2R	15963001	15975000	12000	-1
bratL1 T10	chr2R	18330001	18382000	52000	1
bratL1 T10	chr2R	18383001	18415000	32000	1
bratL1 T10	chr2R	23355001	23392000	37000	-1
bratL1 T10	chr2R	23703001	23710000	7000	-1
bratL1 T10	chr2R	24564001	25254000	690000	1
bratL1 T10	chr3L	1065001	1137000	72000	1
bratL1 T10	chr3L	1951001	1961000	10000	-1

bratL1 T10	chr3L	8473001	8480000	7000	-1
bratL1 T10	chr3L	8584001	8607000	23000	-1
bratL1 T10	chr3L	10495001	10674000	179000	1
bratL1 T10	chr3L	10692001	10858000	166000	1
bratL1 T10	chr3L	11611001	11621000	10000	-1
bratL1 T10	chr3L	11961001	11973000	12000	-1
bratL1 T10	chr3L	16592001	16612000	20000	-1
bratL1 T10	chr3L	22093001	22218000	125000	1
bratL1 T10	chr3R	7306001	7410000	104000	1
bratL1 T10	chr3R	8014001	8024000	10000	-1
bratL1 T10	chr3R	9466001	9473000	7000	-1
bratL1 T10	chr3R	9684001	9700000	16000	-1
bratL1 T10	chr3R	12466001	12512000	46000	-1
bratL1 T10	chr3R	12512001	12601000	89000	1
bratL1 T10	chr3R	13003001	13007000	4000	-1
bratL1 T10	chr3R	17837001	17978000	141000	1
bratL1 T10	chr3R	18948001	18952000	4000	-1
bratL1 T10	chr3R	27324001	27519000	195000	1
bratL1 T10	chr3R	28433001	28487000	54000	1
bratL1 T10	chr3R	29922001	29925000	3000	-1
bratL1 T10	chr4	2001	4000	2000	2
bratL1 T10	chr4	21001	174000	153000	1
bratL1 T10	chr4	181001	1035000	854000	1
bratL1 T10	chr4	1051001	1266000	215000	1
bratL1 T10	chrX	1453001	1480000	27000	-1
bratL1 T10	chrX	1542001	1564000	22000	-1
bratL1 T10	chrX	4617001	4626000	9000	-1
bratL1 T10	chrX	4627001	4634000	7000	-1
bratL1 T10	chrX	4715001	4912000	197000	1
bratL1 T10	chrX	4912001	4916000	4000	1
bratL1 T10	chrX	5090001	5276000	186000	1
bratL1 T10	chrX	6242001	6252000	10000	-1
bratL1 T10	chrX	6269001	6301000	32000	-1
bratL1 T10	chrX	6815001	6833000	18000	-1
bratL1 T10	chrX	7113001	7272000	159000	1
bratL1 T10	chrX	7310001	7643000	333000	1
bratL1 T10	chrX	8286001	8386000	100000	1
bratL1 T10	chrX	11072001	11078000	6000	-1
bratL1 T10	chrX	11110001	11124000	14000	-1
bratL1 T10	chrX	12390001	12421000	31000	1
bratL1 T10	chrX	12688001	12693000	5000	-1
bratL1 T10	chrX	12920001	13032000	112000	1
bratL1 T10	chrX	14194001	14203000	9000	-1
bratL1 T10	chrX	14218001	14242000	24000	-1
bratL1 T10	chrX	14627001	14719000	92000	1
bratL1 T10	chrX	15146001	15257000	111000	1
bratL1 T10	chrX	15329001	15350000	21000	-1
bratL1 T10	chrX	15456001	15469000	13000	-1
bratL1 T10	chrX	15716001	15730000	14000	-1
bratL1 T10	chrX	16041001	16048000	7000	-1
bratL1 T10	chrX	16557001	16574000	17000	-1
bratL1 T10	chrX	18920001	19119000	199000	1
bratL1 T10	chrX	19852001	19872000	20000	-1

bratL1 T10	chrX	20163001	20252000	89000	-1
bratL1 T10	chrX	20292001	20311000	19000	-1
bratL1 T10	chrX	20392001	20411000	19000	-1
bratL1 T10	chrX	22548001	22629000	81000	1
bratL1 T5	chr2L	1250001	1271000	21000	1
bratL1 T5	chr2L	3620001	3635000	15000	-1
bratL1 T5	chr2L	3898001	4145000	247000	1
bratL1 T5	chr2L	4523001	4565000	42000	1
bratL1 T5	chr2L	4964001	5543000	579000	1
bratL1 T5	chr2L	6976001	6986000	10000	-1
bratL1 T5	chr2L	8745001	8891000	146000	1
bratL1 T5	chr2L	18275001	18405000	130000	1
bratL1 T5	chr2R	6501001	6521000	20000	1
bratL1 T5	chr2R	7259001	7277000	18000	-1
bratL1 T5	chr2R	7894001	8267000	373000	1
bratL1 T5	chr2R	8267001	8365000	98000	2
bratL1 T5	chr2R	8365001	8366000	1000	1
bratL1 T5	chr2R	8368001	9863000	1495000	1
bratL1 T5	chr2R	10019001	10281000	262000	1
bratL1 T5	chr2R	10285001	10526000	241000	1
bratL1 T5	chr2R	13482001	13875000	393000	1
bratL1 T5	chr2R	13885001	13992000	107000	1
bratL1 T5	chr2R	18332001	18382000	50000	1
bratL1 T5	chr2R	18383001	18416000	33000	1
bratL1 T5	chr2R	23359001	23392000	33000	-1
bratL1 T5	chr2R	23704001	23710000	6000	-1
bratL1 T5	chr2R	24564001	25254000	690000	1
bratL1 T5	chr3L	989001	993000	4000	-1
bratL1 T5	chr3L	8591001	8600000	9000	-1
bratL1 T5	chr3L	10495001	10673000	178000	1
bratL1 T5	chr3L	10689001	10835000	146000	1
bratL1 T5	chr3L	11613001	11621000	8000	-1
bratL1 T5	chr3L	22096001	22218000	122000	1
bratL1 T5	chr3R	7300001	7410000	110000	1
bratL1 T5	chr3R	12512001	12601000	89000	1
bratL1 T5	chr3R	12601001	12602000	1000	1
bratL1 T5	chr3R	17354001	17851000	497000	-1
bratL1 T5	chr3R	17979001	22092000	4113000	-1
bratL1 T5	chr3R	26365001	26396000	31000	1
bratL1 T5	chr3R	27324001	27521000	197000	1
bratL1 T5	chr3R	28429001	28487000	58000	1
bratL1 T5	chr4	173001	174000	1000	1
bratL1 T5	chr4	181001	1035000	854000	1
bratL1 T5	chr4	1051001	1266000	215000	1
bratL1 T5	chrX	1322001	1542000	220000	-1
bratL1 T5	chrX	1542001	1552000	10000	-1
bratL1 T5	chrX	4617001	4626000	9000	-1
bratL1 T5	chrX	4715001	4912000	197000	1
bratL1 T5	chrX	5090001	5276000	186000	1
bratL1 T5	chrX	6085001	6091000	6000	-1
bratL1 T5	chrX	6106001	6115000	9000	-1
bratL1 T5	chrX	6244001	6252000	8000	-1
bratL1 T5	chrX	6285001	6301000	16000	-1

bratL1 T5	chrX	6815001	6847000	32000	-1
bratL1 T5	chrX	7115001	7278000	163000	1
bratL1 T5	chrX	7281001	7282000	1000	1
bratL1 T5	chrX	7298001	7644000	346000	1
bratL1 T5	chrX	8079001	8094000	15000	1
bratL1 T5	chrX	8270001	8378000	108000	1
bratL1 T5	chrX	8731001	8836000	105000	1
bratL1 T5	chrX	10948001	11047000	99000	1
bratL1 T5	chrX	11110001	11124000	14000	-1
bratL1 T5	chrX	12390001	12421000	31000	1
bratL1 T5	chrX	14194001	14203000	9000	-1
bratL1 T5	chrX	14223001	14242000	19000	-1
bratL1 T5	chrX	15146001	15252000	106000	1
bratL1 T5	chrX	15717001	15730000	13000	-1
bratL1 T5	chrX	17747001	17751000	4000	-1
bratL1 T5	chrX	18909001	19120000	211000	1
bratL1 T5	chrX	20392001	20405000	13000	-1
bratL1 T5	chrX	20439001	20443000	4000	-1
bratL1 T5	chrX	21168001	21230000	62000	1
bratL1 T5	chrX	22548001	22629000	81000	1
bratL2 T0	chr2L	628001	764000	136000	1
bratL2 T0	chr2L	1245001	1501000	256000	1
bratL2 T0	chr2L	4015001	4137000	122000	1
bratL2 T0	chr2L	4489001	4565000	76000	1
bratL2 T0	chr2L	4780001	4785000	5000	1
bratL2 T0	chr2L	8763001	8869000	106000	1
bratL2 T0	chr2L	12471001	12545000	74000	-1
bratL2 T0	chr2L	16782001	16787000	5000	-1
bratL2 T0	chr2L	16787001	16802000	15000	1
bratL2 T0	chr2L	18275001	18362000	87000	1
bratL2 T0	chr2L	18340001	18344000	4000	1
bratL2 T0	chr2R	8297001	8383000	86000	1
bratL2 T0	chr3L	2919001	2976000	57000	1
bratL2 T0	chr3L	7993001	8045000	52000	-1
bratL2 T0	chr3R	4496001	4553000	57000	1
bratL2 T0	chr3R	7267001	7273000	6000	1
bratL2 T0	chr3R	7311001	7396000	85000	1
bratL2 T0	chr3R	9290001	9322000	32000	-1
bratL2 T0	chr3R	12512001	12568000	56000	1
bratL2 T0	chr3R	17849001	17969000	120000	1
bratL2 T0	chr3R	26286001	26372000	86000	1
bratL2 T0	chr3R	27476001	27497000	21000	1
bratL2 T0	chr3R	27503001	27510000	7000	1
bratL2 T0	chr3R	28269001	28295000	26000	1
bratL2 T0	chr3R	28429001	28517000	88000	1
bratL2 T0	chrX	1528001	1552000	24000	-1
bratL2 T0	chrX	5105001	5137000	32000	1
bratL2 T0	chrX	5138001	5247000	109000	1
bratL2 T0	chrX	7139001	7268000	129000	1
bratL2 T0	chrX	7591001	7632000	41000	1
bratL2 T0	chrX	7671001	7698000	27000	-1
bratL2 T0	chrX	10629001	10694000	65000	1
bratL2 T0	chrX	11110001	11124000	14000	-1

bratL2 T0	chrX	11868001	11893000	25000	1
bratL2 T0	chrX	12390001	12419000	29000	1
bratL2 T0	chrX	12420001	12429000	9000	1
bratL2 T0	chrX	15225001	15250000	25000	1
bratL2 T0	chrX	15769001	15785000	16000	1
bratL2 T0	chrX	18951001	19117000	166000	1
bratL2 T0	chrX	19852001	19872000	20000	-1
bratL2 T0	chrX	22587001	22629000	42000	1
bratL2 T5	chr2L	628001	763000	135000	1
bratL2 T5	chr2L	1245001	1501000	256000	1
bratL2 T5	chr2L	4489001	4565000	76000	1
bratL2 T5	chr2L	8764001	8876000	112000	1
bratL2 T5	chr2L	9423001	9428000	5000	1
bratL2 T5	chr2L	10167001	10176000	9000	-1
bratL2 T5	chr2L	12411001	12425000	14000	-1
bratL2 T5	chr2L	15750001	16249000	499000	1
bratL2 T5	chr2L	16721001	16782000	61000	1
bratL2 T5	chr2L	16782001	16787000	5000	-1
bratL2 T5	chr2L	16787001	16802000	15000	1
bratL2 T5	chr2L	18276001	18330000	54000	1
bratL2 T5	chr2L	18340001	18344000	4000	1
bratL2 T5	chr2R	8291001	8385000	94000	1
bratL2 T5	chr2R	9975001	9994000	19000	-1
bratL2 T5	chr3L	14425001	14440000	15000	-1
bratL2 T5	chr3L	14712001	14720000	8000	-1
bratL2 T5	chr3R	4381001	4420000	39000	1
bratL2 T5	chr3R	4444001	4449000	5000	1
bratL2 T5	chr3R	4496001	4559000	63000	1
bratL2 T5	chr3R	7308001	7396000	88000	1
bratL2 T5	chr3R	12512001	12568000	56000	1
bratL2 T5	chr3R	13003001	13007000	4000	-1
bratL2 T5	chr3R	17849001	17969000	120000	1
bratL2 T5	chr3R	20994001	27965000	6971000	1
bratL2 T5	chr3R	27476001	27497000	21000	1
bratL2 T5	chr3R	27503001	27510000	7000	1
bratL2 T5	chr3R	28269001	28284000	15000	1
bratL2 T5	chr3R	28429001	28522000	93000	1
bratL2 T5	chrX	929001	937000	8000	-1
bratL2 T5	chrX	5138001	5267000	129000	1
bratL2 T5	chrX	7139001	7268000	129000	1
bratL2 T5	chrX	7592001	7632000	40000	1
bratL2 T5	chrX	7633001	7635000	2000	1
bratL2 T5	chrX	7669001	7716000	47000	-1
bratL2 T5	chrX	11110001	11124000	14000	-1
bratL2 T5	chrX	11650001	11670000	20000	-1
bratL2 T5	chrX	11685001	11705000	20000	1
bratL2 T5	chrX	12390001	12419000	29000	1
bratL2 T5	chrX	12951001	13016000	65000	1
bratL2 T5	chrX	15225001	15267000	42000	1
bratL2 T5	chrX	18938001	19130000	192000	1
bratL2 T5	chrX	22549001	22586000	37000	1
bratL2 T5	chrX	22587001	22629000	42000	1
lgl T0	chr2L	4406001	4409000	3000	-2

lgl T0	chr2L	4467001	4782000	315000	1
lgl T0	chr2L	5014001	5018000	4000	-2
lgl T0	chr2L	5031001	5036000	5000	-2
lgl T0	chr2L	18275001	18326000	51000	1
lgl T0	chr2L	18381001	18392000	11000	1
lgl T0	chr2R	6517001	6526000	9000	1
lgl T0	chr2R	20821001	20836000	15000	-1
lgl T0	chr3L	8463001	8468000	5000	-2
lgl T0	chr3L	8821001	8828000	7000	1
lgl T0	chr3L	17639001	17688000	49000	-1
lgl T0	chr3L	22122001	22253000	131000	1
lgl T0	chr3R	7318001	7371000	53000	1
lgl T0	chr3R	11372001	11377000	5000	-2
lgl T0	chr3R	13221001	13227000	6000	-1
lgl T0	chr3R	17851001	17985000	134000	1
lgl T0	chr3R	28277001	28287000	10000	1
lgl T0	chr3R	28429001	28523000	94000	1
lgl T0	chrX	8078001	8083000	5000	1
lgl T0	chrX	10466001	10471000	5000	-2
lgl T0	chrX	10941001	10948000	7000	-1
lgl T0	chrX	11109001	11124000	15000	-1
lgl T0	chrX	19778001	19782000	4000	-2
lgl T0	chrX	20174001	20238000	64000	1
lgl T0	chrX	20395001	20411000	16000	-1
lgl T10	chr2L	1951001	1953000	2000	-2
lgl T10	chr2L	4406001	4409000	3000	-2
lgl T10	chr2L	4468001	4781000	313000	1
lgl T10	chr2L	5014001	5018000	4000	-2
lgl T10	chr2L	5031001	5036000	5000	-2
lgl T10	chr2L	12462001	12505000	43000	-1
lgl T10	chr2L	16546001	16600000	54000	-1
lgl T10	chr2R	10357001	10677000	320000	-2
lgl T10	chr2R	18276001	18288000	12000	-1
lgl T10	chr2R	19766001	19810000	44000	1
lgl T10	chr2R	20821001	20831000	10000	-1
lgl T10	chr3L	5696001	5699000	3000	-2
lgl T10	chr3L	8463001	8468000	5000	-2
lgl T10	chr3L	8821001	8828000	7000	1
lgl T10	chr3L	22078001	22253000	175000	1
lgl T10	chr3R	4175001	4606000	431000	-2
lgl T10	chr3R	11372001	11377000	5000	-2
lgl T10	chr3R	13003001	13007000	4000	-2
lgl T10	chr3R	17849001	17985000	136000	1
lgl T10	chr3R	25913001	25917000	4000	1
lgl T10	chr3R	27582001	27589000	7000	-1
lgl T10	chr3R	30825001	30835000	10000	-1
lgl T10	chr4	801001	809000	8000	1
lgl T10	chrX	924001	937000	13000	-1
lgl T10	chrX	1673001	1676000	3000	1
lgl T10	chrX	1998001	2003000	5000	-1
lgl T10	chrX	5217001	5236000	19000	1
lgl T10	chrX	10466001	10471000	5000	-2
lgl T10	chrX	10941001	10955000	14000	-1

lgl T10	chrX	11109001	11124000	15000	-1
lgl T10	chrX	12688001	12693000	5000	-2
lgl T10	chrX	16557001	16568000	11000	-2
lgl T10	chrX	20395001	20411000	16000	-1
lgl T5	chr2L	2947001	2955000	8000	-1
lgl T5	chr2L	4321001	4362000	41000	1
lgl T5	chr2L	4406001	4409000	3000	-2
lgl T5	chr2L	4471001	4781000	310000	1
lgl T5	chr2L	5014001	5018000	4000	-2
lgl T5	chr2L	5030001	5036000	6000	-2
lgl T5	chr2L	5109001	5111000	2000	-1
lgl T5	chr2L	5164001	5185000	21000	-1
lgl T5	chr2L	5272001	5308000	36000	1
lgl T5	chr2L	6140001	6143000	3000	-2
lgl T5	chr2L	8743001	8886000	143000	1
lgl T5	chr2L	16546001	16600000	54000	-1
lgl T5	chr2L	19752001	19768000	16000	1
lgl T5	chr2R	6501001	6612000	111000	1
lgl T5	chr2R	7981001	8116000	135000	1
lgl T5	chr2R	13181001	13183000	2000	-2
lgl T5	chr2R	16815001	16854000	39000	1
lgl T5	chr2R	19725001	19812000	87000	1
lgl T5	chr2R	23567001	23605000	38000	1
lgl T5	chr3L	598001	631000	33000	1
lgl T5	chr3L	3713001	3789000	76000	-1
lgl T5	chr3L	5696001	5699000	3000	-2
lgl T5	chr3L	6553001	6582000	29000	-1
lgl T5	chr3L	8452001	8463000	11000	1
lgl T5	chr3L	8463001	8468000	5000	-2
lgl T5	chr3L	8801001	8828000	27000	1
lgl T5	chr3L	17658001	17721000	63000	-1
lgl T5	chr3L	22078001	22253000	175000	1
lgl T5	chr3R	4949001	5148000	199000	1
lgl T5	chr3R	7313001	7372000	59000	1
lgl T5	chr3R	11241001	11372000	131000	1
lgl T5	chr3R	11372001	11377000	5000	-1
lgl T5	chr3R	11770001	11793000	23000	1
lgl T5	chr3R	13003001	13007000	4000	-2
lgl T5	chr3R	15511001	15532000	21000	1
lgl T5	chr3R	16210001	16257000	47000	1
lgl T5	chr3R	17048001	17072000	24000	1
lgl T5	chr3R	17479001	17506000	27000	1
lgl T5	chr3R	17694001	17714000	20000	1
lgl T5	chr3R	19412001	19587000	175000	-1
lgl T5	chr3R	20736001	20843000	107000	1
lgl T5	chr3R	25157001	25190000	33000	1
lgl T5	chr3R	25990001	26044000	54000	1
lgl T5	chr4	64001	258000	194000	-1
lgl T5	chrX	929001	937000	8000	-1
lgl T5	chrX	1673001	1676000	3000	1
lgl T5	chrX	1998001	2003000	5000	-2
lgl T5	chrX	2688001	2692000	4000	-2
lgl T5	chrX	4548001	4614000	66000	1

lgl T5	chrX	6853001	6865000	12000	1
lgl T5	chrX	7237001	7240000	3000	-2
lgl T5	chrX	7244001	7643000	399000	1
lgl T5	chrX	10941001	10948000	7000	-2
lgl T5	chrX	11110001	11123000	13000	-2
lgl T5	chrX	11139001	11149000	10000	1
lgl T5	chrX	11685001	11756000	71000	1
lgl T5	chrX	12715001	12762000	47000	1
lgl T5	chrX	16544001	16574000	30000	-1
lgl T5	chrX	19507001	19517000	10000	-1
lgl T5	chrX	19778001	19782000	4000	-2
lgl T5	chrX	19782001	19827000	45000	1
lgl T5	chrX	20395001	20403000	8000	-1
lgl T5	chrX	20403001	20411000	8000	-2
mbtL1 T0	chr2L	5001	170000	165000	-1
mbtL1 T0	chr2L	341001	382000	41000	-1
mbtL1 T0	chr2L	584001	803000	219000	1
mbtL1 T0	chr2L	7976001	8127000	151000	-1
mbtL1 T0	chr2L	8934001	8997000	63000	-1
mbtL1 T0	chr2L	16697001	16800000	103000	-1
mbtL1 T0	chr2L	21050001	21136000	86000	-1
mbtL1 T0	chr2L	21245001	21769000	524000	-1
mbtL1 T0	chr2R	21994001	22006000	12000	1
mbtL1 T0	chr2R	24252001	24265000	13000	-1
mbtL1 T0	chr3L	3099001	3110000	11000	1
mbtL1 T0	chr3L	3232001	3239000	7000	-2
mbtL1 T0	chr3L	21899001	21969000	70000	-1
mbtL1 T0	chr3L	22279001	22306000	27000	-1
mbtL1 T0	chr3L	22961001	22963000	2000	1
mbtL1 T0	chr3R	7266001	7274000	8000	-1
mbtL1 T0	chr3R	9461001	9472000	11000	-2
mbtL1 T0	chr3R	9908001	9942000	34000	1
mbtL1 T0	chr3R	21736001	21795000	59000	-1
mbtL1 T0	chr3R	22817001	22830000	13000	1
mbtL1 T0	chr3R	22929001	22945000	16000	1
mbtL1 T0	chr3R	24675001	24708000	33000	-2
mbtL1 T0	chr3R	28339001	28499000	160000	1
mbtL1 T0	chr4	2001	1266000	1264000	-1
mbtL1 T0	chr4	1008001	1060000	52000	-1
mbtL1 T0	chrX	18616001	18652000	36000	1
mbtL1 T10	chr2L	9474001	9486000	12000	-1
mbtL1 T10	chr2R	8228001	8254000	26000	-2
mbtL1 T10	chr2R	23352001	23368000	16000	-1
mbtL1 T10	chr3L	7072001	7099000	27000	-2
mbtL1 T10	chr3L	9104001	9107000	3000	1
mbtL1 T10	chr3L	20757001	20762000	5000	-2
mbtL1 T10	chr3R	14814001	14821000	7000	-1
mbtL1 T10	chr3R	28339001	28515000	176000	1
mbtL1 T10	chr3R	32065001	32069000	4000	-2
mbtL1 T10	chr4	1008001	1060000	52000	-2
mbtL1 T10	chrX	8399001	8406000	7000	-1
mbtL1 T10	chrX	11110001	11127000	17000	-1
mbtL1 T5	chr2L	6985001	7010000	25000	1

mbtL1 T5	chr2L	12470001	12497000	27000	-1
mbtL1 T5	chr2R	8228001	8254000	26000	-2
mbtL1 T5	chr3L	1086001	1096000	10000	1
mbtL1 T5	chr3L	6635001	6650000	15000	1
mbtL1 T5	chr3L	7074001	7099000	25000	-2
mbtL1 T5	chr3L	9102001	9103000	1000	1
mbtL1 T5	chr3L	9104001	9106000	2000	1
mbtL1 T5	chr3R	22829001	22830000	1000	1
mbtL1 T5	chr3R	22929001	22946000	17000	1
mbtL1 T5	chr3R	24675001	24693000	18000	-1
mbtL1 T5	chr3R	27959001	28291000	332000	1
mbtL1 T5	chr3R	28339001	28502000	163000	1
mbtL1 T5	chr3R	32065001	32069000	4000	-2
mbtL1 T5	chr4	39001	230000	191000	-1
mbtL1 T5	chr4	1008001	1060000	52000	-2
mbtL2 T0	chr2L	3434001	3466000	32000	-1
mbtL2 T0	chr2L	4469001	4565000	96000	1
mbtL2 T0	chr2L	7705001	7723000	18000	1
mbtL2 T0	chr2L	7958001	7968000	10000	-1
mbtL2 T0	chr2R	6235001	6237000	2000	-1
mbtL2 T0	chr2R	14000001	14069000	69000	-1
mbtL2 T0	chr2R	15129001	15141000	12000	-1
mbtL2 T0	chr2R	15584001	15626000	42000	-1
mbtL2 T0	chr2R	18241001	18379000	138000	-1
mbtL2 T0	chr2R	22991001	23027000	36000	-1
mbtL2 T0	chr2R	23702001	23708000	6000	-1
mbtL2 T0	chr2R	24690001	24748000	58000	-1
mbtL2 T0	chr3L	1098001	1115000	17000	-1
mbtL2 T0	chr3L	2047001	2097000	50000	1
mbtL2 T0	chr3L	7822001	7872000	50000	-1
mbtL2 T0	chr3L	10661001	10745000	84000	-1
mbtL2 T0	chr3L	11968001	11998000	30000	1
mbtL2 T0	chr3L	17384001	17386000	2000	-1
mbtL2 T0	chr3L	22132001	22161000	29000	1
mbtL2 T0	chr3R	11372001	11377000	5000	-1
mbtL2 T0	chr3R	14375001	14381000	6000	-1
mbtL2 T0	chr3R	25398001	25402000	4000	-1
mbtL2 T0	chr3R	27960001	28291000	331000	1
mbtL2 T0	chr3R	28431001	28523000	92000	1
mbtL2 T0	chr3R	32063001	32069000	6000	1
mbtL2 T0	chrX	124001	265000	141000	1
mbtL2 T0	chrX	8400001	8402000	2000	-1
mbtL2 T0	chrX	10463001	10472000	9000	-1
mbtL2 T0	chrX	10605001	10608000	3000	-1
mbtL2 T0	chrX	11109001	11123000	14000	-1
mbtL2 T0	chrX	11587001	11596000	9000	-1
mbtL2 T0	chrX	11597001	11598000	1000	-1
mbtL2 T0	chrX	12688001	12716000	28000	-1
mbtL2 T0	chrX	20174001	20236000	62000	1
mbtL2 T10A	chr2L	1230001	1502000	272000	1
mbtL2 T10A	chr2L	4043001	4173000	130000	1
mbtL2 T10A	chr3L	1057001	1098000	41000	1
mbtL2 T10A	chr3R	9457001	9470000	13000	-1

mbtL2 T10A	chr3R	21317001	25398000	4081000	1
mbtL2 T10A	chr3R	25398001	25402000	4000	-1
mbtL2 T10A	chr3R	25402001	27960000	2558000	1
mbtL2 T10A	chr3R	27960001	28270000	310000	1
mbtL2 T10A	chr3R	28339001	28505000	166000	1
mbtL2 T10A	chr4	64001	174000	110000	-1
mbtL2 T10A	chrX	21167001	21185000	18000	-1
mbtL2 T10B	chr3L	1064001	1098000	34000	1
mbtL2 T10B	chr3L	8812001	8846000	34000	1
mbtL2 T10B	chr3L	9102001	9107000	5000	1
mbtL2 T10B	chr3L	22111001	22246000	135000	1
mbtL2 T10B	chr3R	21317001	25398000	4081000	1
mbtL2 T10B	chr3R	25398001	25402000	4000	-1
mbtL2 T10B	chr3R	25402001	27960000	2558000	1
mbtL2 T10B	chr3R	27960001	28270000	310000	1
mbtL2 T10B	chr3R	28430001	28562000	132000	1
mbtL2 T10B	chr4	120001	141000	21000	-1
mbtL2 T10B	chrX	11587001	11596000	9000	-1
mbtL2 T10B	chrX	21167001	21184000	17000	-1
mbtL2 T5	chr2L	2947001	2951000	4000	-1
mbtL2 T5	chr2L	4467001	4565000	98000	1
mbtL2 T5	chr2L	5014001	5018000	4000	-1
mbtL2 T5	chr2L	8827001	8832000	5000	-1
mbtL2 T5	chr2L	16491001	16623000	132000	-1
mbtL2 T5	chr2L	16758001	16782000	24000	1
mbtL2 T5	chr2R	18656001	18694000	38000	-1
mbtL2 T5	chr3L	5696001	5699000	3000	-1
mbtL2 T5	chr3L	8465001	8468000	3000	-1
mbtL2 T5	chr3L	12007001	12052000	45000	-1
mbtL2 T5	chr3L	14202001	14215000	13000	-1
mbtL2 T5	chr3L	17384001	17386000	2000	-1
mbtL2 T5	chr3R	5743001	5747000	4000	-1
mbtL2 T5	chr3R	11371001	11377000	6000	-1
mbtL2 T5	chr3R	19166001	19172000	6000	-1
mbtL2 T5	chr3R	27701001	27703000	2000	-1
mbtL2 T5	chr3R	28339001	28570000	231000	1
mbtL2 T5	chr3R	31578001	31580000	2000	-1
mbtL2 T5	chr4	801001	827000	26000	1
mbtL2 T5	chrX	124001	265000	141000	1
mbtL2 T5	chrX	267001	849000	582000	1
mbtL2 T5	chrX	1998001	2002000	4000	-1
mbtL2 T5	chrX	2567001	2599000	32000	1
mbtL2 T5	chrX	10466001	10471000	5000	-1
mbtL2 T5	chrX	11110001	11123000	13000	-1
mbtL2 T5	chrX	12688001	12693000	5000	-1
mbtL2 T5	chrX	16314001	16543000	229000	1
mbtL2 T5	chrX	19778001	19782000	4000	-1
mbtL2 T5	chrX	20392001	20411000	19000	-1
mbtL2 T5	chrX	21167001	21184000	17000	-1

Table S1.
Catalogue of CNVs found in the cohort.

Statistically significant (P < 0.05) over-representation of GO-Slim categories

GO term	REF	DUPLICATED	expected	Fold Enrichment	P value	GO dataset
**proteinaceous extracellular matrix (GO:0005578)**	**68**	**25**	**9.09**	**2.75**	**0.00728**	**CC**

GO term	REF	DELETED	expected	Fold Enrichment	P value	GO dataset
**nucleosome assembly (GO:0006334)**	**90**	**73**	**6.36**	**11.49**	**6.18E-48**	**BP**
chromatin assembly or disassembly (GO:0006333)	126	97	8.9	10.9	1.13E-62	BP
chromatin assembly (GO:0031497)	99	74	6.99	10.58	3.19E-46	BP
protein-DNA complex assembly (GO:0065004)	119	76	8.4	9.04	7.67E-43	BP
nucleosome organization (GO:0034728)	122	76	8.62	8.82	4.19E-42	BP
DNA packaging (GO:0006323)	155	78	10.95	7.13	6.80E-37	BP
protein-DNA complex subunit organization (GO:0071824)	158	79	11.16	7.08	3.28E-37	BP
DNA conformation change (GO:0071103)	170	80	12.01	6.66	6.83E-36	BP
chromatin organization (GO:0006325)	423	139	29.87	4.65	9.46E-47	BP
**DNA-templated transcription, initiation (GO:0006352)**	**102**	**31**	**7.2**	**4.3**	**1.09E-07**	**BP**
chromosome organization (GO:0051276)	608	153	42.94	3.56	4.90E-38	BP
cellular macromolecular complex assembly (GO:0034622)	404	96	28.53	3.36	8.94E-21	BP
macromolecular complex assembly (GO:0065003)	442	99	31.21	3.17	1.10E-19	BP
macromolecular complex subunit organization (GO:0043933)	521	106	36.79	2.88	3.37E-18	BP
nucleic acid-templated transcription (GO:0097659)	229	45	16.17	2.78	6.37E-06	BP
transcription, DNA-templated (GO:0006351)	229	45	16.17	2.78	6.37E-06	BP
RNA biosynthetic process (GO:0032774)	232	45	16.38	2.75	9.37E-06	BP
cellular component assembly (GO:0022607)	952	147	67.23	2.19	2.95E-15	BP
cellular component biogenesis (GO:0044085)	1100	156	77.68	2.01	4.00E-13	BP
nucleobase-containing compound biosynthetic process (GO:0034654)	392	54	27.68	1.95	1.39E-02	BP
organelle organization (GO:0006996)	1739	231	122.81	1.88	7.00E-18	BP
aromatic compound biosynthetic process (GO:0019438)	463	60	32.7	1.84	2.59E-02	BP
heterocycle biosynthetic process (GO:0018130)	464	60	32.77	1.83	2.76E-02	BP
**nuclear nucleosome (GO:0000788)**	**67**	**66**	**4.73**	**13.95**	**7.15E-49**	**CC**
nucleosome (GO:0000786)	125	117	8.83	13.25	3.31E-86	CC
DNA packaging complex (GO:0044815)	132	117	9.32	12.55	1.26E-83	CC
protein-DNA complex (GO:0032993)	145	119	10.24	11.62	1.96E-81	CC
nuclear chromatin (GO:0000790)	193	97	13.63	7.12	3.46E-47	CC
nuclear chromosome part (GO:0044454)	248	99	17.51	5.65	9.93E-40	CC
chromatin (GO:0000785)	341	132	24.08	5.48	1.53E-52	CC
nuclear chromosome (GO:0000228)	282	101	19.91	5.07	1.24E-36	CC
chromosomal part (GO:0044427)	528	143	37.29	3.84	2.38E-39	CC
chromosome (GO:0005694)	633	149	44.7	3.33	2.51E-34	CC

Most downstream GO terms are shown in bold
Only GO terms with less than 500 members are shown

REF = number of genes in the reference genome
DUPLICATED= number of genes amplified in the cohort analysed
DELETED= number of genes deleted in the cohort analysed
EXPECTED=numb er of genes theorically expected in the cohort
Fold Enrichment= experimental enrichment in the cohort analysed
P value= p-value after Bonferroni correction
GO dataset=BP: Biological Process; CC: cellular component

Table S2.
GO analyses of genes affected by CNVs.

sample	chromosome	position	mutation	alternative allele	Gene name	Flybase ID
lgl_T10	4	574985	C-A	0.10	*Ephrin*	FBgn0040324
lgl_T5	4	535708	C-T	0.23	*zfh2*	FBgn0004607
bratL1 T10	4	637790	C-G	0.25	*CG9935*	FBgn0039916
lgl_T5	4	738671	C-A	0.50	*bt*	FBgn0005666
lgl_T5	4	1164294	G-A	0.29	*Kif3C*	FBgn0039925
lgl_T5	4	878681	C-A	0.14	*unc-13*	FBgn0025726
lgl_T10	4	738671	C-A	0.47	*bt*	FBgn0005666
lgl_T5	4	1002712	G-T	0.11	*toy*	FBgn0019650
lgl_T10	4	618855	G-T	0.09	*Eph*	FBgn0025936
lgl_T10	4	73834	G-T	0.21	*pan*	FBgn0085432
lgl_T5	4	892562	C-A	0.11	*unc-13*	FBgn0025726
lgl_T5	4	169190	G-T	0.12	*CG33978*	FBgn0053978
bratL1 T10	4	335862	G-T	0.27	*PMCA*	FBgn0259214
bratL1 T10	4	333659	A-T	0.36	*PMCA*	FBgn0259214
mbtL2 T0	4	1163942	C-T	0.18	*Kif3C*	FBgn0039925
mbtL2 T10A	4	1163942	C-T	0.22	*Kif3C*	FBgn0039925
mbtL2 T10B	4	1163942	C-T	0.15	*Kif3C*	FBgn0039925
mbtL2 T10B	4	1163940	T-A	0.15	*Kif3C*	FBgn0039925
mbtL2 T5	4	1163942	C-T	0.12	*Kif3C*	FBgn0039925
mbtL2 T10A	4	1163940	T-A	0.22	*Kif3C*	FBgn0039925
mbtL2 T0	4	1163940	T-A	0.21	*Kif3C*	FBgn0039925
lgl_T5	4	156574	G-T	0.21		
mbtL1 T5	4	504071	C-A	0.14	*zfh2*	FBgn0004607
lgl_T10	4	48694	C-A	0.14	*ci*	FBgn0004859
bratL1 T5	4	637790	C-G	0.23	*CG9935*	FBgn0039916
bratL1 T10	4	333647	G-C	0.38	*PMCA*	FBgn0259214
bratL1 T0	4	637790	C-G	0.24	*CG9935*	FBgn0039916
aura T5	4	43468	G-T	0.24	*PlexB*	FBgn0025740
bratL1 T0	2L	22514717	G-T	0.22		
bratL1 T5	2L	15578994	A-T	0.16	*kek3*	FBgn0028370
bratL2 T0	2L	22505955	C-A	0.21		
bratL1 T10	2L	15334700	G-A	0.18	*esg*	FBgn0001981
mbtL1 T0	2L	13603198	G-A	0.20	*kuz*	FBgn0259984
mbtL2 T0	2L	11270130	A-G	0.21	*cana*	FBgn0040233
bratL1 T10	2L	6638680	G-C	0.46	*CG31635*	FBgn0051635
bratL2 T0	2L	8165845	G-T	0.16	*Piezo*	FBgn0264953
bratL2 T0	2L	13296308	G-T	0.19	*CG31729*	FBgn0051729
bratL1 T10	2L	22514717	G-T	0.14		
mbtL2 T0	2L	22513837	T-A	0.11		
aura T0	2L	11133734	C-A	0.11	*CG4751*	FBgn0032348
mbtL1 T0	2L	5926297	A-T	0.36	*dsf*	FBgn0015381
mbtL2 T10A	2L	10003188	C-A	0.14		
aura T0	2L	17738225	C-A	0.14	*CadN*	FBgn0015609
bratL1 T5	2L	5603482	G-A	0.11	*CG14010*	FBgn0031725
mbtL2 T10B	2L	23072321	C-A	0.12		
lgl_T0	2L	12661130	T-C	0.50	*pdm2*	FBgn0004394
lgl_T0	2L	16323817	C-A	0.14	*cact*	FBgn0000250
mbtL2 T10B	2L	13610750	T-A	0.63	*kuz*	FBgn0259984
mbtL2 T10A	2L	23231919	T-A	0.24		
mbtL2 T10A	2L	4627951	G-T	0.64		
aura T0	2L	6821763	G-T	0.14		
aura T0	2L	20761062	G-T	0.13	*dia*	FBgn0011202
mbtL2 T10A	2L	12947200	T-C	0.31		
mbtL2 T10A	2L	16566380	C-T	0.28	*CG42389*	FBgn0259735
mbtL2 T10B	2L	16566380	C-T	0.45	*CG42389*	FBgn0259735
bratL1 T10	2L	18169055	A-C	0.66	*CG42750*	FBgn0261804
mbtL1 T10	2L	12859565	G-A	0.16		
lgl_T0	2L	21789731	C-A	0.18	*CG31612*	FBgn0051612
mbtL2 T5	2L	6318382	C-A	0.07	*Ddr*	FBgn0053531
mbtL2 T5	2L	11418129	G-T	0.17		
mbtL2 T10B	2L	10016586	C-A	0.14	*CG13131*	FBgn0032175
mbtL1 T0	2L	21396770	C-T	0.14		

aura T0	2L	3721412	G-T	0.14	*Shaw*	FBgn0003386
mbtL2 T0	2L	15355355	C-A	0.14		
mbtL2 T10B	2L	19034083	C-G	0.53	*CG31800*	FBgn0051800
mbtL2 T5	2L	16528994	G-T	0.25	*CG5953*	FBgn0032587
mbtL2 T10A	2L	19034083	C-G	0.52	*CG31800*	FBgn0051800
mbtL2 T0	2L	4545625	A-T	0.15	*dp*	FBgn0053196
mbtL1 T0	2L	18840252	G-T	0.20	*CG31752*	FBgn0051752
mbtL1 T10	2L	5134543	C-T	0.50	*Msp300*	FBgn0261836
mbtL2 T10B	2L	4627951	G-T	0.66		
mbtL1 T5	2L	15909166	G-T	0.12	*beat-Ib*	FBgn0028645
mbtL1 T10	2L	17937168	C-A	0.07		
mbtL1 T0	2L	15429902	C-A	0.15		
mbtL1 T10	2L	14616591	C-A	0.19	*Adhr*	FBgn0000056
aura T0	2L	12811104	G-T	0.14		
mbtL1 T5	2L	12867252	A-G	0.64		
mbtL2 T0	2L	12859385	T-C	0.15		
lgl_T0	2L	8998259	G-T	0.14	*CG18661*	FBgn0040964
mbtL2 T0	2L	16848753	C-T	0.17		
lgl_T10	2L	8235218	G-T	0.11	*Pvr*	FBgn0032006
aura T5	2L	16308048	C-A	0.10	*Syx5*	FBgn0011708
mbtL1 T10	2L	16057468	C-A	0.11	*beat-Ia*	FBgn0013433
mbtL1 T0	2L	16848680	T-A	0.17	*CG13284*	FBgn0032614
lgl_T0	2L	19025401	C-A	0.12		
lgl_T10	2L	16196064	C-A	0.15	*CR44735*	FBgn0265948
mbtL2 T10B	2L	7318873	G-T	0.13		
lgl_T0	2L	17632510	C-A	0.13		
mbtL2 T10A	2L	5906221	G-T	0.16	*CR43808*	FBgn0264352
lgl_T10	2L	9852669	G-T	0.14	*nAChRalpha6*	FBgn0032151
mbtL1 T5	2L	6382764	C-A	0.16	*CG9507*	FBgn0031808
mbtL1 T0	2L	22604751	A-C	0.15		
mbtL1 T10	2L	19130554	G-T	0.11	*Aats-asn*	FBgn0086443
mbtL2 T5	2L	13988032	G-T	0.13	*rk*	FBgn0003255
bratL1 T5	2L	1765512	C-G	0.11		
mbtL2 T5	2L	7421927	C-A	0.13	*chm*	FBgn0028387
aura T5	2L	10742951	G-T	0.11	*CG6729*	FBgn0032296
mbtL2 T10B	2L	2729520	C-A	0.11	*CG31689*	FBgn0031449
aura T5	2L	12509753	G-T	0.12	*bun*	FBgn0259176
lgl_T0	2L	7496024	G-T	0.20	*Ziz*	FBgn0260486
lgl_T10	2L	3042841	G-T	0.14	*CG17261*	FBgn0031501
aura T5	2L	19096438	C-A	0.14	*Lim3*	FBgn0002023
mbtL1 T10	2L	1605912	T-G	1.00	*CG31935*	FBgn0051935
mbtL1 T5	2L	18239113	C-A	0.14	*CG42750*	FBgn0261804
mbtL1 T10	2L	6727404	C-A	0.10	*Liprin-alpha*	FBgn0046704
mbtL2 T5	2L	3008544	C-A	0.09	*CG17265*	FBgn0031488
lgl_T5	2L	17005370	C-A	0.13		
aura T5	2L	22414927	T-A	0.06		
lgl_T5	2L	8968031	C-A	0.13	*CG18088*	FBgn0032082
mbtL2 T5	2L	19889634	C-A	0.12	*sick*	FBgn0263873
lgl_T5	2L	22736853	C-A	0.09	*CG40006*	FBgn0058006
aura T5	2L	5785511	G-T	0.10	*CG9171*	FBgn0031738
mbtL2 T0	2L	17739428	G-T	0.10	*CadN*	FBgn0015609
mbtL2 T5	2L	18094238	C-A	0.15		
aura T0	2L	13687223	C-A	0.13	*CG31814*	FBgn0051814
aura T5	2L	7136791	G-T	0.14	*Pvf3*	FBgn0085407
mbtL2 T10B	2L	5569966	T-G	0.35		
lgl_T10	2L	5846261	G-T	0.10	*TrissinR*	FBgn0085410
mbtL2 T0	2L	1971630	G-T	0.18		
aura T5	2L	20402067	G-T	0.12	*CG10947*	FBgn0032857
mbtL2 T5	2L	18925481	G-T	0.11	*ssp3*	FBgn0032723
mbtL1 T5	2L	2164692	C-A	0.18	*aop*	FBgn0000097
mbtL1 T10	2L	3177601	G-T	0.13	*CG34393*	FBgn0085422
mbtL1 T5	2L	4207160	G-T	0.13	*ft*	FBgn0001075
aura T5	2L	14865373	C-A	0.24		

mbtL1 T10	2L	9162187	C-A	0.19	*CG32982*	FBgn0052982
lgl_T10	2L	4029816	G-T	0.16		
mbtL1 T5	2L	9662346	C-A	0.39		
mbtL2 T5	2L	2000478	C-A	0.11	*CR43464*	FBgn0263441
lgl_T5	2L	7996714	C-A	0.12	*CG7227*	FBgn0031970
lgl_T10	2L	21790754	G-T	0.20	*CG31612*	FBgn0051612
aura T5	2L	3718494	G-T	0.17	*Shaw*	FBgn0003386
mbtL1 T5	2L	13892282	C-A	0.10	*CenG1A*	FBgn0028509
aura T5	2L	2283287	C-A	0.12		
lgl_T10	2L	18902150	C-A	0.15		
mbtL2 T5	2L	12569192	C-A	0.10		
mbtL1 T5	2L	19917039	C-A	0.12	*sick*	FBgn0263873
mbtL1 T5	2L	8465486	G-T	0.13	*fu2*	FBgn0029173
mbtL1 T10	2L	7867185	G-T	0.15	*Proc*	FBgn0045038
mbtL2 T10A	2L	19698873	C-A	0.13	*Lar*	FBgn0000464
mbtL2 T10A	2L	13610750	T-A	0.37	*kuz*	FBgn0259984
lgl_T5	2L	10171027	C-A	0.14	*CG44153*	FBgn0265002
lgl_T5	2L	10822146	G-T	0.10	*Nos*	FBgn0011676
aura T5	2L	9643585	G-T	0.13	*Apoltp*	FBgn0032136
mbtL2 T5	2L	8692253	G-T	0.13	*Hnf4*	FBgn0004914
lgl_T5	2L	11771876	G-T	0.11		
mbtL2 T0	2L	5167200	G-A	0.07	*Msp300*	FBgn0261836
lgl_T10	2L	18189079	G-T	0.15	*CG42750*	FBgn0261804
lgl_T5	2L	5188458	C-A	0.13	*Msp300*	FBgn0261836
mbtL2 T10B	2L	1395091	C-A	1.00	*lea*	FBgn0002543
mbtL1 T5	2L	11124626	C-A	0.12	*Nup160*	FBgn0262647
aura T5	2L	7729285	G-T	0.17	*CG13796*	FBgn0031939
mbtL1 T5	2L	20507734	C-A	0.15		
lgl_T10	2L	16780514	C-A	0.16	*Mhc*	FBgn0264695
mbtL1 T10	2L	5701283	C-A	0.15		
lgl_T10	2L	20089233	C-A	0.12	*neb*	FBgn0004374
aura T5	2L	8648803	G-T	0.22	*Sema-1a*	FBgn0011259
mbtL2 T5	2L	3568071	C-A	0.15		
mbtL1 T5	2L	7318973	G-T	0.12		
lgl_T10	2L	20676632	G-T	0.44	*ik2*	FBgn0086657
mbtL1 T10	2L	2353346	C-A	0.13	*CG9967*	FBgn0031413
mbtL2 T5	2L	17216511	G-T	0.13	*beat-IIIc*	FBgn0032629
mbtL2 T0	2L	2506187	G-T	0.19		
mbtL1 T5	2L	10519810	G-T	0.16		
mbtL1 T5	2L	16435343	C-A	0.16	*CG5888*	FBgn0028523
mbtL2 T5	2L	8050308	A-G	0.08	*CG7466*	FBgn0031981
mbtL1 T5	2L	1023829	G-T	0.12	*IA-2*	FBgn0031294
mbtL1 T5	2L	21396770	C-T	0.14		
lgl_T5	2L	6169695	G-T	0.10		
aura T5	2L	11245559	G-T	0.10	*ab*	FBgn0264442
aura T5	2L	882247	G-T	0.13	*CG15824*	FBgn0031292
mbtL1 T10	2L	9662346	C-A	0.50		
mbtL2 T10B	2L	782424	G-T	0.12	*Gr21a*	FBgn0041250
lgl_T5	2L	21790342	G-T	0.24	*CG31612*	FBgn0051612
aura T5	2L	13603198	G-A	0.27	*kuz*	FBgn0259984
mbtL1 T10	2L	8353711	C-T	0.29	*Mur29B*	FBgn0051901
lgl_T10	2L	4984384	C-A	0.12	*CG34126*	FBgn0083962
mbtL1 T5	2L	22123489	G-T	0.17	*CG3651*	FBgn0032974
mbtL1 T5	2L	16910224	G-T	0.15	*ApepP*	FBgn0026150
mbtL1 T10	2L	7201379	C-A	0.10	*CG4502*	FBgn0031896
lgl_T5	2L	1831823	C-A	0.16	*c-cup*	FBgn0031367
lgl_T10	2L	19522764	G-T	0.13	*CG10132*	FBgn0032798
mbtL1 T5	2L	18704324	C-A	0.15	*CG10343*	FBgn0032703
aura T5	2L	16920244	G-T	0.15		
lgl_T5	2L	4372316	C-A	0.13	*Traf4*	FBgn0026319
lgl_T5	2L	20509111	G-T	0.11		
lgl_T10	2L	14354374	G-T	0.13	*l(2)34Fd*	FBgn0261535
aura T5	2L	17813247	G-T	0.14	*CadN2*	FBgn0262018

mbtL2 T0	2L	18187079	G-T	0.14	*CG42750*	FBgn0261804
lgl_T5	2L	7174612	G-T	0.12	*CR44610*	FBgn0265821
lgl_T5	2L	14431499	G-T	0.10		
lgl_T10	2L	14795756	C-A	0.10	*CG18420*	FBgn0028866
mbtL2 T5	2L	1219390	G-T	0.06	*CG42329*	FBgn0259229
lgl_T5	2L	9407672	C-A	0.13	*Shawl*	FBgn0085395
lgl_T10	2L	10343223	G-T	0.11	*Sps2*	FBgn0032224
aura T5	2L	12947490	G-T	0.18		
lgl_T5	2L	19644932	C-A	0.13	*Lar*	FBgn0000464
lgl_T5	2L	18890639	C-A	0.11		
lgl_T5	2L	15832728	C-A	0.12		
mbtL1 T5	2L	5034135	C-A	0.15	*Cg25C*	FBgn0000299
mbtL1 T5	2L	8876452	C-G	0.21		
mbtL2 T0	2L	22921707	C-T	0.16		
lgl_T5	2L	17403700	G-T	0.14	*CLIP-190*	FBgn0020503
lgl_T5	2L	13394969	G-T	0.10	*RpL24*	FBgn0032518
lgl_T10	2L	12447080	G-T	0.08	*CG5421*	FBgn0032434
mbtL1 T5	2L	22513837	T-A	0.13		
lgl_T5	2L	12529403	G-T	0.16	*bun*	FBgn0259176
mbtL1 T10	2L	10046624	C-T	0.23		
lgl_T10	2L	6758957	G-T	0.11	*CG11322*	FBgn0031856
lgl_T5	2L	5565259	C-A	0.15	*CR44794*	FBgn0266029
lgl_T10	2L	11184241	C-A	0.24	*Ca-beta*	FBgn0259822
lgl_T10	2L	12054946	G-T	0.14	*JhI-21*	FBgn0028425
mbtL1 T5	2L	20877996	C-A	0.17	*CG43739*	FBgn0263996
mbtL1 T5	2L	4618592	C-A	0.14		
aura T5	2L	9000001	C-A	0.11		
lgl_T5	2L	3908849	G-T	0.13	*fred*	FBgn0051774
mbtL1 T5	2L	3385477	G-T	0.12		
aura T5	2L	17260591	G-T	0.11	*beat-IIIc*	FBgn0032629
aura T5	2L	4058207	G-T	0.17	*ed*	FBgn0000547
lgl_T10	2L	17519129	G-T	0.10	*Dhc36C*	FBgn0013810
aura T5	2L	8063255	G-T	0.10	*poe*	FBgn0011230
lgl_T5	2L	14761558	C-A	0.13	*CG4691*	FBgn0028870
lgl_T10	2L	13184115	G-T	0.10	*CG5458*	FBgn0032478
aura T5	2L	2605259	G-T	0.13		
lgl_T5	2L	19211800	C-A	0.12		
mbtL1 T5	2L	19021434	C-A	0.16		
mbtL1 T5	2L	6699258	C-A	0.12	*Tsp*	FBgn0031850
mbtL2 T5	2L	778071	G-T	0.14		
lgl_T10	2L	6280342	G-T	0.18	*Ddr*	FBgn0053531
lgl_T5	2L	791335	G-T	0.17		
mbtL1 T5	2L	2478039	G-T	0.12		
aura T5	2L	4849321	C-A	0.10	*CG11927*	FBgn0031638
mbtL1 T5	2L	1336385	C-A	0.15		
mbtL2 T5	2L	14299606	G-T	0.12	*wb*	FBgn0261563
mbtL2 T0	2L	23231919	T-A	0.16		
aura T5	2L	20919922	G-T	0.14	*Mtp*	FBgn0266369
lgl_T5	2L	17781750	C-A	0.13		
aura T5	2L	13897458	G-T	0.13	*CenG1A*	FBgn0028509
mbtL1 T5	2L	13153132	G-T	0.14		
lgl_T5	2L	6455381	C-A	0.21	*retm*	FBgn0031814
lgl_T10	2L	20964212	C-A	0.12		
lgl_T5	2L	6729598	G-T	0.10	*Liprin-alpha*	FBgn0046704
lgl_T5	2L	15297099	G-T	0.12	*CR43764*	FBgn0264264
lgl_T10	2L	10808285	G-T	0.15	*Nos*	FBgn0011676
aura T5	2L	1152429	C-A	0.11	*capt*	FBgn0261458
mbtL1 T5	2L	17174997	C-A	0.21		
lgl_T5	2L	18413111	C-A	0.16		
lgl_T5	2L	20051184	C-A	0.10	*sNPF*	FBgn0032840
lgl_T10	2L	1976410	G-T	0.13	*CG15356*	FBgn0031377
aura T5	2L	2970166	C-A	0.10	*Rrp1*	FBgn0004584
mbtL2 T10B	2L	19292489	G-T	0.13		

lgl_T5	2L	2437128	G-T	0.17	*dpp*	FBgn0000490
lgl_T10	2L	1154472	G-T	0.19	*capt*	FBgn0261458
lgl_T10	2L	17034744	G-T	0.17		
lgl_T10	2L	8489433	C-A	0.12	*CG13088*	FBgn0032047
lgl_T5	2L	20990641	G-T	0.15	*CG42238*	FBgn0250867
mbtL1 T5	2L	21645893	G-T	0.17	*Ac3*	FBgn0023416
lgl_T10	2L	12694849	C-T	0.48	*CG5780*	FBgn0032446
lgl_T5	2L	13767360	G-T	0.15	*TM9SF4*	FBgn0028541
lgl_T5	2L	3153594	G-T	0.10	*Mad*	FBgn0011648
lgl_T5	2L	21231699	G-T	0.19	*CG8677*	FBgn0026577
mbtL2 T5	2L	11867946	A-G	0.60	*Pde1c*	FBgn0264815
aura T5	2L	19563622	C-A	0.13		
mbtL2 T10B	2L	5950257	C-A	0.10	*CG9044*	FBgn0031752
mbtL2 T10B	2L	1634009	G-T	0.17		
lgl_T10	2L	13636303	C-A	0.23	*kuz*	FBgn0259984
mbtL2 T10A	2L	1395091	C-A	1.00	*lea*	FBgn0002543
lgl_T10	2L	312630	G-T	0.10	*Plc21C*	FBgn0004611
mbtL2 T5	2L	12799435	C-A	0.21		
aura T5	2L	19323643	C-A	0.12	*CR45700*	FBgn0267264
lgl_T10	2L	4499816	G-T	0.11	*dp*	FBgn0053196
lgl_T5	2L	15022719	C-A	0.19	*GABA-B-R1*	FBgn0260446
lgl_T10	2L	1575930	C-A	0.21	*haf*	FBgn0261509
mbtL1 T5	2L	7758866	G-T	0.10	*CG46025*	FBgn0267689
aura T5	2L	18031892	C-A	0.14	*rdo*	FBgn0243486
mbtL1 T5	2L	7535618	C-A	0.15	*RapGAP1*	FBgn0264895
aura T5	2L	21244670	C-A	0.11	*Hr39*	FBgn0261239
lgl_T5	2L	22951488	C-A	0.15		
lgl_T10	2L	11459672	C-A	0.15		
mbtL1 T5	2L	2690371	G-T	0.12	*CG31690*	FBgn0051690
mbtL2 T5	2L	11627827	C-A	0.11		
lgl_T5	2L	1158243	C-A	0.12	*capt*	FBgn0261458
lgl_T5	2L	4575781	C-A	0.17	*dp*	FBgn0053196
aura T5	2L	4260933	G-T	0.13	*CR44080*	FBgn0264889
lgl_T5	2L	3393793	G-T	0.10		
mbtL1 T5	2L	19221271	C-A	0.19		
mbtL1 T10	2L	1799493	A-G	0.14		
aura T5	2L	4536493	A-G	0.13	*dp*	FBgn0053196
aura T5	2L	6266878	G-T	0.10	*Ddr*	FBgn0053531
lgl_T10	2L	3230307	T-C	0.13		
lgl_T5	2L	12998615	C-A	0.14	*CG12404*	FBgn0032465
aura T5	2L	374186	G-T	0.17		
mbtL1 T5	2L	2911229	C-A	0.15	*lilli*	FBgn0041111
aura T5	2L	21424884	C-T	0.05	*His4:CG31611*	FBgn0051611
lgl_T5	2L	2911938	C-A	0.21	*lilli*	FBgn0041111
lgl_T5	2L	11000157	G-T	0.11	*aub*	FBgn0000146
mbtL2 T5	2L	20066838	C-A	0.10	*bwa*	FBgn0045064
lgl_T5	2L	2732028	C-A	0.08	*CG31689*	FBgn0031449
lgl_T5	2L	3562148	G-T	0.19	*CR44309*	FBgn0265368
mbtL2 T10A	2L	16733686	A-T	0.37	*glu*	FBgn0015391
mbtL2 T10B	2L	16733686	A-T	0.47	*glu*	FBgn0015391
aura T5	2L	13113987	C-A	0.24		
lgl_T5	2L	15999048	G-T	0.20		
mbtL2 T5	2L	462487	G-T	0.10	*CG4297*	FBgn0031258
lgl_T10	2L	12859385	T-C	0.12		
lgl_T10	2L	4273917	G-T	0.17		
aura T5	2L	1309302	C-A	0.19		
lgl_T5	2L	1362998	G-T	0.12		
lgl_T5	2L	953904	G-T	0.12	*CG4341*	FBgn0028481
lgl_T10	2L	19056524	G-T	0.11	*CG10492*	FBgn0032748
lgl_T10	2L	17184201	C-A	0.15		
aura T5	2L	14045674	C-A	0.10	*nAChRalpha5*	FBgn0028875
lgl_T10	2L	704410	T-C	0.14	*ds*	FBgn0000497
aura T5	2L	191329	G-T	0.12	*spen*	FBgn0016977

aura T5	2L	16455517	C-A	0.17		
lgl_T5	2L	17928961	G-T	0.14		
lgl_T5	2L	2179374	C-A	0.18		
aura T5	2L	20548047	G-T	0.13		
lgl_T10	2L	871737	G-T	0.11	*PNUTS*	FBgn0053526
lgl_T10	2L	464210	G-T	0.12	*CG4297*	FBgn0031258
mbtL2 T10B	2L	5711209	G-T	0.13	*CG7236*	FBgn0031730
mbtL2 T5	2L	16670067	C-A	0.14	*Trpgamma*	FBgn0032593
mbtL1 T5	2L	9989457	C-A	0.13	*bib*	FBgn0000180
aura T5	2L	5923869	G-T	0.10		
lgl_T5	2L	20737695	C-A	0.10	*Fs(2)Ket*	FBgn0262743
lgl_T5	2L	1967443	C-A	0.10	*erm*	FBgn0031375
lgl_T5	2L	12813002	C-A	0.13		
lgl_T10	2L	1352396	C-A	0.13	*CG5556*	FBgn0031332
lgl_T5	2L	12663326	C-A	0.12	*pdm2*	FBgn0004394
mbtL1 T5	2L	14025900	G-T	0.16		
aura T5	2L	6079093	G-T	0.16	*CR9162*	FBgn0031778
mbtL1 T5	2L	3042473	C-A	0.19		
lgl_T10	2L	13397957	C-A	0.10	*CG10859*	FBgn0032520
lgl_T5	2L	12037821	C-A	0.18	*Tom70*	FBgn0032397
lgl_T10	2L	8615723	G-T	0.13	*Sema-1a*	FBgn0011259
mbtL1 T5	2L	11244479	C-A	0.10	*ab*	FBgn0264442
lgl_T5	2L	18144558	C-A	0.13	*Socs36E*	FBgn0041184
mbtL1 T5	2L	9850967	C-A	0.10	*nAChRalpha6*	FBgn0032151
lgl_T5	2L	13521458	A-C	0.50	*B4*	FBgn0023407
mbtL2 T10A	2L	16848753	C-T	0.13		
aura T5	2L	1480701	G-T	0.16		
lgl_T5	2L	2548551	G-T	0.15		
aura T5	2L	21029413	G-T	0.11	*CG42238*	FBgn0250867
aura T5	2L	2711618	G-T	0.10	*CG31690*	FBgn0051690
lgl_T5	2L	15413361	C-A	0.14		
mbtL2 T5	2L	7527229	G-T	0.08	*RapGAP1*	FBgn0264895
lgl_T10	2L	10537862	C-A	0.12		
aura T5	2L	17363518	C-A	0.13	*CG31804*	FBgn0051804
lgl_T5	2L	5665888	C-A	0.19	*Hsp60C*	FBgn0031728
mbtL1 T5	2L	5134543	C-T	0.50	*Msp300*	FBgn0261836
lgl_T10	2L	11598012	C-A	0.15	*kek2*	FBgn0015400
lgl_T10	2L	13734717	C-A	0.21		
lgl_T5	2L	18025430	C-A	0.20	*rdo*	FBgn0243486
aura T5	2L	11341572	G-T	0.13		
mbtL2 T10A	2L	13706347	G-C	0.41	*CG31814*	FBgn0051814
mbtL2 T10B	2L	13706347	G-C	0.38	*CG31814*	FBgn0051814
lgl_T10	2L	6374972	G-T	0.29	*slam*	FBgn0043854
lgl_T10	2L	13897458	G-T	0.11	*CenG1A*	FBgn0028509
lgl_T10	2L	556383	G-T	0.21	*rempA*	FBgn0260933
mbtL1 T5	2L	22604751	A-C	0.15		
lgl_T5	2L	20599966	G-T	0.13	*CR44909*	FBgn0266214
lgl_T5	2L	11090024	G-T	0.13	*CG16854*	FBgn0032338
lgl_T5	2L	19733269	C-A	0.10	*CG10462*	FBgn0032815
lgl_T10	2L	11683621	G-T	0.11		
lgl_T5	2L	11909240	C-A	0.14	*Pde1c*	FBgn0264815
lgl_T10	2L	13268604	C-A	0.12	*CG44085*	FBgn0264894
aura T5	2L	4345122	G-T	0.10	*Atet*	FBgn0020762
lgl_T5	2L	13986627	C-A	0.12	*rk*	FBgn0003255
lgl_T10	2L	5961770	G-T	0.10		
aura T5	2L	11423088	G-T	0.16		
mbtL2 T5	2L	298571	G-T	0.14	*Pi3K21B*	FBgn0020622
lgl_T10	2L	21040630	C-A	0.19	*CG42238*	FBgn0250867
lgl_T5	2L	9517289	G-T	0.18	*CG33298*	FBgn0032120
lgl_T5	2L	477975	C-A	0.13	*cbt*	FBgn0043364
mbtL2 T10B	2L	4700102	G-T	0.42	*CG34351*	FBgn0085380
aura T5	2L	1552238	G-T	0.19		
mbtL1 T5	2L	9732510	G-T	0.12	*Nckx30C*	FBgn0028704

lgl_T10	2L	13804469	A-G	0.12	*CAH1*	FBgn0027844
mbtL1 T0	2L	22671697	T-C	0.12	*CG40006*	FBgn0058006
lgl_T5	2L	2033152	G-T	0.11	*CG42296*	FBgn0259192
aura T5	2L	20612843	C-A	0.13	*CR44909*	FBgn0266214
lgl_T5	2L	13903281	G-T	0.10	*Smg5*	FBgn0019890
lgl_T10	2L	1217498	C-A	0.12	*CR43263*	FBgn0262944
lgl_T5	2L	12137216	G-T	0.22		
mbtL1 T5	2L	6760997	G-T	0.17	*CG11321*	FBgn0031857
lgl_T5	2L	21291511	G-T	0.10	*Gr39a*	FBgn0264556
lgl_T5	2L	403074	G-T	0.12		
aura T5	2L	1367731	G-T	0.13		
lgl_T5	2L	19788600	C-A	0.11	*CdGAPr*	FBgn0032821
lgl_T5	2L	13822049	C-A	0.12	*b*	FBgn0000153
lgl_T10	2L	9905365	G-T	0.11	*CG13124*	FBgn0032156
lgl_T10	2L	1714585	G-T	0.12	*frtz*	FBgn0086698
lgl_T10	2L	17571395	C-A	0.12		
lgl_T10	2L	14847968	G-T	0.12	*CG15279*	FBgn0028886
lgl_T5	2L	11824073	C-A	0.10	*Pde1c*	FBgn0264815
lgl_T10	2L	4116271	C-A	0.15		
lgl_T10	2L	10394795	C-A	0.12	*Mdh1*	FBgn0262782
mbtL2 T10B	2L	6000986	G-T	0.11	*Tsp26A*	FBgn0031760
lgl_T5	2L	11137571	G-T	0.13	*hgo*	FBgn0040211
lgl_T5	2L	17479839	G-T	0.09	*fws*	FBgn0024689
lgl_T5	2L	7220659	C-A	0.13	*ico*	FBgn0263133
lgl_T10	2L	11245983	C-A	0.12	*ab*	FBgn0264442
mbtL1 T5	2L	4249252	G-T	0.12	*CR44059*	FBgn0264868
lgl_T5	2L	3193695	G-T	0.10	*CG34393*	FBgn0085422
lgl_T10	2L	10433913	C-A	0.11	*CG5337*	FBgn0032249
mbtL2 T5	2L	12859385	T-C	0.10		
lgl_T10	2L	11498305	G-T	0.10	*CG6488*	FBgn0032361
mbtL1 T5	2L	2728478	G-T	0.10	*CG31689*	FBgn0031449
lgl_T5	2L	12075337	G-T	0.14		
lgl_T5	2L	1208070	G-T	0.12		
lgl_T10	2L	1612791	C-A	0.15	*RFeSP*	FBgn0021906
mbtL1 T5	2L	5171171	T-A	0.31	*Msp300*	FBgn0261836
mbtL1 T5	2L	7795101	C-A	0.13	*LKR*	FBgn0025687
lgl_T5	2L	343984	C-A	0.15	*Plc21C*	FBgn0004611
lgl_T5	2L	14797133	G-T	0.12		
lgl_T10	2L	1647893	C-A	0.17		
lgl_T10	2L	4064676	C-A	0.17	*ed*	FBgn0000547
lgl_T5	2L	9441557	C-A	0.09	*numb*	FBgn0002973
lgl_T10	2L	5879861	G-T	0.17		
lgl_T10	2L	20122366	G-T	0.15		
lgl_T5	2L	17433216	C-A	0.27		
lgl_T10	2L	899498	G-T	0.10	*Lsp1beta*	FBgn0002563
lgl_T5	2L	18461587	G-T	0.22	*let-7-C*	FBgn0263049
aura T5	2L	8088795	C-A	0.24	*Bsg*	FBgn0261822
aura T5	2L	5946106	C-A	0.08	*Gpdh*	FBgn0001128
mbtL1 T5	2L	19242287	C-A	0.12		
lgl_T5	2L	18434092	C-A	0.21	*CR44488*	FBgn0265681
mbtL2 T5	2L	12820282	C-A	0.07	*kek1*	FBgn0015399
mbtL1 T5	2L	17195333	G-T	0.15	*beat-IIIc*	FBgn0032629
lgl_T10	2L	724181	G-T	0.11		
lgl_T10	2L	11202675	G-T	0.10	*Ca-beta*	FBgn0259822
lgl_T5	2L	13839026	C-A	0.11	*CenG1A*	FBgn0028509
lgl_T10	2L	1662236	C-A	0.14	*chinmo*	FBgn0086758
lgl_T5	2L	12677460	G-T	0.18	*pdm2*	FBgn0004394
mbtL2 T5	2L	8704792	G-T	0.13	*Hnf4*	FBgn0004914
lgl_T5	2L	1170690	G-T	0.14	*CG31922*	FBgn0051922
lgl_T5	2L	16010594	C-A	0.17	*beat-Ic*	FBgn0028644
lgl_T10	2L	20688134	G-T	0.12	*Hr38*	FBgn0014859
lgl_T5	2L	15307728	G-T	0.24		
lgl_T5	2L	13404890	G-T	0.10	*CG7110*	FBgn0032521

lgl_T10	2L	322247	C-A	0.10	*Plc21C*	FBgn0004611
lgl_T5	2L	799464	G-T	0.14		
lgl_T5	2L	2555976	C-A	0.17	*CR44113*	FBgn0264944
aura T5	2L	381150	C-A	0.11	*al*	FBgn0000061
lgl_T5	2L	18150079	G-T	0.11	*Socs36E*	FBgn0041184
mbtL2 T10B	2L	4705227	G-C	0.40	*CG34351*	FBgn0085380
mbtL1 T5	2L	19026362	C-A	0.10		
lgl_T10	2L	729050	T-G	0.13	*Hsp60B*	FBgn0011244
lgl_T10	2L	5966563	G-T	0.12	*CG13992*	FBgn0031756
lgl_T5	2L	12141054	G-T	0.14		
aura T5	2L	9647060	G-T	0.13	*Apoltp*	FBgn0032136
lgl_T10	2L	12698150	C-A	0.10	*PICK1*	FBgn0032447
lgl_T5	2L	15025606	C-A	0.10	*GABA-B-R1*	FBgn0260446
lgl_T10	2L	6760941	C-T	0.11	*CG11321*	FBgn0031857
bratL1 T0	2L	22514754	C-G	0.14		
mbtL2 T5	2L	8050329	A-G	0.07	*CG7466*	FBgn0031981
mbtL1 T0	2L	21396790	T-C	0.13		
mbtL2 T0	2L	22921723	T-A	0.14		
mbtL2 T10A	2L	13706363	A-C	0.30	*CG31814*	FBgn0051814
mbtL2 T10B	2L	13706363	A-C	0.47	*CG31814*	FBgn0051814
bratL1 T0	2L	22514726	T-C	0.17		
bratL1 T10	2L	22514726	T-C	0.13		
mbtL2 T10A	2L	13706353	A-T	0.38	*CG31814*	FBgn0051814
mbtL2 T10B	2L	13706353	A-T	0.44	*CG31814*	FBgn0051814
mbtL2 T0	2L	23231923	A-C	0.16		
mbtL2 T10A	2L	23231923	A-C	0.21		
bratL1 T0	2L	22514756	A-G	0.14		
mbtL2 T0	2L	5167202	A-G	0.07	*Msp300*	FBgn0261836
lgl_T0	2L	12661131	G-A	0.50	*pdm2*	FBgn0004394
lgl_T10	2L	6374973	A-T	0.29	*slam*	FBgn0043854
mbtL1 T10	2L	1605913	C-G	1.00	*CG31935*	FBgn0051935
mbtL1 T10	2L	10046625	A-C	0.23		
mbtL2 T0	2L	1117278	C-A	0.13	*Pino*	FBgn0016926
lgl_T10	2L	82344	C-A	0.12		
lgl_T5	2L	308192	G-T	0.15	*Plc21C*	FBgn0004611
mbtL2 T10B	2L	282807	C-A	0.11	*CG11601*	FBgn0031244
mbtL2 T5	2L	217718	G-T	0.15	*kis*	FBgn0266557
bratL2 T5	2L	22480104	G-A	0.09		
mbtL1 T0	2L	1799493	A-G	0.19		
mbtL1 T10	2L	560192	C-A	0.14	*CG2789*	FBgn0031263
lgl_T0	2L	6416007	G-T	0.12	*Tig*	FBgn0011722
bratL2 T0	2L	2299247	A-T	0.80		
aura T0	2L	1199537	C-A	0.13	*CG43348*	FBgn0263080
bratL2 T0	2R	16702426	T-G	0.20	*CG30463*	FBgn0050463
bratL2 T5	2R	15757758	G-T	0.24	*CG30089*	FBgn0050089
mbtL2 T10B	2R	23570552	C-A	0.12	*apt*	FBgn0015903
aura T0	2R	23378165	G-T	0.19	*CG13551*	FBgn0040660
aura T0	2R	16026552	G-T	0.13	*casp*	FBgn0034068
bratL1 T5	2R	15268463	G-T	0.19	*Trpm*	FBgn0265194
mbtL1 T0	2R	10153857	C-A	0.21	*CR45133*	FBgn0266626
bratL1 T10	2R	16208003	A-T	0.80	*mrj*	FBgn0034091
mbtL2 T0	2R	10428530	C-T	0.31		
mbtL2 T0	2R	20225957	A-T	0.36		
bratL1 T5	2R	8498095	G-T	0.16	*mtt*	FBgn0050361
lgl_T0	2R	22457942	C-A	0.19	*CG10384*	FBgn0034731
mbtL2 T10A	2R	10997140	C-A	0.42	*luna*	FBgn0040765
mbtL2 T10B	2R	11002247	C-G	0.55	*luna*	FBgn0040765
mbtL2 T5	2R	16172922	C-A	0.11	*Ptp52F*	FBgn0034085
bratL1 T10	2R	19630716	G-C	0.76	*sm*	FBgn0003435
mbtL1 T5	2R	22334831	C-A	0.15	*Liprin-gamma*	FBgn0034720
mbtL2 T10A	2R	16310139	A-G	0.41	*Nox*	FBgn0085428
aura T0	2R	3763504	C-A	0.06		
lgl_T0	2R	16857343	C-A	0.33	*Dek*	FBgn0026533

mbtL2 T10A	2R	24084142	C-A	0.15	*CG16786*	FBgn0034974
lgl_T0	2R	11580358	G-T	0.15	*tou*	FBgn0033636
lgl_T5	2R	4151027	G-C	0.10		
mbtL1 T10	2R	4957226	C-A	0.13	*Ir41a*	FBgn0040849
mbtL2 T10A	2R	19159266	C-A	0.13	*ena*	FBgn0000578
mbtL2 T10B	2R	15749815	G-T	0.11	*CG30089*	FBgn0050089
bratL2 T0	2R	3133785	A-C	0.14	*CG42596*	FBgn0260995
bratL1 T10	2R	7744259	G-C	0.61	*CG45093*	FBgn0266526
lgl_T10	2R	3052267	A-G	0.12		
mbtL2 T5	2R	6123461	C-A	0.11	*EcR*	FBgn0000546
mbtL1 T10	2R	7347678	G-T	0.09	*Dscam1*	FBgn0033159
bratL1 T10	2R	3678239	A-T	0.17		
mbtL1 T0	2R	12448874	C-A	0.14	*Dyb*	FBgn0033739
mbtL2 T10A	2R	6553006	G-A	0.47	*jing*	FBgn0086655
mbtL1 T10	2R	17450570	G-A	0.11	*mthl3*	FBgn0028956
mbtL2 T10B	2R	6418424	T-C	0.25		
mbtL1 T0	2R	3515184	G-A	0.20		
mbtL1 T5	2R	24425184	G-T	0.12	*prom*	FBgn0259210
lgl_T0	2R	8533031	C-A	0.18		
mbtL2 T10B	2R	13024473	T-A	0.20		
aura T5	2R	17966770	C-A	0.11		
mbtL2 T5	2R	8951074	G-T	0.12	*CG8229*	FBgn0033356
mbtL1 T10	2R	23208057	G-T	0.16		
bratL1 T5	2R	17171750	C-A	0.17		
aura T5	2R	15107346	C-A	0.15		
aura T5	2R	6054357	G-T	0.13	*CCHa2-R*	FBgn0033058
mbtL2 T10B	2R	25242244	G-A	0.50	*CG9380*	FBgn0035094
mbtL2 T5	2R	22361829	T-G	0.12	*Liprin-gamma*	FBgn0034720
mbtL1 T10	2R	12756278	G-T	0.13	*Taz*	FBgn0026619
mbtL1 T10	2R	15246371	C-A	0.16		
mbtL1 T5	2R	5466943	G-T	0.11		
mbtL1 T0	2R	16836170	G-T	0.13	*CR43650*	FBgn0263659
bratL2 T5	2R	4730537	T-C	0.10	*CR40282*	FBgn0039979
mbtL2 T10A	2R	3418096	C-G	0.64		
mbtL1 T10	2R	8914722	G-T	0.14	*lin*	FBgn0002552
mbtL2 T0	2R	1532992	C-T	0.28		
bratL1 T10	2R	5150998	C-A	0.59	*Atf6*	FBgn0033010
mbtL1 T10	2R	2057804	C-A	0.19	*CG17684*	FBgn0263780
aura T0	2R	8436367	C-A	0.64		
mbtL2 T5	2R	23750612	T-C	0.11		
aura T5	2R	2746939	G-T	0.15		
mbtL2 T5	2R	19002911	C-A	0.12	*tn*	FBgn0265356
aura T0	2R	6986737	C-A	0.13	*CG17002*	FBgn0033122
mbtL2 T0	2R	14431662	G-T	0.17	*phyl*	FBgn0013725
mbtL2 T10A	2R	1845581	C-T	0.27		
mbtL1 T10	2R	19179254	C-A	0.18	*hppy*	FBgn0263395
mbtL2 T0	2R	13117491	T-G	0.12	*CG13325*	FBgn0033792
bratL2 T0	2R	17938687	G-T	0.23		
mbtL1 T5	2R	12909762	C-A	0.13		
mbtL1 T10	2R	10093660	G-T	0.11	*CG2269*	FBgn0033484
aura T5	2R	23277888	G-A	0.14		
mbtL2 T10A	2R	25242244	G-A	0.62	*CG9380*	FBgn0035094
lgl_T0	2R	12731687	G-T	0.12	*CG42663*	FBgn0261545
lgl_T10	2R	10818792	G-T	0.11	*dgo*	FBgn0086898
bratL1 T10	2R	8854704	T-G	0.37	*sns*	FBgn0024189
mbtL2 T10B	2R	2948001	C-A	0.10	*Scp1*	FBgn0020908
mbtL2 T0	2R	11529006	G-T	0.14		
mbtL1 T5	2R	11227979	G-T	0.13	*metro*	FBgn0050021
lgl_T10	2R	6093173	C-A	0.14	*EcR*	FBgn0000546
mbtL2 T0	2R	2589202	A-T	0.25		
mbtL2 T5	2R	12269735	G-T	0.16	*Cam*	FBgn0000253
mbtL1 T10	2R	21276043	C-T	0.24	*Rgk3*	FBgn0085426
mbtL1 T0	2R	18545840	C-A	0.19	*Rgk2*	FBgn0085419

mbtL2 T0	2R	3622914	A-C	0.22		
mbtL1 T5	2R	18536162	G-T	0.15		
lgl_T5	2R	11683160	G-T	0.11	*Buffy*	FBgn0040491
mbtL1 T10	2R	11115708	C-A	0.13	*luna*	FBgn0040765
mbtL2 T5	2R	24772353	G-T	0.09	*Eps-15*	FBgn0035060
mbtL2 T10B	2R	1532992	C-T	0.20		
mbtL1 T5	2R	3515184	G-A	0.36		
aura T5	2R	19694056	C-A	0.10	*CR44506*	FBgn0265699
aura T0	2R	4730585	A-G	0.14	*CR40282*	FBgn0039979
mbtL1 T0	2R	15227621	T-G	0.09	*Fs*	FBgn0259878
mbtL2 T5	2R	9908420	G-T	0.12	*FMRFa*	FBgn0000715
mbtL2 T10A	2R	11949910	A-G	0.44	*CG13188*	FBgn0033668
mbtL1 T5	2R	7587713	C-A	0.20		
lgl_T5	2R	16952682	C-A	0.10	*mute*	FBgn0085444
mbtL1 T5	2R	16680504	G-T	0.13	*CG30463*	FBgn0050463
lgl_T10	2R	4431215	G-T	0.10	*Gprk1*	FBgn0260798
aura T5	2R	21510321	C-A	0.11	*MESK2*	FBgn0043070
lgl_T5	2R	20989559	C-A	0.15	*CG3216*	FBgn0034568
lgl_T10	2R	11920107	C-A	0.13		
mbtL1 T10	2R	13617644	G-C	0.57		
bratL1 T10	2R	9704899	C-A	0.16	*CG30002*	FBgn0260474
aura T5	2R	4350672	C-A	0.14	*Gprk1*	FBgn0260798
mbtL2 T5	2R	17020761	C-A	0.12	*Dark*	FBgn0263864
mbtL1 T0	2R	19388466	C-A	0.21	*CG10081*	FBgn0034441
mbtL2 T10A	2R	12775399	T-C	0.36		
lgl_T5	2R	5111243	G-T	0.15		
lgl_T10	2R	8983989	G-T	0.10	*CG8213*	FBgn0033359
lgl_T10	2R	14614786	C-A	0.10	*Su(var)2-HP2*	FBgn0026427
lgl_T0	2R	17653322	C-A	0.14	*CCHa1-R*	FBgn0050106
lgl_T5	2R	13884249	G-T	0.13	*shot*	FBgn0013733
mbtL1 T5	2R	10157274	G-T	0.13	*mlt*	FBgn0265512
aura T5	2R	9949891	G-T	0.11	*Mef2*	FBgn0011656
lgl_T10	2R	19043811	C-A	0.12		
mbtL2 T10A	2R	20944475	A-G	0.50	*Act57B*	FBgn0000044
lgl_T10	2R	22117889	G-T	0.13	*Vrp1*	FBgn0243516
lgl_T5	2R	10258453	G-T	0.11	*CG12911*	FBgn0033501
lgl_T5	2R	19473357	C-A	0.15	*par-1*	FBgn0260934
aura T5	2R	12721461	C-A	0.13	*CG42663*	FBgn0261545
mbtL1 T0	2R	13118953	C-A	0.12	*CG13325*	FBgn0033792
mbtL1 T0	2R	17503221	A-C	0.23	*CG43164*	FBgn0262720
mbtL2 T0	2R	4288068	T-C	0.15		
mbtL2 T10B	2R	4288068	T-C	0.14		
mbtL1 T10	2R	19837942	G-T	0.11		
lgl_T5	2R	20121251	G-T	0.14		
lgl_T5	2R	15464887	G-T	0.11	*CG43729*	FBgn0263980
mbtL1 T0	2R	14261831	G-T	0.14	*CG8547*	FBgn0033919
lgl_T10	2R	17663262	C-A	0.13	*CCHa1-R*	FBgn0050106
aura T5	2R	3383315	G-C	0.11		
lgl_T10	2R	23871712	C-A	0.12	*ken*	FBgn0011236
mbtL1 T5	2R	25163157	A-T	0.14		
lgl_T5	2R	7059540	G-T	0.12	*pgant3*	FBgn0027558
lgl_T10	2R	16816494	G-T	0.13		
mbtL1 T5	2R	15321839	G-T	0.14	*CG8155*	FBgn0034009
mbtL1 T5	2R	8623134	G-T	0.12	*Cirl*	FBgn0033313
lgl_T10	2R	5033482	G-T	0.16		
mbtL1 T5	2R	6065759	G-T	0.11	*CCHa2-R*	FBgn0033058
mbtL2 T10A	2R	20225957	A-T	0.50		
mbtL2 T10A	2R	4207375	G-A	0.17		
aura T0	2R	8991786	G-C	0.83		
aura T5	2R	12049435	G-T	0.12	*Drep-1*	FBgn0024732
lgl_T5	2R	6437682	C-T	0.28		
mbtL2 T5	2R	17556642	C-A	0.60		
mbtL2 T5	2R	20695298	C-A	0.12		

mbtL1 T10	2R	23730565	G-T	0.15	*Sesn*	FBgn0034897
aura T0	2R	5656458	C-A	0.15	*Not3*	FBgn0033029
mbtL1 T0	2R	13617644	G-C	0.54		
mbtL1 T5	2R	19034265	G-T	0.13		
mbtL1 T5	2R	14110492	C-A	0.10	*Prosap*	FBgn0040752
lgl_T0	2R	13589584	C-A	0.19	*CG45088*	FBgn0266489
lgl_T5	2R	17577430	G-T	0.10	*Dcr-2*	FBgn0034246
lgl_T5	2R	18789781	C-A	0.10	*CG15080*	FBgn0034391
mbtL1 T5	2R	17450570	G-A	0.17	*mthl3*	FBgn0028956
aura T5	2R	8991786	G-C	0.67		
mbtL2 T10B	2R	3418096	C-G	0.53		
mbtL2 T5	2R	25242244	G-A	0.45	*CG9380*	FBgn0035094
mbtL1 T10	2R	17917200	G-T	0.10	*Elk*	FBgn0011589
mbtL1 T5	2R	14710818	C-A	0.17	*mspo*	FBgn0020269
lgl_T5	2R	5808748	G-T	0.14	*dpr12*	FBgn0085414
mbtL1 T5	2R	6514717	C-A	0.15	*jing*	FBgn0086655
lgl_T5	2R	15910715	G-T	0.16	*sli*	FBgn0264089
mbtL2 T5	2R	19440881	G-T	0.11	*Spt5*	FBgn0040273
mbtL1 T5	2R	15756628	C-A	0.10	*CG30089*	FBgn0050089
lgl_T10	2R	3485296	C-A	0.17		
mbtL1 T5	2R	13341802	G-T	0.16	*CR44206*	FBgn0265104
aura T5	2R	11140982	C-A	0.15	*CG13229*	FBgn0033579
aura T5	2R	20123719	G-T	0.16		
mbtL2 T5	2R	6553006	G-A	0.51	*jing*	FBgn0086655
lgl_T10	2R	19875222	G-T	0.12		
mbtL1 T5	2R	8013252	G-T	0.11	*CG14762*	FBgn0033250
aura T0	2R	5150506	G-T	0.10	*Atf6*	FBgn0033010
lgl_T5	2R	18256744	C-A	0.13		
lgl_T5	2R	14743959	G-T	0.21	*CG12858*	FBgn0033958
lgl_T10	2R	12595629	G-T	0.24	*CR43909*	FBgn0264510
lgl_T5	2R	23930012	C-A	0.10	*Ca-P60A*	FBgn0263006
lgl_T10	2R	15827762	G-T	0.18	*Poxn*	FBgn0003130
lgl_T10	2R	488366	A-G	0.11	*CG45781*	FBgn0267428
mbtL2 T5	2R	10834544	G-T	0.13	*CG12344*	FBgn0033558
lgl_T10	2R	19443985	C-A	0.14		
lgl_T10	2R	20542403	C-A	0.14		
lgl_T5	2R	12316394	G-T	0.25	*CG30046*	FBgn0050046
mbtL1 T10	2R	20222928	G-T	0.14		
lgl_T10	2R	9430851	G-T	0.10	*Pdk*	FBgn0017558
mbtL2 T10A	2R	19542992	A-T	0.15	*sm*	FBgn0003435
lgl_T10	2R	20935078	G-T	0.11	*Rx*	FBgn0020617
mbtL2 T10B	2R	16310139	A-G	0.46	*Nox*	FBgn0085428
lgl_T5	2R	22403558	G-T	0.12		
lgl_T10	2R	15055692	C-A	0.17	*CG33467*	FBgn0053467
lgl_T0	2R	13102654	C-A	0.12		
mbtL2 T10A	2R	445490	G-C	0.56	*CG45781*	FBgn0267428
mbtL2 T10B	2R	445490	G-C	0.63	*CG45781*	FBgn0267428
aura T5	2R	6418376	T-A	0.29		
aura T5	2R	21981546	C-A	0.11		
aura T5	2R	10710610	C-A	0.17	*stan*	FBgn0024836
aura T5	2R	11498300	C-A	0.17	*inv*	FBgn0001269
lgl_T5	2R	21846214	C-A	0.11		
aura T5	2R	6764929	C-A	0.16	*Epac*	FBgn0085421
lgl_T5	2R	9116335	C-A	0.17	*Su(var)2-10*	FBgn0003612
lgl_T10	2R	6437682	C-T	0.26		
mbtL1 T5	2R	9268546	G-T	0.14	*Pkn*	FBgn0020621
lgl_T5	2R	8165514	C-A	0.11	*Rs1*	FBgn0021995
lgl_T5	2R	8573917	G-T	0.13	*CG42326*	FBgn0259226
mbtL2 T0	2R	11865598	G-A	0.11	*Damm*	FBgn0033659
aura T5	2R	8381426	G-T	0.13	*pdm3*	FBgn0261588
lgl_T10	2R	6837642	G-T	0.17	*CG15236*	FBgn0033108
lgl_T10	2R	12973680	G-T	0.18	*Psc*	FBgn0005624
lgl_T5	2R	24282690	C-A	0.11	*nvy*	FBgn0005636

lgl_T5	2R	14213015	G-T	0.08		
lgl_T5	2R	21324932	C-A	0.12	*dom*	FBgn0020306
mbtL1 T5	2R	3840231	C-A	0.19		
lgl_T10	2R	22966383	C-A	0.12		
lgl_T10	2R	8081112	G-T	0.10	*slv*	FBgn0025469
lgl_T10	2R	15370828	C-A	0.10	*Pms2*	FBgn0011660
mbtL2 T10B	2R	1845581	C-T	0.29		
aura T5	2R	7998313	C-A	0.12		
aura T5	2R	24190132	G-T	0.12	*ocm*	FBgn0266083
lgl_T10	2R	7765531	G-T	0.13	*CG45093*	FBgn0266526
mbtL1 T5	2R	11709329	G-T	0.10		
mbtL1 T5	2R	8925785	G-T	0.13	*CG8237*	FBgn0033350
lgl_T5	2R	7358796	G-T	0.11	*Dscam1*	FBgn0033159
aura T5	2R	20416103	C-A	0.14		
lgl_T5	2R	23156687	G-T	0.17	*CG9899*	FBgn0034829
aura T0	2R	16315347	G-T	0.12	*Nox*	FBgn0085428
lgl_T10	2R	24580968	G-T	0.16		
mbtL2 T5	2R	10186546	G-T	0.10	*KCNQ*	FBgn0033494
mbtL1 T5	2R	13617644	G-C	0.61		
lgl_T5	2R	9391701	C-A	0.12	*l(2)03659*	FBgn0010549
aura T5	2R	7343661	G-T	0.18	*Dscam1*	FBgn0033159
lgl_T10	2R	20143261	G-T	0.19		
mbtL2 T5	2R	19965398	G-T	0.11		
lgl_T10	2R	7456214	G-T	0.11	*Inos*	FBgn0025885
lgl_T5	2R	10576912	A-T	0.21	*psq*	FBgn0263102
mbtL2 T5	2R	6802263	C-A	0.11		
aura T5	2R	15366754	G-T	0.10	*unc-5*	FBgn0034013
lgl_T10	2R	21222142	C-A	0.10	*ASPP*	FBgn0034606
mbtL2 T10A	2R	13024473	T-A	0.20		
lgl_T5	2R	5356064	G-T	0.13		
aura T5	2R	23685068	C-A	0.19	*l(2)efl*	FBgn0011296
mbtL2 T5	2R	10428530	C-T	0.55		
lgl_T10	2R	12158833	G-T	0.09	*Prp8*	FBgn0033688
lgl_T10	2R	11055999	G-T	0.13	*luna*	FBgn0040765
aura T5	2R	18721634	G-T	0.11	*Hs3st-A*	FBgn0053147
lgl_T10	2R	22485772	G-T	0.16	*CG4554*	FBgn0034734
mbtL2 T10B	2R	6779172	C-A	0.11	*Epac*	FBgn0085421
lgl_T10	2R	13776206	G-T	0.13	*Cpr50Ca*	FBgn0033867
lgl_T10	2R	24828605	G-T	0.13	*Dll*	FBgn0000157
aura T5	2R	12943070	C-A	0.12	*Mdr49*	FBgn0004512
aura T5	2R	20632282	G-T	0.11	*Rcd6*	FBgn0034530
lgl_T10	2R	18159837	C-A	0.14	*CG10914*	FBgn0034307
mbtL2 T5	2R	19656767	C-A	0.09	*CR44214*	FBgn0265145
mbtL2 T5	2R	11214384	C-A	0.17	*shn*	FBgn0003396
lgl_T10	2R	17024559	G-T	0.10	*CR44390*	FBgn0265540
mbtL2 T10A	2R	3622914	A-C	0.25		
mbtL2 T10B	2R	3622914	A-C	0.31		
lgl_T5	2R	12802455	G-T	0.13		
lgl_T5	2R	23488988	G-T	0.12	*CG43795*	FBgn0264339
lgl_T10	2R	21419919	G-T	0.19	*Sdc*	FBgn0010415
lgl_T10	2R	13170553	C-A	0.17	*Spt-I*	FBgn0086532
mbtL2 T5	2R	20162174	G-T	0.09		
lgl_T5	2R	8770158	C-A	0.17		
aura T5	2R	15950817	C-A	0.08	*Strn-Mlck*	FBgn0265045
lgl_T10	2R	13364562	G-T	0.10		
aura T5	2R	9185465	G-T	0.12	*ltd*	FBgn0002567
lgl_T10	2R	24292291	G-T	0.10		
lgl_T10	2R	16182123	C-A	0.11	*Lis-1*	FBgn0015754
lgl_T10	2R	13550417	G-T	0.15	*CG33156*	FBgn0053156
mbtL2 T5	2R	6984275	C-A	0.11	*CG30156*	FBgn0050156
mbtL2 T10B	2R	15930789	G-T	0.13	*bdg*	FBgn0034049
lgl_T5	2R	9572288	G-T	0.12	*Not1*	FBgn0085436
lgl_T10	2R	25007116	G-T	0.11	*zip*	FBgn0265434

lgl_T5	2R	12979992	G-T	0.12	*Psc*	FBgn0005624
lgl_T5	2R	22732258	G-T	0.14	*jbug*	FBgn0028371
mbtL1 T5	2R	11401177	C-A	0.17		
lgl_T5	2R	21495186	G-T	0.13	*Fkbp14*	FBgn0010470
lgl_T10	2R	15995810	C-A	0.11	*ATPCL*	FBgn0020236
mbtL2 T5	2R	11002247	C-G	0.17	*luna*	FBgn0040765
aura T5	2R	23443071	G-T	0.14		
aura T5	2R	13106231	G-T	0.14		
aura T5	2R	18249352	C-A	0.16		
mbtL1 T5	2R	16835592	C-A	0.13	*CR43650*	FBgn0263659
lgl_T10	2R	9696729	G-T	0.12		
mbtL1 T5	2R	6661735	C-A	0.10	*Dpit47*	FBgn0266518
lgl_T5	2R	4297359	C-A	0.08		
lgl_T5	2R	24956287	G-T	0.17	*CR44830*	FBgn0266088
lgl_T10	2R	7194083	C-A	0.11	*pk*	FBgn0003090
aura T5	2R	18392277	T-G	0.33		
lgl_T5	2R	24753467	C-A	0.11	*CG30421*	FBgn0050421
lgl_T5	2R	17090770	G-T	0.13	*Sply*	FBgn0010591
lgl_T10	2R	23176020	G-T	0.20	*CG34371*	FBgn0085400
aura T5	2R	7635459	C-A	0.14	*CG43340*	FBgn0263077
mbtL2 T10B	2R	6553006	G-A	0.47	*jing*	FBgn0086655
aura T5	2R	22116095	G-T	0.13	*Vrp1*	FBgn0243516
lgl_T5	2R	22866374	C-A	0.09		
mbtL1 T5	2R	14244119	C-A	0.12	*CG8503*	FBgn0033917
lgl_T10	2R	24103932	C-A	0.11	*enok*	FBgn0034975
lgl_T10	2R	7012519	G-T	0.14	*Tsp42Ee*	FBgn0029506
lgl_T5	2R	25088342	C-A	0.16	*lov*	FBgn0266129
aura T5	2R	24393158	G-T	0.08	*slik*	FBgn0035001
lgl_T5	2R	13110074	G-T	0.13		
aura T5	2R	6934157	G-T	0.10	*mim*	FBgn0053558
lgl_T5	2R	23285526	C-A	0.12	*CG13541*	FBgn0034841
lgl_T5	2R	7826509	G-T	0.07	*phr*	FBgn0003082
lgl_T5	2R	11809194	C-A	0.10	*ths*	FBgn0033652
aura T5	2R	23880602	G-T	0.12	*TM4SF*	FBgn0020372
mbtL1 T5	2R	9386798	G-T	0.11	*CG13954*	FBgn0033405
aura T5	2R	10349188	C-A	0.13	*CG42732*	FBgn0261698
lgl_T5	2R	12434390	G-T	0.11	*DUBAI*	FBgn0033738
lgl_T5	2R	14329740	C-A	0.16	*Shroom*	FBgn0085408
lgl_T5	2R	24613755	G-T	0.14	*CG3608*	FBgn0035039
lgl_T5	2R	12574261	C-A	0.12	*ClC-b*	FBgn0033755
mbtL1 T5	2R	24541085	G-T	0.12	*ITP*	FBgn0035023
aura T5	2R	13221818	G-T	0.12	*Fsn*	FBgn0043010
lgl_T5	2R	24424783	C-A	0.14	*CG42383*	FBgn0259729
aura T5	2R	7073650	C-A	0.13	*CG12831*	FBgn0033141
lgl_T10	2R	9542728	G-T	0.13	*CG1888*	FBgn0033421
aura T5	2R	10141310	G-T	0.10	*Or46a*	FBgn0026388
aura T5	2R	15756038	G-T	0.12	*CG30089*	FBgn0050089
aura T5	2R	3502485	C-T	0.29		
aura T5	2R	13326496	C-A	0.11		
lgl_T10	2R	22628574	G-T	0.13	*CG33143*	FBgn0053143
mbtL2 T5	2R	17660448	G-T	0.27	*CCHa1-R*	FBgn0050106
mbtL1 T5	2R	16969955	G-T	0.11	*CR44377*	FBgn0265527
aura T5	2R	24493646	C-A	0.11	*CG13590*	FBgn0035012
aura T0	2R	23477427	C-A	0.19	*CR43793*	FBgn0264337
lgl_T10	2R	23970892	C-A	0.14	*CG3065*	FBgn0034946
mbtL2 T10A	2R	544601	G-T	0.49	*CG45781*	FBgn0267428
mbtL2 T10B	2R	544601	G-T	0.56	*CG45781*	FBgn0267428
lgl_T5	2R	17676496	C-A	0.11	*Sema-1b*	FBgn0016059
aura T5	2R	21607580	G-T	0.09	*CG10433*	FBgn0034638
lgl_T5	2R	22556114	C-A	0.12	*px*	FBgn0003175
lgl_T5	2R	22025615	G-T	0.11	*CG34370*	FBgn0085399
lgl_T5	2R	16028448	C-A	0.19		
lgl_T10	2R	8176073	G-A	0.11	*LRP1*	FBgn0053087

mbtL2 T10A	2R	19637875	C-A	0.12	*CG43277*	FBgn0262966
aura T5	2R	18485833	G-T	0.11	*CG30116*	FBgn0028496
aura T5	2R	7500791	G-T	0.12	*didum*	FBgn0261397
lgl_T5	2R	5899678	T-G	0.43	*CG43366*	FBgn0263109
aura T5	2R	10231078	G-T	0.13	*CG12912*	FBgn0033497
aura T5	2R	15646747	G-T	0.11	*CG42524*	FBgn0260429
lgl_T5	2R	17762844	G-T	0.10	*CG5009*	FBgn0027572
lgl_T5	2R	7619947	G-T	0.09	*CG1358*	FBgn0033196
lgl_T5	2R	21929892	C-A	0.11	*Fili*	FBgn0085397
aura T5	2R	15557521	C-A	0.14	*CG12963*	FBgn0034031
lgl_T5	2R	7439685	C-A	0.10		
mbtL2 T10A	2R	4288064	C-A	0.24		
lgl_T5	2R	7698047	G-T	0.19	*CG1399*	FBgn0033212
lgl_T10	2R	18237713	C-A	0.11		
aura T5	2R	10031713	G-T	0.09	*CG1407*	FBgn0033474
aura T5	2R	23761725	C-A	0.14	*CG9850*	FBgn0034903
lgl_T10	2R	17791040	C-A	0.12	*CG5033*	FBgn0028744
lgl_T5	2R	24497264	C-A	0.14		
lgl_T10	2R	23038292	G-T	0.10	*CG42741*	FBgn0261705
aura T5	2R	24261305	G-T	0.27	*CG3376*	FBgn0034997
aura T5	2R	18091793	G-T	0.16	*CG34386*	FBgn0085415
lgl_T0	2R	6505350	C-A	0.16	*jing*	FBgn0086655
lgl_T10	2R	6504961	C-A	0.17	*jing*	FBgn0086655
lgl_T10	2R	14681033	G-T	0.12	*mspo*	FBgn0020269
lgl_T5	2R	14808767	G-T	0.10	*hui*	FBgn0033968
aura T5	2R	7407455	G-T	0.11	*Eaf*	FBgn0033166
lgl_T10	2R	23239814	C-A	0.14		
lgl_T10	2R	25094715	G-T	0.12	*lov*	FBgn0266129
lgl_T10	2R	17899044	C-A	0.10	*Elk*	FBgn0011589
lgl_T5	2R	10321355	C-A	0.11	*Ndg*	FBgn0026403
lgl_T10	2R	22178967	G-T	0.13	*CG11170*	FBgn0034705
lgl_T5	2R	11923337	C-A	0.14		
aura T5	2R	15423909	C-A	0.10	*CG30472*	FBgn0050472
lgl_T5	2R	11865598	G-A	0.18	*Damm*	FBgn0033659
aura T5	2R	18023169	G-T	0.13		
lgl_T5	2R	22459593	G-T	0.15	*CG10384*	FBgn0034731
lgl_T5	2R	10656326	G-T	0.22		
aura T5	2R	8436367	C-A	0.63		
lgl_T10	2R	17717911	G-T	0.12	*CG6424*	FBgn0028494
mbtL1 T5	2R	17503221	A-C	0.47	*CG43164*	FBgn0262720
lgl_T5	2R	17814114	G-T	0.17	*grh*	FBgn0259211
lgl_T5	2R	18307456	C-A	0.10	*sbb*	FBgn0010575
lgl_T5	2R	24812402	G-T	0.09		
aura T5	2R	8047912	C-A	0.12		
lgl_T10	2R	18283818	G-T	0.10	*sbb*	FBgn0010575
lgl_T10	2R	12641146	C-A	0.13	*CG8785*	FBgn0033760
lgl_T10	2R	15416245	G-T	0.13	*CG43729*	FBgn0263980
lgl_T10	2R	17835869	C-A	0.16	*grh*	FBgn0259211
lgl_T10	2R	17943791	G-T	0.14		
aura T5	2R	7686115	C-A	0.12	*CG1399*	FBgn0033212
lgl_T5	2R	8208843	C-A	0.10	*LRP1*	FBgn0053087
lgl_T10	2R	6880172	G-T	0.10	*Spn42Db*	FBgn0033112
aura T5	2R	15474249	C-A	0.09	*CG8180*	FBgn0034021
mbtL2 T5	2R	19698347	G-T	0.12	*Obp56a*	FBgn0034468
lgl_T10	2R	22220345	C-A	0.07	*Oatp58Da*	FBgn0050277
aura T5	2R	8476637	G-T	0.09	*mtt*	FBgn0050361
aura T5	2R	6804801	C-A	0.11		
lgl_T10	2R	18323162	G-T	0.12	*sbb*	FBgn0010575
lgl_T10	2R	3524634	C-A	0.12	*CR41501*	FBgn0085786
lgl_T10	2R	13816835	G-T	0.08	*stj*	FBgn0261041
lgl_T10	2R	7050456	C-A	0.09	*lbm*	FBgn0016032
lgl_T10	2R	20973005	C-A	0.15	*CG15649*	FBgn0034563
lgl_T10	2R	9020472	G-T	0.11	*CG8170*	FBgn0033365

lgl_T5	2R	7487389	C-A	0.10	*wech*	FBgn0259745
aura T5	2R	8512368	C-A	0.11	*mtt*	FBgn0050361
lgl_T5	2R	7522415	C-A	0.12	*CG2144*	FBgn0033187
lgl_T10	2R	22253063	C-A	0.13	*dve*	FBgn0020307
mbtL1 T5	2R	16866542	G-T	0.16	*Psi*	FBgn0014870
lgl_T5	2R	23518119	C-A	0.12		
lgl_T5	2R	24311053	G-T	0.11	*betaTub60D*	FBgn0003888
lgl_T5	2R	8236508	C-A	0.11		
aura T5	2R	6961557	C-A	0.12	*mim*	FBgn0053558
lgl_T5	2R	17840498	G-T	0.10	*grh*	FBgn0259211
lgl_T10	2R	9046590	C-A	0.08	*CG13743*	FBgn0033368
lgl_T10	2R	24606467	G-T	0.16	*CG4707*	FBgn0035036
lgl_T10	2R	25031224	C-A	0.15	*uzip*	FBgn0004055
lgl_T10	2R	22509706	G-T	0.12	*px*	FBgn0003175
lgl_T5	2R	12458094	C-A	0.12	*Lac*	FBgn0010238
lgl_T5	2R	12597810	G-T	0.14	*Amph*	FBgn0027356
lgl_T10	2R	12181459	C-A	0.20	*wash*	FBgn0033692
lgl_T5	2R	23952470	C-A	0.11		
lgl_T5	2R	15932726	G-T	0.11	*Diap2*	FBgn0015247
lgl_T5	2R	10595627	C-A	1.00	*psq*	FBgn0263102
lgl_T10	2R	16200070	C-A	0.10	*shark*	FBgn0015295
lgl_T10	2R	22644131	G-T	0.07		
lgl_T10	2R	22524633	G-T	0.11	*px*	FBgn0003175
lgl_T5	2R	7535796	C-A	0.10	*Cyt-b5*	FBgn0264294
lgl_T5	2R	7450997	G-T	0.11	*fa2h*	FBgn0050502
lgl_T5	2R	14819442	G-T	0.15	*CG10202*	FBgn0033969
aura T5	2R	21617901	G-T	0.14	*clt*	FBgn0000326
aura T5	2R	3393583	T-A	0.23		
aura T5	2R	15117609	G-T	0.08	*chn*	FBgn0015371
lgl_T10	2R	20551508	G-T	0.13	*CG18065*	FBgn0034519
lgl_T5	2R	24762250	C-A	0.12	*CG3880*	FBgn0035057
aura T5	2R	15432105	C-A	0.11	*CG43729*	FBgn0263980
lgl_T5	2R	20996955	C-A	0.11	*CG10543*	FBgn0034570
mbtL1 T0	2R	15234604	G-T	0.15	*Fs*	FBgn0259878
mbtL1 T5	2R	14249846	G-T	0.12	*Mdr50*	FBgn0010241
lgl_T10	2R	19449249	G-T	0.10	*betaTub56D*	FBgn0003887
lgl_T5	2R	10600846	C-A	0.10	*psq*	FBgn0263102
mbtL2 T10A	2R	11002247	C-G	0.53	*luna*	FBgn0040765
aura T5	2R	9954691	G-T	0.25	*Mef2*	FBgn0011656
aura T5	2R	7641748	C-A	0.11	*CG43340*	FBgn0263077
aura T5	2R	7638062	G-T	0.15	*CG43340*	FBgn0263077
lgl_T10	2R	13778413	G-T	0.14	*Cpr50Ca*	FBgn0033867
lgl_T5	2R	21326508	C-A	0.16	*dom*	FBgn0020306
mbtL1 T5	2R	9387691	G-A	0.18	*CG13954*	FBgn0033405
lgl_T10	2R	7456840	G-T	0.10	*Inos*	FBgn0025885
lgl_T5	2R	22460214	G-T	0.10	*CG10384*	FBgn0034731
lgl_T10	2R	3052321	G-T	0.11		
mbtL1 T5	2R	3840283	C-T	0.10		
bratL2 T5	2R	4730574	T-G	0.10	*CR40282*	FBgn0039979
bratL2 T0	2R	3133813	A-C	0.23	*CG42596*	FBgn0260995
mbtL2 T5	2R	23750625	A-G	0.12		
bratL2 T5	2R	4730585	A-G	0.11	*CR40282*	FBgn0039979
bratL1 T10	2R	5151005	C-G	0.59	*Atf6*	FBgn0033010
mbtL2 T10A	2R	445494	G-C	0.59	*CG45781*	FBgn0267428
mbtL2 T10A	2R	1845585	G-C	0.31		
mbtL2 T10B	2R	445494	G-C	0.63	*CG45781*	FBgn0267428
mbtL2 T10B	2R	1845585	G-C	0.29		
aura T0	2R	488366	A-G	0.11	*CG45781*	FBgn0267428
mbtL2 T5	2R	22361832	A-G	0.10	*Liprin-gamma*	FBgn0034720
bratL1 T10	2R	7744260	T-C	0.61	*CG45093*	FBgn0266526
lgl_T10	2R	3052322	C-G	0.10		
mbtL2 T10A	2R	4207376	G-A	0.17		
bratL1 T5	2R	3678239	A-T	0.32		

lgl_T0	2R	6437682	C-T	0.26		
mbtL2 T5	2R	3622914	A-C	0.29		
mbtL1 T10	2R	588063	G-T	0.11	*CG45781*	FBgn0267428
bratL1 T0	2R	3678239	A-T	0.25		
bratL2 T5	2R	3133813	A-C	0.17	*CG42596*	FBgn0260995
mbtL1 T5	2R	2530844	G-T	0.12	*MFS17*	FBgn0058263
aura T5	2R	1379562	C-A	0.10	*l(2)41Ab*	FBgn0262123
bratL1 T10	2R	1326929	T-C	0.17		
mbtL1 T0	2R	1385676	T-A	0.24	*l(2)41Ab*	FBgn0262123
lgl_T10	2R	80978	C-A	0.10		
lgl_T5	2R	1149961	C-A	0.13		
bratL2 T0	2R	488363	C-A	0.16	*CG45781*	FBgn0267428
mbtL2 T10B	2R	80826	G-C	0.46		
mbtL2 T10A	2R	80826	G-C	0.44		
mbtL2 T0	2R	6747	T-A	0.08		
aura T0	2R	488363	C-A	0.11	*CG45781*	FBgn0267428
bratL2 T0	3L	27172809	C-T	0.14		
mbtL2 T0	3L	21834250	A-G	0.24	*Nopp140*	FBgn0037137
mbtL2 T10A	3L	13087529	G-T	0.14	*CR45749*	FBgn0267313
bratL1 T5	3L	24534569	T-A	0.13	*CR12460*	FBgn0040045
bratL1 T0	3L	24308588	T-C	0.13		
mbtL2 T10B	3L	11386254	G-T	0.13		
bratL1 T10	3L	22198469	A-T	0.69	*olf413*	FBgn0037153
aura T0	3L	21732933	C-A	0.15	*CG14568*	FBgn0037124
bratL1 T0	3L	13782336	A-G	0.24	*bru-3*	FBgn0264001
mbtL2 T10B	3L	20136373	G-T	0.11	*gig*	FBgn0005198
aura T0	3L	11470315	G-T	0.40		
bratL2 T5	3L	16834965	G-T	0.24		
lgl_T0	3L	15673606	G-T	0.11		
mbtL1 T10	3L	9657008	C-G	0.09	*CNMaR*	FBgn0053696
bratL1 T5	3L	9872577	C-A	0.24		
mbtL2 T10A	3L	24534113	T-C	0.13	*CR12460*	FBgn0040045
mbtL1 T10	3L	13868380	G-T	0.14	*dysc*	FBgn0264006
mbtL1 T0	3L	20856971	T-G	0.86	*CG32432*	FBgn0052432
mbtL1 T0	3L	13639332	C-A	0.17	*bru-3*	FBgn0264001
mbtL1 T10	3L	22043812	C-T	0.56	*Oct-TyrR*	FBgn0004514
mbtL2 T5	3L	12766081	C-A	0.14	*CG32113*	FBgn0052113
aura T0	3L	25496611	A-T	0.14	*CG45782*	FBgn0267429
lgl_T0	3L	22380474	G-T	0.13	*CR45962*	FBgn0267624
lgl_T0	3L	18728988	C-A	0.15		
mbtL2 T0	3L	4851927	A-C	0.17	*CG32237*	FBgn0052237
mbtL2 T10A	3L	20212194	A-C	0.57		
mbtL2 T10A	3L	17550319	G-T	0.11	*CG7542*	FBgn0036738
mbtL2 T5	3L	18292561	G-T	0.10	*CG5103*	FBgn0036784
bratL1 T0	3L	6161759	A-C	0.57		
mbtL2 T10B	3L	13855171	C-A	0.18	*Hml*	FBgn0029167
mbtL2 T0	3L	8262780	G-T	0.24	*Dscam4*	FBgn0263219
mbtL1 T5	3L	17501244	C-A	0.38	*Oatp74D*	FBgn0036732
mbtL2 T5	3L	4645265	G-T	0.14	*axo*	FBgn0262870
mbtL1 T0	3L	16754622	C-A	0.12		
mbtL2 T5	3L	20623593	C-A	0.21		
mbtL2 T0	3L	24086673	A-G	0.17	*Snap25*	FBgn0011288
bratL1 T5	3L	13789668	C-A	0.30	*bru-3*	FBgn0264001
mbtL1 T10	3L	25190093	C-T	0.18		
aura T5	3L	25079806	C-A	0.11		
bratL1 T10	3L	24308588	T-C	0.23		
mbtL1 T10	3L	17243585	G-T	0.30	*CG7707*	FBgn0036703
bratL2 T5	3L	18864947	A-G	0.57	*CR45912*	FBgn0267572
aura T0	3L	13456748	C-A	0.15	*CG10116*	FBgn0036367
mbtL2 T10A	3L	15041894	C-A	0.22	*Sytbeta*	FBgn0261090
mbtL2 T5	3L	22432918	G-T	0.10		
mbtL1 T0	3L	24808494	A-G	0.14		
bratL1 T5	3L	11616660	C-T	0.09	*Muc68Ca*	FBgn0036181

mbtL2 T5	3L	8317050	C-A	0.10		
mbtL1 T10	3L	4236483	G-T	0.13		
lgl_T10	3L	24308588	T-C	0.09		
lgl_T0	3L	10811129	G-T	0.14		
mbtL1 T5	3L	20630137	G-T	0.12		
mbtL1 T5	3L	6201275	G-T	0.14	*CG8219*	FBgn0035693
mbtL1 T5	3L	11853434	G-T	0.14	*CG44837*	FBgn0266100
mbtL1 T5	3L	24515326	T-C	0.44		
mbtL1 T5	3L	8540552	C-A	0.11		
lgl_T0	3L	24308588	T-C	0.18		
aura T5	3L	26463406	T-C	0.25		
lgl_T5	3L	27132949	G-T	0.10		
mbtL2 T5	3L	24534130	T-A	0.15	*CR12460*	FBgn0040045
mbtL2 T10B	3L	24534113	T-C	0.18	*CR12460*	FBgn0040045
mbtL2 T5	3L	2297535	G-T	0.12	*MsR2*	FBgn0264002
mbtL2 T10B	3L	21523977	C-A	0.13	*CG7611*	FBgn0037094
mbtL2 T5	3L	15789006	C-A	0.14		
aura T5	3L	21413762	C-A	0.12	*rgn*	FBgn0261258
lgl_T10	3L	17635215	C-A	0.14	*CG7497*	FBgn0036742
mbtL1 T0	3L	22043812	C-T	0.62	*Oct-TyrR*	FBgn0004514
mbtL1 T10	3L	27172800	T-G	0.16		
mbtL2 T10B	3L	26629884	G-C	0.21		
lgl_T10	3L	5367105	G-T	0.12	*Membrin*	FBgn0260856
lgl_T5	3L	24308588	T-C	0.18		
aura T5	3L	7415514	C-A	0.17		
lgl_T5	3L	23183962	C-A	0.14	*CG34031*	FBgn0054031
bratL1 T0	3L	25969348	C-A	0.13	*CG40178*	FBgn0058178
lgl_T5	3L	13445870	C-A	0.14	*CG10732*	FBgn0036365
mbtL1 T10	3L	2027204	G-T	0.12	*CG34259*	FBgn0085288
mbtL1 T0	3L	23064343	C-A	0.29		
mbtL1 T5	3L	2694304	C-A	0.10	*Fife*	FBgn0264606
mbtL1 T5	3L	4671982	G-T	0.10	*axo*	FBgn0262870
mbtL1 T5	3L	1678010	C-A	0.11	*CG7991*	FBgn0035260
mbtL2 T0	3L	5819269	C-A	0.22	*vn*	FBgn0003984
aura T0	3L	26463406	T-C	0.27		
lgl_T10	3L	9969238	C-A	0.16	*Dronc*	FBgn0026404
lgl_T10	3L	14414865	G-T	0.13	*bbg*	FBgn0087007
mbtL2 T10B	3L	22509911	C-A	0.14		
mbtL2 T0	3L	27452275	G-T	0.21		
lgl_T5	3L	15357016	T-G	0.14		
mbtL1 T10	3L	23064366	T-C	0.29		
mbtL2 T5	3L	14321964	G-T	0.11	*fz*	FBgn0001085
mbtL1 T5	3L	7100402	G-T	0.25	*form3*	FBgn0053556
mbtL2 T0	3L	26509241	A-T	0.19		
lgl_T5	3L	5781535	C-A	0.10	*Pmi*	FBgn0044419
mbtL1 T5	3L	3660180	G-T	0.10		
aura T5	3L	12286409	C-A	0.20		
lgl_T0	3L	25190093	C-T	0.18		
aura T5	3L	10728628	G-T	0.21		
aura T5	3L	4250611	C-A	0.10	*ago*	FBgn0041171
mbtL1 T10	3L	5068115	C-A	0.08	*Con*	FBgn0005775
mbtL1 T10	3L	15173861	G-T	0.10		
lgl_T10	3L	21985777	C-A	0.11	*Act79B*	FBgn0000045
aura T5	3L	13483046	C-A	0.12	*stv*	FBgn0086708
mbtL1 T0	3L	14433817	G-T	0.12	*bbg*	FBgn0087007
mbtL1 T10	3L	18267612	C-A	0.16		
aura T5	3L	17109909	G-T	0.15	*Rbp6*	FBgn0260943
lgl_T5	3L	25073743	C-T	0.11		
mbtL2 T10B	3L	27387272	C-T	0.24	*Dbp80*	FBgn0024804
aura T5	3L	14438427	C-A	0.25	*bbg*	FBgn0087007
aura T5	3L	16342589	C-A	0.12	*CG4998*	FBgn0036612
mbtL2 T5	3L	23183962	C-A	0.16	*CG34031*	FBgn0054031
mbtL2 T10A	3L	25278315	A-G	0.86	*CG45782*	FBgn0267429

mbtL2 T10B	3L	25278315	A-G	0.63	*CG45782*	FBgn0267429
lgl_T5	3L	9213190	C-A	0.12	*CG4476*	FBgn0035969
lgl_T5	3L	7044952	G-T	0.11	*Mp*	FBgn0260660
aura T5	3L	5212127	G-T	0.15	*shep*	FBgn0052423
mbtL1 T0	3L	25503394	A-C	0.18	*CG45782*	FBgn0267429
mbtL2 T5	3L	25503321	G-A	0.22	*CG45782*	FBgn0267429
aura T5	3L	2547570	G-T	0.10	*Spn*	FBgn0010905
mbtL1 T5	3L	15036952	G-T	0.11	*Sytbeta*	FBgn0261090
mbtL1 T5	3L	25190093	C-T	0.17		
mbtL2 T10B	3L	23183962	C-A	0.15	*CG34031*	FBgn0054031
aura T5	3L	11400908	G-T	0.17		
aura T5	3L	18678665	C-A	0.09		
lgl_T10	3L	25190002	G-A	0.15		
mbtL2 T0	3L	25190002	G-A	0.22		
aura T5	3L	18008117	C-A	0.16	*Eip75B*	FBgn0000568
mbtL2 T5	3L	13408794	C-A	0.13		
mbtL2 T10B	3L	14490055	G-C	0.56	*bbg*	FBgn0087007
mbtL1 T5	3L	9327679	G-T	0.07		
lgl_T5	3L	18091174	C-A	0.14	*CG13698*	FBgn0036773
lgl_T10	3L	20876811	G-T	0.21	*CG32432*	FBgn0052432
lgl_T5	3L	18705618	G-T	0.13		
bratL1 T0	3L	24916749	T-A	0.24		
mbtL2 T5	3L	5778035	C-A	0.09	*Myt1*	FBgn0040298
mbtL2 T5	3L	9049943	G-T	0.08	*Argk*	FBgn0000116
aura T5	3L	22008526	C-A	0.13		
lgl_T10	3L	13178860	G-T	0.15		
lgl_T5	3L	17193549	G-T	0.12	*Rbp6*	FBgn0260943
lgl_T5	3L	2142515	G-T	0.27	*zormin*	FBgn0052311
lgl_T5	3L	681300	G-T	0.15	*CG3386*	FBgn0035152
lgl_T5	3L	12407657	G-T	0.13	*CG32103*	FBgn0052103
lgl_T10	3L	10543714	G-C	0.39	*A2bp1*	FBgn0052062
mbtL1 T10	3L	2564802	G-T	0.09	*msn*	FBgn0010909
mbtL2 T5	3L	5174166	C-A	0.13	*shep*	FBgn0052423
mbtL1 T5	3L	10330876	C-A	0.11	*CR46006*	FBgn0267668
lgl_T10	3L	6184193	C-A	0.11	*D19B*	FBgn0022699
aura T5	3L	15591154	G-T	0.17	*RhoGAP71E*	FBgn0036518
mbtL1 T10	3L	26027114	G-T	0.13		
mbtL1 T5	3L	13018573	C-A	0.15	*ste14*	FBgn0036336
mbtL1 T5	3L	12368263	C-A	0.24		
lgl_T0	3L	22895141	G-T	0.11		
lgl_T5	3L	7621163	C-A	0.13	*CG33275*	FBgn0035802
lgl_T5	3L	20578241	C-A	0.17		
lgl_T10	3L	9005312	C-A	0.17	*Doc3*	FBgn0035954
mbtL1 T5	3L	21732933	C-A	0.24	*CG14568*	FBgn0037124
lgl_T5	3L	21070399	G-T	0.12	*siz*	FBgn0026179
lgl_T5	3L	11848820	G-T	0.15		
mbtL1 T5	3L	17992917	C-A	0.13	*Eip75B*	FBgn0000568
mbtL1 T5	3L	9804899	G-T	0.14	*CG32052*	FBgn0044328
lgl_T5	3L	4209420	C-A	0.15	*CG43367*	FBgn0263110
lgl_T10	3L	6653107	C-A	0.09		
mbtL1 T10	3L	14347468	G-C	0.41	*fz*	FBgn0001085
lgl_T10	3L	8191460	G-T	0.15	*MED24*	FBgn0035851
mbtL2 T0	3L	8723292	T-G	0.10	*CG6511*	FBgn0035923
lgl_T10	3L	18385601	C-A	0.11		
lgl_T10	3L	15238490	C-A	0.10	*Tollo*	FBgn0029114
lgl_T10	3L	19812513	C-A	0.12	*Cyp305a1*	FBgn0036910
lgl_T10	3L	2977144	G-T	0.14		
mbtL1 T5	3L	18432676	G-T	0.11		
aura T5	3L	899352	G-T	0.14		
lgl_T10	3L	7089111	C-A	0.14		
aura T5	3L	22960533	C-A	0.16		
aura T5	3L	14857111	G-T	0.16	*CG17839*	FBgn0036454
mbtL1 T5	3L	26027143	C-T	0.15		

lgl_T10	3L	1613378	C-A	0.19	*CG12105*	FBgn0035241
lgl_T5	3L	16604301	G-T	0.11	*CG32164*	FBgn0042177
aura T5	3L	2950449	C-A	0.16	*Shab*	FBgn0262593
aura T5	3L	9463953	C-A	0.11	*CG44838*	FBgn0266101
lgl_T10	3L	22635435	G-T	0.11	*CG14459*	FBgn0037171
lgl_T5	3L	10850548	G-T	0.10	*CR45169*	FBgn0266679
mbtL1 T5	3L	22568458	C-A	0.13		
mbtL1 T5	3L	21213084	C-A	0.20	*CG10508*	FBgn0037060
aura T5	3L	8886734	C-A	0.20	*dally*	FBgn0263930
lgl_T10	3L	14783708	C-A	0.11	*bmm*	FBgn0036449
lgl_T5	3L	2510541	G-T	0.15	*Spn*	FBgn0010905
lgl_T10	3L	16050439	C-T	1.00	*Diap1*	FBgn0260635
aura T5	3L	19616929	C-A	0.10	*l(3)76BDm*	FBgn0260655
lgl_T5	3L	25760372	C-A	0.25	*CG17374*	FBgn0040001
aura T5	3L	9823679	C-A	0.12	*CG43897*	FBgn0264489
mbtL2 T5	3L	6134868	C-A	0.29	*Lcp65Ag1*	FBgn0020638
lgl_T5	3L	19600788	G-T	0.09	*Taf6*	FBgn0010417
lgl_T5	3L	6328314	C-A	0.14	*Or65c*	FBgn0041623
lgl_T5	3L	21695649	G-T	0.13	*CG32447*	FBgn0052447
aura T5	3L	5937809	C-A	0.13	*CG33993*	FBgn0053993
lgl_T5	3L	9628227	G-T	0.16	*Or67b*	FBgn0036019
lgl_T5	3L	1572808	C-A	0.11	*CG12004*	FBgn0035236
lgl_T10	3L	10881149	G-T	0.11	*CG6409*	FBgn0036106
lgl_T10	3L	1141525	G-T	0.11	*bab2*	FBgn0025525
lgl_T10	3L	2533094	C-A	0.13	*Spn*	FBgn0010905
mbtL1 T5	3L	19076990	G-T	0.12	*Mkp3*	FBgn0036844
lgl_T10	3L	15571616	C-A	0.12	*dop*	FBgn0267390
mbtL1 T5	3L	13342425	G-T	0.09		
mbtL1 T5	3L	13872582	G-T	0.12	*dysc*	FBgn0264006
mbtL2 T10A	3L	25825340	A-C	0.12	*CG40178*	FBgn0058178
lgl_T10	3L	3667084	C-A	0.11		
mbtL1 T5	3L	22886283	C-A	0.11	*slif*	FBgn0037203
aura T5	3L	7859864	G-T	0.12	*Pdp1*	FBgn0016694
lgl_T10	3L	25503394	A-C	0.20	*CG45782*	FBgn0267429
mbtL1 T10	3L	25503394	A-C	0.24	*CG45782*	FBgn0267429
mbtL1 T5	3L	25503394	A-C	0.28	*CG45782*	FBgn0267429
lgl_T0	3L	25503394	A-C	0.24	*CG45782*	FBgn0267429
mbtL1 T5	3L	22043812	C-T	0.57	*Oct-TyrR*	FBgn0004514
mbtL1 T5	3L	18743292	G-T	0.21		
lgl_T5	3L	2901304	C-A	0.15	*Shab*	FBgn0262593
aura T5	3L	20172188	C-A	0.13	*CG42674*	FBgn0261556
lgl_T10	3L	12358152	C-A	0.11		
lgl_T5	3L	13985709	C-A	0.12	*CG17364*	FBgn0036391
aura T5	3L	8160739	G-T	0.10	*CG13675*	FBgn0035845
lgl_T5	3L	10238940	G-T	0.13		
lgl_T10	3L	18685068	C-A	0.11	*Cyp12c1*	FBgn0036806
lgl_T5	3L	9927385	G-T	0.10	*CG34356*	FBgn0085385
lgl_T5	3L	18999174	G-T	0.17	*CG3808*	FBgn0036838
lgl_T10	3L	17927007	G-T	0.14		
lgl_T10	3L	5648156	C-A	0.10	*Blimp-1*	FBgn0035625
lgl_T5	3L	11131287	C-A	0.14	*nol*	FBgn0014368
lgl_T5	3L	4747369	C-A	0.15	*RhoGEF64C*	FBgn0035574
mbtL2 T5	3L	24808494	A-G	0.08		
lgl_T5	3L	8346828	C-A	0.21	*GAPcenA*	FBgn0035879
lgl_T5	3L	20070338	C-A	0.16	*sNPF-R*	FBgn0036934
lgl_T5	3L	8073607	C-A	0.10	*Ect4*	FBgn0262579
mbtL1 T5	3L	14347468	G-C	0.16	*fz*	FBgn0001085
aura T5	3L	4513984	G-T	0.09	*Tie*	FBgn0014073
lgl_T10	3L	11311779	T-C	0.25	*CG7573*	FBgn0036153
lgl_T5	3L	3481780	G-T	0.20	*CG42324*	FBgn0259224
lgl_T10	3L	22242971	C-A	0.12	*olf413*	FBgn0037153
aura T5	3L	1755140	C-A	0.11	*Cht2*	FBgn0022702
lgl_T5	3L	3737661	C-A	0.11	*CG32264*	FBgn0052264

mbtL1 T10	3L	17495013	C-A	0.16	*Oatp74D*	FBgn0036732
aura T5	3L	17360312	C-A	0.11	*tap*	FBgn0015550
lgl_T10	3L	21166648	G-T	0.24	*Ac78C*	FBgn0024150
lgl_T10	3L	7730141	C-A	0.11	*CG8492*	FBgn0035813
aura T5	3L	12531552	C-A	0.12	*Tsf2*	FBgn0036299
aura T5	3L	19148440	G-T	0.14	*fz2*	FBgn0016797
aura T5	3L	5455499	C-T	0.15		
lgl_T10	3L	805716	C-A	0.12		
lgl_T5	3L	15846121	G-T	0.12		
aura T5	3L	1234813	C-A	0.12	*CG33966*	FBgn0053966
mbtL2 T5	3L	6586345	G-T	0.25	*CG18769*	FBgn0042185
lgl_T10	3L	20261723	G-T	0.24	*rdgC*	FBgn0265959
aura T5	3L	461867	C-A	0.50	*klar*	FBgn0001316
lgl_T5	3L	19247054	G-T	0.10		
aura T5	3L	1498733	G-T	0.11	*pUf68*	FBgn0028577
lgl_T5	3L	10468029	C-A	0.12	*S-Lap3*	FBgn0045770
lgl_T10	3L	12586676	G-T	0.17	*ara*	FBgn0015904
lgl_T10	3L	11540104	G-T	0.12	*Mob2*	FBgn0259481
bratL1 T10	3L	24534569	T-A	0.25	*CR12460*	FBgn0040045
mbtL2 T0	3L	24534154	T-A	0.13	*CR12460*	FBgn0040045
lgl_T10	3L	4042902	G-T	0.13		
aura T5	3L	18904138	C-A	0.13	*CSN1b*	FBgn0027057
mbtL2 T0	3L	25503394	A-C	0.14	*CG45782*	FBgn0267429
mbtL2 T10B	3L	25503321	G-A	0.24	*CG45782*	FBgn0267429
lgl_T5	3L	13670857	C-A	0.10	*bru-3*	FBgn0264001
mbtL2 T0	3L	24308454	T-C	0.14		
lgl_T10	3L	12054633	G-T	0.24	*rols*	FBgn0041096
mbtL2 T10A	3L	25496611	A-T	0.13	*CG45782*	FBgn0267429
mbtL2 T5	3L	6352643	G-T	0.10		
mbtL1 T10	3L	27390459	T-G	0.11	*Dbp80*	FBgn0024804
aura T5	3L	3405460	G-T	0.18	*sty*	FBgn0014388
lgl_T10	3L	20028220	C-A	0.14	*CG14184*	FBgn0036932
lgl_T10	3L	1929044	A-G	1.00	*Dbx*	FBgn0261723
aura T5	3L	15068330	G-T	0.13	*cp309*	FBgn0086690
lgl_T10	3L	7483118	C-A	0.10		
aura T5	3L	6300892	C-A	0.14		
mbtL1 T5	3L	14080127	G-T	0.12	*btl*	FBgn0005592
lgl_T10	3L	18892480	G-T	0.12	*NijB*	FBgn0036822
mbtL1 T5	3L	13549206	C-A	0.12	*bru-3*	FBgn0264001
lgl_T10	3L	3182506	C-A	0.10	*Girdin*	FBgn0035411
lgl_T10	3L	24513372	G-A	0.11		
lgl_T5	3L	16050439	C-T	0.80	*Diap1*	FBgn0260635
aura T5	3L	13684813	G-T	0.14	*bru-3*	FBgn0264001
lgl_T10	3L	2130130	C-A	0.11	*zormin*	FBgn0052311
aura T5	3L	3149663	C-A	0.10	*BtbVII*	FBgn0263108
lgl_T10	3L	4241976	C-A	0.10	*CG14997*	FBgn0035515
lgl_T5	3L	14184403	G-T	0.14		
aura T5	3L	19812513	C-A	0.17	*Cyp305a1*	FBgn0036910
lgl_T5	3L	15611019	G-T	0.17	*CG13449*	FBgn0036520
lgl_T5	3L	11323658	C-A	0.15	*CR44721*	FBgn0265932
lgl_T5	3L	3097769	G-T	0.22	*Cht7*	FBgn0035398
lgl_T5	3L	1141956	C-A	0.13	*bab2*	FBgn0025525
mbtL1 T0	3L	9657008	C-G	0.11	*CNMaR*	FBgn0053696
lgl_T10	3L	16236129	G-T	0.11	*l(3)72Dp*	FBgn0263607
lgl_T10	3L	7272784	G-T	0.25	*unc-13-4A*	FBgn0035756
lgl_T5	3L	7804038	G-T	0.11	*CG32369*	FBgn0052369
lgl_T5	3L	4390922	G-T	0.10	*slow*	FBgn0035539
mbtL1 T5	3L	20833346	C-A	0.10	*Pka-R1*	FBgn0259243
mbtL1 T5	3L	23064343	C-A	0.41		
mbtL2 T5	3L	14496756	G-T	0.18	*bbg*	FBgn0087007
lgl_T5	3L	21348134	G-T	0.11	*ebd2*	FBgn0037076
lgl_T5	3L	14436496	C-A	0.15	*bbg*	FBgn0087007
lgl_T10	3L	16408378	C-A	0.12	*fax*	FBgn0014163

lgl_T5	3L	22011683	G-T	0.12		
lgl_T5	3L	954475	G-T	0.12	*Glut1*	FBgn0264574
lgl_T5	3L	8510326	C-A	0.12	*CG6745*	FBgn0035901
lgl_T10	3L	8508391	G-T	0.14	*ZC3H3*	FBgn0035900
lgl_T10	3L	13462160	C-A	0.11	*CG10738*	FBgn0036368
lgl_T10	3L	8349467	C-A	0.16	*GAPcenA*	FBgn0035879
mbtL1 T5	3L	8694634	G-T	0.17		
aura T5	3L	6090682	C-A	0.13	*CG13293*	FBgn0035677
lgl_T10	3L	3817257	C-A	0.08	*Scsalpha*	FBgn0004888
lgl_T10	3L	11819566	C-A	0.15	*CG5946*	FBgn0036211
lgl_T5	3L	16196807	G-T	0.19	*CR45437*	FBgn0266986
lgl_T5	3L	17466135	G-T	0.12	*CG7603*	FBgn0036726
mbtL1 T5	3L	22186494	G-T	0.12	*olf413*	FBgn0037153
aura T5	3L	12671400	C-A	0.09		
lgl_T10	3L	19231039	G-T	0.15	*fz2*	FBgn0016797
lgl_T10	3L	19091358	G-T	0.10		
lgl_T10	3L	563038	T-A	0.13	*hipk*	FBgn0035142
aura T5	3L	5591397	C-A	0.13	*spo*	FBgn0003486
mbtL1 T5	3L	12501003	G-T	0.13	*CG10638*	FBgn0036290
lgl_T10	3L	19363219	C-A	0.20	*CG32206*	FBgn0052206
aura T5	3L	9017782	G-T	0.12	*Doc2*	FBgn0035956
lgl_T5	3L	25322323	A-G	0.18	*CG45782*	FBgn0267429
aura T5	3L	8289320	C-A	0.11	*cert*	FBgn0027569
aura T5	3L	7543902	G-T	0.16		
lgl_T5	3L	19797924	C-A	0.11	*HLH106*	FBgn0261283
lgl_T5	3L	5944852	G-T	0.18	*CG33993*	FBgn0053993
lgl_T5	3L	17320370	G-T	0.16	*CG6485*	FBgn0036706
lgl_T10	3L	13303714	G-T	0.15	*CR44555*	FBgn0265748
aura T5	3L	22511116	G-T	0.13		
mbtL1 T10	3L	1006346	C-A	0.12	*trio*	FBgn0024277
lgl_T5	3L	25191279	G-C	0.15		
lgl_T10	3L	3345952	C-A	0.13	*kst*	FBgn0004167
lgl_T10	3L	15682890	C-A	0.12	*CG7579*	FBgn0036528
mbtL1 T5	3L	25612139	T-G	0.11	*CG45782*	FBgn0267429
mbtL2 T0	3L	25611345	G-A	0.14	*CG45782*	FBgn0267429
lgl_T5	3L	21174402	G-T	0.10	*chb*	FBgn0021760
aura T5	3L	19251493	C-A	0.22		
mbtL1 T10	3L	22145436	G-T	0.18	*olf413*	FBgn0037153
aura T5	3L	1000865	G-T	0.17	*trio*	FBgn0024277
aura T5	3L	1856257	G-T	0.24		
lgl_T10	3L	11641169	C-A	0.12	*CG32091*	FBgn0052091
mbtL1 T5	3L	15135603	C-A	0.18	*CG12316*	FBgn0036483
lgl_T5	3L	21791464	G-T	0.12		
aura T5	3L	8515310	C-A	0.15	*CG6765*	FBgn0035903
lgl_T10	3L	10975425	G-T	0.12		
aura T5	3L	22172416	G-T	0.11	*olf413*	FBgn0037153
mbtL2 T0	3L	25278315	A-G	0.41	*CG45782*	FBgn0267429
lgl_T5	3L	1229799	G-T	0.13	*mwh*	FBgn0264272
lgl_T5	3L	4893930	G-T	0.17	*CG17150*	FBgn0035581
lgl_T5	3L	2596282	C-A	0.19	*CG16984*	FBgn0062517
lgl_T5	3L	14264209	G-T	0.17		
lgl_T5	3L	3177406	C-A	0.13	*CG14964*	FBgn0035410
lgl_T5	3L	22091268	C-A	0.11		
mbtL1 T5	3L	2773857	G-T	0.20	*CG12093*	FBgn0035372
mbtL2 T5	3L	8447316	C-A	0.14	*CG6983*	FBgn0035896
lgl_T5	3L	4467356	C-A	0.09		
lgl_T5	3L	25398209	C-A	0.15	*CG45782*	FBgn0267429
mbtL2 T10B	3L	20212194	A-C	0.57		
lgl_T10	3L	11050650	G-T	0.21	*CG43693*	FBgn0263776
aura T5	3L	8420474	C-A	0.10	*CG32354*	FBgn0052354
lgl_T10	3L	1714092	C-A	0.11	*CG7991*	FBgn0035260
aura T5	3L	22080145	C-A	0.16		
lgl_T5	3L	19670596	C-A	0.23		

lgl_T5	3L	7113061	C-A	0.15	*form3*	FBgn0053556
aura T0	3L	21800302	C-A	0.24		
lgl_T10	3L	1203940	C-A	0.10	*CG9119*	FBgn0035189
lgl_T10	3L	2191343	G-T	0.25	*CG5707*	FBgn0026593
lgl_T5	3L	15417469	G-T	0.24		
aura T5	3L	22232249	G-T	0.11	*olf413*	FBgn0037153
lgl_T5	3L	4806414	G-T	0.15	*CG18808*	FBgn0042131
aura T5	3L	8347360	C-A	0.14	*GAPcenA*	FBgn0035879
lgl_T5	3L	784695	G-T	0.12		
mbtL1 T0	3L	9712791	C-A	0.12	*SH3PX1*	FBgn0040475
aura T5	3L	19867837	C-A	0.26	*Rab8*	FBgn0262518
aura T5	3L	9071109	G-T	0.10	*CG4911*	FBgn0035959
mbtL2 T5	3L	8368180	A-G	0.50		
aura T5	3L	230681	G-T	0.20	*CR42862*	FBgn0262109
lgl_T10	3L	22685681	G-T	0.18		
lgl_T5	3L	21840732	C-A	0.24	*P5CDh1*	FBgn0037138
lgl_T5	3L	728276	C-A	0.15		
lgl_T10	3L	3228533	G-T	0.11	*CG11505*	FBgn0035424
aura T5	3L	9868275	G-T	0.11	*CG8108*	FBgn0027567
lgl_T10	3L	18952416	G-T	0.12	*CG6836*	FBgn0036834
lgl_T5	3L	3221716	G-T	0.17	*RpL28*	FBgn0035422
lgl_T10	3L	20919467	G-T	0.15		
aura T5	3L	22306974	C-A	0.10	*Ten-m*	FBgn0004449
lgl_T10	3L	424629	C-A	0.14		
lgl_T5	3L	11364418	C-A	0.14	*CG6163*	FBgn0036155
aura T5	3L	22386453	C-A	0.15	*CR45962*	FBgn0267624
aura T5	3L	3188969	T-A	0.08	*CG42525*	FBgn0260430
aura T5	3L	22346067	C-A	0.13	*CR45963*	FBgn0267625
lgl_T5	3L	9252054	G-T	0.17	*GluRIB*	FBgn0264000
mbtL2 T10B	3L	21562620	C-A	0.14		
lgl_T10	3L	10005967	G-T	0.15	*dpr6*	FBgn0040823
lgl_T5	3L	5817633	G-T	0.11	*vn*	FBgn0003984
aura T5	3L	1269140	G-T	0.16	*CG9134*	FBgn0035199
aura T5	3L	22264886	G-T	0.10	*Ddx1*	FBgn0015075
lgl_T5	3L	9284089	G-T	0.15	*GluRIB*	FBgn0264000
lgl_T5	3L	5976131	G-T	0.15		
lgl_T10	3L	11670059	C-A	0.25	*CG32085*	FBgn0052085
lgl_T10	3L	1641314	G-T	0.11	*alphaCOP*	FBgn0025725
aura T5	3L	22537947	C-A	0.11		
mbtL1 T5	3L	21239062	G-T	0.16	*Eip78C*	FBgn0004865
mbtL1 T5	3L	20654852	G-T	0.10		
mbtL2 T5	3L	18315982	G-T	0.11		
lgl_T10	3L	24534113	T-C	0.21	*CR12460*	FBgn0040045
lgl_T5	3L	19016822	C-A	0.14	*nkd*	FBgn0002945
lgl_T10	3L	11835853	C-A	0.11	*CG43064*	FBgn0262366
lgl_T10	3L	18908052	G-T	0.13	*CG6841*	FBgn0036828
lgl_T5	3L	6345398	C-A	0.16	*CG13300*	FBgn0035699
bratL2 T0	3L	8751665	C-T	0.11	*CR44527*	FBgn0265720
lgl_T5	3L	11860580	C-A	0.13	*CG44837*	FBgn0266100
mbtL1 T10	3L	13879718	C-A	0.10	*CG13737*	FBgn0036382
lgl_T5	3L	9938444	G-A	0.75	*CG34356*	FBgn0085385
lgl_T10	3L	5658722	C-A	0.11	*sif*	FBgn0085447
lgl_T5	3L	13680666	C-A	0.11	*bru-3*	FBgn0264001
bratL2 T0	3L	8738665	A-C	0.19	*Prm*	FBgn0003149
mbtL1 T5	3L	17509690	G-T	0.19	*Oatp74D*	FBgn0036732
lgl_T10	3L	2198920	C-A	0.15		
bratL1 T0	3L	13789668	C-A	0.47	*bru-3*	FBgn0264001
mbtL2 T10A	3L	25503321	G-A	0.22	*CG45782*	FBgn0267429
bratL2 T0	3L	8726689	G-C	0.15		
mbtL1 T10	3L	25508614	T-C	0.10	*CG45782*	FBgn0267429
lgl_T5	3L	2905859	C-A	0.14	*Shab*	FBgn0262593
mbtL2 T0	3L	8727075	T-C	0.15	*Cp18*	FBgn0000357
lgl_T5	3L	13684310	G-T	0.12	*bru-3*	FBgn0264001

bratL2 T0	3L	8729963	C-T	0.13	*Cp19*	FBgn0000358
bratL2 T0	3L	8719160	C-G	0.75	*SrpRbeta*	FBgn0011509
bratL2 T0	3L	8715600	C-T	0.09		
lgl_T5	3L	6330637	C-A	0.11	*CG13300*	FBgn0035699
bratL2 T0	3L	8720932	T-C	0.17	*CG32022*	FBgn0052022
lgl_T0	3L	25191279	G-C	0.11		
mbtL2 T0	3L	8728204	C-G	0.16		
bratL2 T0	3L	8716239	T-C	0.12		
mbtL2 T10B	3L	24534557	C-T	0.11	*CR12460*	FBgn0040045
mbtL2 T0	3L	8727302	T-C	0.20	*Cp18*	FBgn0000357
bratL1 T10	3L	22198582	A-T	0.69	*olf413*	FBgn0037153
mbtL2 T0	3L	25190093	C-T	0.16		
mbtL2 T10A	3L	25503394	A-C	0.22	*CG45782*	FBgn0267429
lgl_T0	3L	25191324	C-A	0.13		
lgl_T5	3L	25191324	C-A	0.17		
mbtL1 T0	3L	23064392	A-G	0.29		
bratL1 T10	3L	24534593	T-G	0.24	*CR12460*	FBgn0040045
bratL1 T5	3L	24534593	T-G	0.15	*CR12460*	FBgn0040045
mbtL2 T10A	3L	24534154	T-A	0.21	*CR12460*	FBgn0040045
mbtL2 T10B	3L	24534154	T-A	0.28	*CR12460*	FBgn0040045
mbtL2 T5	3L	24534154	T-A	0.18	*CR12460*	FBgn0040045
mbtL1 T0	3L	23064366	T-C	0.28		
mbtL1 T5	3L	23064366	T-C	0.39		
aura T0	3L	25496633	G-T	0.16	*CG45782*	FBgn0267429
lgl_T10	3L	24513394	C-T	0.11		
mbtL2 T10A	3L	25496633	G-T	0.16	*CG45782*	FBgn0267429
mbtL2 T0	3L	25611365	C-G	0.12	*CG45782*	FBgn0267429
mbtL2 T10A	3L	24534130	T-A	0.18	*CR12460*	FBgn0040045
mbtL2 T10B	3L	24534130	T-A	0.26	*CR12460*	FBgn0040045
mbtL2 T10A	3L	25825354	T-C	0.11	*CG40178*	FBgn0058178
lgl_T0	3L	25191291	A-G	0.11		
lgl_T5	3L	25191291	A-G	0.14		
mbtL1 T10	3L	27172809	C-T	0.13		
bratL1 T10	3L	24534600	C-T	0.23	*CR12460*	FBgn0040045
bratL1 T5	3L	24534600	C-T	0.15	*CR12460*	FBgn0040045
aura T0	3L	25496639	T-C	0.16	*CG45782*	FBgn0267429
bratL1 T0	3L	13789674	A-G	0.39	*bru-3*	FBgn0264001
bratL1 T5	3L	13789674	A-G	0.32	*bru-3*	FBgn0264001
mbtL2 T10A	3L	25496639	T-C	0.15	*CG45782*	FBgn0267429
mbtL2 T10B	3L	27387276	C-T	0.24	*Dbp80*	FBgn0024804
lgl_T5	3L	9938447	A-C	0.50	*CG34356*	FBgn0085385
bratL1 T0	3L	13782337	A-C	0.22	*bru-3*	FBgn0264001
bratL2 T0	3L	27172810	C-G	0.14		
bratL2 T0	3L	27172811	G-A	0.14		
lgl_T5	3L	25398210	A-T	0.15	*CG45782*	FBgn0267429
mbtL1 T10	3L	27172810	C-G	0.13		
mbtL1 T10	3L	27172811	G-A	0.12		
bratL2 T5	3L	11733856	T-C	0.15	*CG6024*	FBgn0036202
bratL1 T10	3L	13773705	T-A	0.60	*bru-3*	FBgn0264001
bratL2 T0	3L	8712762	G-A	0.14		
mbtL1 T0	3L	9469958	T-G	0.38	*CG44838*	FBgn0266101
bratL1 T5	3L	5323445	T-C	0.19		
lgl_T0	3L	9239996	G-T	0.16		
mbtL2 T0	3L	2069394	C-T	0.08	*sls*	FBgn0086906
mbtL1 T10	3L	885181	G-T	0.16	*CG32479*	FBgn0052479
mbtL2 T5	3L	954474	G-T	0.10	*Glut1*	FBgn0264574
aura T5	3L	180076	G-T	0.12	*CG13875*	FBgn0035104
mbtL2 T10B	3L	914349	T-G	0.50	*Glut1*	FBgn0264574
mbtL1 T5	3L	693901	G-T	0.11	*CG13894*	FBgn0035157
mbtL2 T10A	3L	639396	G-T	0.13	*CR43334*	FBgn0263039
lgl_T10	3L	383799	C-A	0.15	*trh*	FBgn0262139
lgl_T5	3L	123309	C-A	0.11		
aura T0	3L	6342403	C-A	0.28	*CG13300*	FBgn0035699

bratL1 T0	3R	24799903	C-G	0.80		
bratL1 T5	3R	19192001	G-A	0.33	*CR46039*	FBgn0267706
bratL2 T5	3R	19686055	G-A	0.21	*Naam*	FBgn0051216
lgl_T0	3R	21292289	C-A	0.12	*SIFaR*	FBgn0038880
bratL2 T0	3R	16992355	C-T	0.18	*CR45221*	FBgn0266748
bratL1 T5	3R	27227913	G-T	0.18	*CG42813*	FBgn0261995
mbtL2 T0	3R	10799449	A-T	0.11	*CG14692*	FBgn0037836
aura T0	3R	13658984	C-A	0.16	*Hrb87F*	FBgn0004237
mbtL1 T0	3R	22259277	G-T	0.12	*SKIP*	FBgn0051163
aura T0	3R	23368532	C-A	0.17		
bratL1 T10	3R	26628535	C-G	0.10	*TwdlK*	FBgn0039439
bratL1 T10	3R	8696236	C-A	0.31	*hb*	FBgn0001180
bratL1 T10	3R	14471209	G-C	0.67	*cv-c*	FBgn0086901
bratL1 T10	3R	20778468	A-T	0.21	*Syp*	FBgn0038826
lgl_T0	3R	30545688	G-T	0.23	*tmod*	FBgn0082582
bratL2 T5	3R	28829301	C-G	0.10	*WASp*	FBgn0024273
mbtL1 T0	3R	10588907	T-A	0.88	*hth*	FBgn0001235
mbtL2 T10B	3R	12579429	C-A	0.30	*Octbeta2R*	FBgn0038063
mbtL1 T10	3R	21055902	C-A	0.10	*Mvl*	FBgn0011672
lgl_T0	3R	8149608	C-A	0.17	*grn*	FBgn0001138
mbtL1 T0	3R	15183837	G-T	0.17	*Gyc88E*	FBgn0038295
mbtL2 T10A	3R	11794556	C-A	0.09	*Sbf*	FBgn0025802
mbtL2 T10B	3R	16631491	C-A	0.11	*Fas1*	FBgn0262742
lgl_T0	3R	25125578	T-G	0.36	*vig2*	FBgn0046214
bratL2 T5	3R	23345593	T-G	0.20	*pnt*	FBgn0003118
mbtL2 T5	3R	9470171	C-A	0.10	*CG16779*	FBgn0037698
aura T0	3R	6174358	G-T	0.11	*Osi1*	FBgn0037406
bratL1 T5	3R	30708297	A-C	0.67		
aura T0	3R	26822337	T-A	1.00	*Tl*	FBgn0262473
mbtL2 T5	3R	17356781	C-A	0.12	*beat-IIa*	FBgn0038498
mbtL1 T10	3R	12981461	G-T	0.11	*Paip2*	FBgn0038100
mbtL2 T10A	3R	14850090	G-T	0.27	*btsz*	FBgn0266756
lgl_T0	3R	3617130	C-T	0.15		
mbtL1 T10	3R	6000741	T-G	0.14	*Rm62*	FBgn0003261
mbtL2 T10A	3R	25125520	G-T	0.17	*vig2*	FBgn0046214
mbtL1 T10	3R	15633623	C-A	0.21		
mbtL2 T0	3R	13835357	G-T	0.14	*Nsf2*	FBgn0266464
mbtL2 T5	3R	4466324	G-T	0.21	*CG31523*	FBgn0051523
mbtL2 T0	3R	17349132	C-A	0.12	*beat-IIa*	FBgn0038498
mbtL1 T5	3R	20063849	G-T	0.10		
mbtL2 T10B	3R	24198153	G-T	0.10	*twin*	FBgn0011725
mbtL2 T0	3R	23175447	C-A	0.13	*Irk1*	FBgn0265042
mbtL1 T0	3R	5604849	T-A	0.16	*asl*	FBgn0261004
mbtL2 T10B	3R	31227325	C-G	0.44	*CG12054*	FBgn0039831
mbtL1 T5	3R	5065922	G-T	0.12		
mbtL2 T10B	3R	18734746	T-A	0.28	*Mekk1*	FBgn0024329
mbtL1 T10	3R	8664880	C-A	0.11	*CG8032*	FBgn0037606
bratL2 T5	3R	31525566	C-G	0.13		
mbtL2 T0	3R	25125520	G-T	0.33	*vig2*	FBgn0046214
mbtL2 T10A	3R	18734746	T-A	0.49	*Mekk1*	FBgn0024329
mbtL1 T10	3R	22947340	G-T	0.10	*klg*	FBgn0017590
lgl_T10	3R	2694705	C-A	0.14	*CG45783*	FBgn0267430
mbtL2 T5	3R	27888606	G-T	0.14	*CG34353*	FBgn0085382
mbtL2 T10A	3R	5273796	G-T	0.17	*mtd*	FBgn0013576
mbtL1 T5	3R	10180994	G-T	0.11	*CG12814*	FBgn0037796
mbtL2 T5	3R	24277766	G-T	0.10	*Orct*	FBgn0019952
mbtL1 T10	3R	29737184	C-A	0.11	*CG7598*	FBgn0039689
bratL1 T10	3R	31787809	A-T	0.53	*faf*	FBgn0005632
mbtL2 T5	3R	20545998	G-T	0.15	*Stat92E*	FBgn0016917
mbtL2 T10A	3R	28342280	T-A	0.44	*Moca-cyp*	FBgn0039581
mbtL1 T10	3R	2504999	G-A	0.12		
mbtL1 T5	3R	2504999	G-A	0.13		
bratL1 T0	3R	2505760	G-A	0.18		

mbtL1 T5	3R	13174023	G-T	0.35		
mbtL1 T0	3R	25127431	T-A	0.19		
mbtL2 T10A	3R	20585318	C-T	0.62	*CG16953*	FBgn0038809
mbtL2 T5	3R	10943684	G-T	0.24		
mbtL1 T10	3R	25147131	G-T	0.08		
mbtL2 T10A	3R	31227325	C-G	0.48	*CG12054*	FBgn0039831
mbtL2 T5	3R	31486328	G-T	0.11		
mbtL1 T0	3R	23625092	T-G	0.27	*CG31145*	FBgn0051145
lgl_T5	3R	3617130	C-T	0.16		
lgl_T10	3R	18378303	C-A	0.15	*CG7675*	FBgn0038610
mbtL2 T10A	3R	26721740	C-G	0.29	*CG6420*	FBgn0039451
lgl_T10	3R	4874325	G-T	0.15		
mbtL1 T10	3R	27927058	C-A	0.11	*mino*	FBgn0027579
mbtL2 T0	3R	19227835	C-A	0.11	*CG11703*	FBgn0038690
mbtL2 T0	3R	2270777	C-T	0.11	*CG45784*	FBgn0267431
mbtL2 T10A	3R	7154818	G-T	0.17	*MAGE*	FBgn0037481
aura T5	3R	26188118	G-T	0.11	*CG6073*	FBgn0039417
mbtL1 T5	3R	25189674	C-A	0.29	*lobo*	FBgn0083946
mbtL1 T5	3R	7027459	C-A	0.12		
mbtL2 T10A	3R	21713659	T-C	0.42	*CG31176*	FBgn0051176
bratL2 T5	3R	3906784	C-G	0.16		
bratL1 T10	3R	28683723	C-G	0.36	*CG9990*	FBgn0039594
mbtL2 T5	3R	5924451	G-T	0.10	*CG34113*	FBgn0083949
mbtL2 T10B	3R	21658345	G-T	0.07	*E2f1*	FBgn0011766
lgl_T5	3R	1866915	G-T	0.17	*CG45784*	FBgn0267431
aura T5	3R	22292029	G-T	0.10	*SKIP*	FBgn0051163
mbtL2 T5	3R	30110644	A-G	0.44	*sima*	FBgn0266411
mbtL2 T10B	3R	5359224	C-A	0.16	*Cerk*	FBgn0037315
aura T0	3R	15339718	G-T	0.21	*CG6218*	FBgn0038321
mbtL2 T5	3R	12282041	C-A	0.08	*svp*	FBgn0003651
aura T5	3R	7584473	G-T	0.16	*CG34127*	FBgn0083963
lgl_T10	3R	28955985	G-T	0.18	*CG14521*	FBgn0039617
mbtL2 T5	3R	18348347	G-T	0.07	*CG15803*	FBgn0038606
lgl_T10	3R	30842292	G-T	0.17		
mbtL1 T10	3R	31389836	T-G	0.53	*CG1607*	FBgn0039844
aura T0	3R	16309960	C-A	0.14		
mbtL2 T0	3R	14804875	C-A	0.13	*btsz*	FBgn0266756
mbtL2 T5	3R	22459782	C-A	0.07	*CG34376*	FBgn0085405
mbtL2 T10A	3R	16839236	T-C	0.50	*iab-8*	FBgn0264857
mbtL1 T5	3R	22262682	G-T	0.17	*SKIP*	FBgn0051163
mbtL2 T5	3R	21485433	G-T	0.10	*CG7922*	FBgn0038889
lgl_T10	3R	3617130	C-T	0.21		
mbtL1 T5	3R	17565475	G-T	0.10	*Hmx*	FBgn0264005
aura T5	3R	14279561	G-T	0.24	*trx*	FBgn0003862
mbtL2 T10B	3R	4307661	G-T	0.16		
mbtL1 T5	3R	7918697	G-T	0.12	*CG10445*	FBgn0037531
mbtL2 T10B	3R	6846631	G-T	0.10	*Scr*	FBgn0003339
lgl_T10	3R	19255241	G-T	0.11		
aura T5	3R	9587335	G-T	0.11	*P58IPK*	FBgn0037718
mbtL2 T10B	3R	27908369	G-T	0.13		
mbtL2 T0	3R	20725574	C-A	0.13		
mbtL2 T10A	3R	29802511	G-T	0.16	*CG7834*	FBgn0039697
lgl_T10	3R	6307971	C-A	0.10	*CG46026*	FBgn0267690
bratL1 T10	3R	29487034	A-C	0.67	*Ptp99A*	FBgn0004369
mbtL2 T10B	3R	20078371	C-A	0.15		
lgl_T10	3R	14806564	G-C	0.31	*btsz*	FBgn0266756
aura T5	3R	30207686	C-A	0.12	*RpS7*	FBgn0039757
aura T5	3R	27608315	C-A	0.12	*Tusp*	FBgn0039530
mbtL1 T10	3R	25922285	G-T	0.14	*CG14546*	FBgn0039395
mbtL2 T5	3R	25895020	C-A	0.18	*CG5913*	FBgn0039385
mbtL1 T10	3R	23716724	C-A	0.21	*nau*	FBgn0002922
lgl_T5	3R	4380776	C-A	0.17	*CG11739*	FBgn0037239
aura T5	3R	12947579	G-T	0.14		

aura T5	3R	11170070	C-A	0.13	*Ugt35b*	FBgn0026314
lgl_T5	3R	19984531	A-C	0.27	*Hs6st*	FBgn0038755
lgl_T10	3R	13859390	C-A	0.10		
aura T5	3R	19184145	C-A	0.17	*CG31475*	FBgn0051475
mbtL2 T10A	3R	1867095	C-G	0.13	*CG45784*	FBgn0267431
mbtL2 T10B	3R	1867090	A-C	0.19	*CG45784*	FBgn0267431
mbtL1 T0	3R	25858671	G-A	0.32	*CG5890*	FBgn0039380
lgl_T10	3R	19984531	A-C	0.17	*Hs6st*	FBgn0038755
mbtL1 T5	3R	28210090	G-T	0.21	*CG34354*	FBgn0085383
mbtL1 T10	3R	9382074	T-G	0.19	*FER*	FBgn0000723
mbtL2 T10B	3R	26721740	C-G	0.41	*CG6420*	FBgn0039451
lgl_T5	3R	18513911	G-T	0.11	*fru*	FBgn0004652
mbtL2 T10A	3R	22409594	G-T	0.11	*CG13850*	FBgn0038961
bratL2 T5	3R	29518895	A-C	0.22		
mbtL2 T10A	3R	5959715	G-T	0.33		
mbtL2 T10B	3R	7669742	C-A	0.37	*Nlg1*	FBgn0051146
mbtL1 T10	3R	16315844	G-T	0.11	*Sulf1*	FBgn0040271
lgl_T5	3R	24828107	G-T	0.11	*RabX4*	FBgn0051118
mbtL2 T5	3R	12996825	C-A	0.08	*CG7488*	FBgn0038106
lgl_T5	3R	31124781	G-T	0.20		
lgl_T10	3R	27136801	C-A	0.48	*CG6051*	FBgn0039492
mbtL2 T0	3R	19878236	C-A	0.12	*Arc42*	FBgn0038742
bratL1 T10	3R	15120091	G-T	0.50	*CG42788*	FBgn0261859
aura T5	3R	25002659	G-T	0.12	*tnc*	FBgn0039257
mbtL2 T0	3R	17989204	C-A	0.14	*cpo*	FBgn0263995
aura T5	3R	6029567	C-A	0.21		
aura T0	3R	14297513	G-T	0.17	*CG12207*	FBgn0038220
lgl_T5	3R	28938533	C-A	0.15	*CG14521*	FBgn0039617
lgl_T10	3R	11198435	G-T	0.17	*cu*	FBgn0261808
lgl_T10	3R	15501930	C-A	0.13		
mbtL1 T5	3R	11651295	G-T	0.13	*Elp1*	FBgn0037926
aura T5	3R	8214163	G-T	0.17		
aura T5	3R	20761660	G-T	0.14	*Syp*	FBgn0038826
lgl_T10	3R	9258362	C-G	0.41	*Ibf2*	FBgn0037669
lgl_T5	3R	23698796	G-T	0.13	*prt*	FBgn0043005
mbtL1 T5	3R	20720036	G-T	0.14		
mbtL2 T10A	3R	28966556	A-G	0.43	*CG14521*	FBgn0039617
mbtL2 T10B	3R	28966556	A-G	0.60	*CG14521*	FBgn0039617
aura T5	3R	4221746	C-A	0.11	*aux*	FBgn0037218
lgl_T5	3R	29849926	G-T	0.12	*Cad99C*	FBgn0039709
mbtL2 T10B	3R	5959715	G-T	0.52		
lgl_T5	3R	27130980	G-T	0.11	*CG6051*	FBgn0039492
bratL1 T10	3R	27227913	G-T	0.77	*CG42813*	FBgn0261995
aura T5	3R	17681684	C-A	0.14	*alt*	FBgn0038535
mbtL2 T10A	3R	15879042	G-T	0.15		
bratL1 T10	3R	30073499	T-A	0.63	*sima*	FBgn0266411
lgl_T5	3R	5429473	G-T	0.19	*CG14669*	FBgn0037326
mbtL1 T5	3R	13758880	C-A	0.10	*CG34383*	FBgn0085412
mbtL1 T5	3R	24031079	G-T	0.15	*KrT95D*	FBgn0020647
lgl_T5	3R	26006853	G-T	0.14	*CR45039*	FBgn0266399
aura T5	3R	30790219	G-T	0.22	*wts*	FBgn0011739
lgl_T5	3R	14142986	G-T	0.12		
mbtL1 T5	3R	16654390	G-T	0.20	*modSP*	FBgn0051217
mbtL1 T10	3R	26499457	C-A	0.14	*plum*	FBgn0039431
mbtL2 T10B	3R	26008738	G-T	0.06		
mbtL2 T5	3R	28966556	A-G	0.46	*CG14521*	FBgn0039617
bratL1 T0	3R	970361	T-A	0.13	*CG45784*	FBgn0267431
mbtL1 T5	3R	30385989	G-T	0.11	*Fer1HCH*	FBgn0015222
mbtL1 T5	3R	25858671	G-A	0.29	*CG5890*	FBgn0039380
mbtL1 T5	3R	29303911	C-A	0.11		
mbtL1 T10	3R	6562526	C-A	0.14	*dpr11*	FBgn0053202
lgl_T5	3R	16207150	G-T	0.11		
mbtL1 T5	3R	15100561	C-A	0.11	*CG42788*	FBgn0261859

mbtL1 T5	3R	26604724	G-T	0.21		
mbtL1 T5	3R	22806639	C-A	0.12	*CG7031*	FBgn0039027
lgl_T5	3R	10412490	G-T	0.17	*Irp-1B*	FBgn0024957
lgl_T10	3R	22822416	G-T	0.12		
mbtL2 T10B	3R	19275834	G-T	0.14		
aura T5	3R	4845543	G-T	0.12		
mbtL1 T5	3R	16075866	G-T	0.12	*Mhcl*	FBgn0026059
mbtL2 T10A	3R	3412217	C-G	0.16		
mbtL2 T10B	3R	3412217	C-G	0.33		
mbtL2 T5	3R	18883295	C-A	0.09		
mbtL2 T5	3R	13982297	G-T	0.07	*rdx*	FBgn0264493
lgl_T10	3R	10103654	C-A	0.19	*Fmr1*	FBgn0028734
lgl_T5	3R	22552041	C-A	0.11	*bond*	FBgn0260942
lgl_T5	3R	21365974	C-A	0.10	*pre-mod(mdg4)-V*	FBgn0261844
mbtL2 T5	3R	24798362	G-T	0.10	*Ude*	FBgn0039226
mbtL2 T10B	3R	25125520	G-T	0.16	*vig2*	FBgn0046214
mbtL1 T5	3R	29819907	C-T	0.35	*neo*	FBgn0039704
mbtL2 T10A	3R	7669742	C-A	0.59	*Nlg1*	FBgn0051146
lgl_T5	3R	8435339	C-A	0.12	*CG45263*	FBgn0266801
lgl_T10	3R	24010035	G-T	0.15	*KrT95D*	FBgn0020647
mbtL2 T10B	3R	20585318	C-T	0.52	*CG16953*	FBgn0038809
lgl_T0	3R	31210754	G-T	0.10		
lgl_T10	3R	16038254	G-T	0.19	*pnr*	FBgn0003117
mbtL2 T0	3R	2769085	G-T	0.14	*CG45783*	FBgn0267430
lgl_T10	3R	25125578	T-G	0.26	*vig2*	FBgn0046214
lgl_T10	3R	16848272	G-A	0.11	*iab-8*	FBgn0264857
lgl_T10	3R	21037380	G-T	0.11	*Dhc93AB*	FBgn0013812
mbtL1 T5	3R	28690807	C-A	0.15	*CG9990*	FBgn0039594
mbtL1 T5	3R	8396539	C-A	0.15	*CG45263*	FBgn0266801
mbtL1 T5	3R	5542485	C-A	0.10	*CG2519*	FBgn0037336
mbtL2 T10B	3R	31698471	G-T	0.12		
aura T5	3R	19861660	G-T	0.11	*CG11447*	FBgn0038737
lgl_T10	3R	25937297	G-T	0.12	*CCAP-R*	FBgn0039396
aura T5	3R	16820807	G-T	0.14	*abd-A*	FBgn0000014
mbtL2 T5	3R	13449444	G-T	0.17		
lgl_T5	3R	31702919	G-T	0.11		
mbtL1 T5	3R	27481050	C-A	0.17	*Cyp6a18*	FBgn0039519
lgl_T10	3R	21698048	C-A	0.11	*CG31176*	FBgn0051176
mbtL2 T0	3R	11241974	T-C	0.09	*scpr-A*	FBgn0037889
lgl_T10	3R	11640792	C-A	0.10	*Csk*	FBgn0262081
mbtL2 T10A	3R	15292385	A-G	0.12	*Tm1*	FBgn0003721
mbtL1 T5	3R	15539639	G-T	0.11	*AOX2*	FBgn0038348
lgl_T5	3R	17406772	G-T	0.18	*CG42798*	FBgn0261932
lgl_T5	3R	2302105	G-A	0.21	*CG45784*	FBgn0267431
mbtL1 T10	3R	30254015	C-A	0.10	*CG18404*	FBgn0039761
mbtL2 T10B	3R	28342280	T-A	0.48	*Moca-cyp*	FBgn0039581
aura T5	3R	18451587	C-A	0.19	*fru*	FBgn0004652
lgl_T5	3R	13058526	C-A	0.17	*sim*	FBgn0004666
lgl_T5	3R	4843710	C-A	0.13		
mbtL1 T5	3R	27034044	G-T	0.11	*RYa-R*	FBgn0004842
mbtL1 T5	3R	14317777	C-A	0.10	*pr-set7*	FBgn0011474
aura T5	3R	28297235	G-T	0.16		
lgl_T5	3R	6192164	C-A	0.09	*NPFR*	FBgn0037408
mbtL1 T5	3R	31802327	G-T	0.13	*Ir100a*	FBgn0039879
mbtL2 T10B	3R	24608914	C-A	0.15	*mld*	FBgn0263490
lgl_T5	3R	16612744	G-T	0.12	*CG12783*	FBgn0038448
mbtL1 T10	3R	2906632	A-T	0.43	*CG45783*	FBgn0267430
mbtL1 T5	3R	31389836	T-G	0.50	*CG1607*	FBgn0039844
lgl_T10	3R	8643581	C-A	0.10	*Cyp313b1*	FBgn0037601
mbtL1 T0	3R	2906632	A-T	0.24	*CG45783*	FBgn0267430
mbtL2 T5	3R	28279917	C-A	0.13		
lgl_T10	3R	13110039	C-A	0.24	*timeout*	FBgn0038118
lgl_T5	3R	9571251	G-T	0.11	*CG9396*	FBgn0037714

mbtL2 T5	3R	4847367	G-T	0.20		
lgl_T10	3R	7903786	G-T	0.12	*CG2943*	FBgn0037530
lgl_T10	3R	20363048	G-T	0.11		
aura T5	3R	21139383	G-T	0.14	*meigo*	FBgn0250820
mbtL1 T5	3R	23371009	G-T	0.12		
aura T5	3R	12191604	C-A	0.10		
lgl_T5	3R	23084961	C-A	0.12	*CG13830*	FBgn0039054
aura T5	3R	8717353	C-A	0.15	*CG8116*	FBgn0037614
lgl_T5	3R	27571251	G-T	0.15	*wdb*	FBgn0027492
lgl_T10	3R	23324002	C-A	0.15	*pnt*	FBgn0003118
mbtL2 T10A	3R	19099212	C-A	0.10	*CG6040*	FBgn0038679
lgl_T5	3R	24153415	C-A	0.10	*CG6178*	FBgn0039156
aura T5	3R	5389885	G-T	0.11	*kkv*	FBgn0001311
lgl_T5	3R	16970346	G-T	0.11	*Abd-B*	FBgn0000015
aura T5	3R	6384201	C-A	0.14	*godzilla*	FBgn0037442
bratL1 T10	3R	2860147	T-C	0.12	*CG45783*	FBgn0267430
bratL2 T5	3R	23696911	G-A	0.38	*prt*	FBgn0043005
lgl_T10	3R	25476444	C-A	0.17	*CG4553*	FBgn0039336
mbtL1 T0	3R	3256469	A-T	0.17	*CG45783*	FBgn0267430
lgl_T10	3R	26471662	G-T	0.11	*plum*	FBgn0039431
mbtL1 T10	3R	3256051	T-G	0.13	*CG45783*	FBgn0267430
bratL1 T10	3R	27577312	C-G	0.83	*wdb*	FBgn0027492
aura T5	3R	29317600	G-T	0.10	*Cnx99A*	FBgn0015622
aura T5	3R	15694923	G-T	0.17		
lgl_T10	3R	12720341	G-T	0.22	*Men*	FBgn0002719
mbtL2 T10B	3R	27058645	C-A	0.16	*Nep5*	FBgn0039478
lgl_T5	3R	11436240	C-A	0.16	*CG5281*	FBgn0037902
lgl_T10	3R	29841951	G-T	0.10	*Cad99C*	FBgn0039709
aura T5	3R	9918178	G-T	0.13	*Glut4EF*	FBgn0267336
mbtL2 T5	3R	25125520	G-T	0.17	*vig2*	FBgn0046214
mbtL1 T5	3R	30710273	G-T	0.12		
lgl_T10	3R	24631515	C-A	0.11	*atl*	FBgn0039213
aura T5	3R	28971759	G-T	0.12	*CR45669*	FBgn0267229
lgl_T10	3R	12126903	G-T	0.17	*CG4702*	FBgn0037992
lgl_T5	3R	7238309	C-A	0.14	*CG14608*	FBgn0037487
aura T5	3R	3617130	C-T	0.21		
aura T5	3R	16013632	C-A	0.17	*GATAe*	FBgn0038391
aura T5	3R	31375203	C-A	0.13		
lgl_T10	3R	9571264	G-T	0.14	*CG9396*	FBgn0037714
lgl_T5	3R	5776199	C-A	0.10	*CG2082*	FBgn0027608
lgl_T5	3R	6711098	G-T	0.13	*pb*	FBgn0051481
aura T5	3R	23700017	C-A	0.17	*CG31468*	FBgn0047351
lgl_T5	3R	29248897	G-T	0.19	*SP1029*	FBgn0263236
mbtL2 T10A	3R	25434814	C-G	0.21	*Fur1*	FBgn0004509
lgl_T5	3R	7544391	G-T	0.11	*alpha-Est2*	FBgn0015570
lgl_T5	3R	11832332	C-A	0.16	*CG6959*	FBgn0037956
mbtL1 T5	3R	10484835	C-A	0.18		
aura T5	3R	15101677	C-A	0.11	*CG42788*	FBgn0261859
lgl_T5	3R	25125578	T-G	0.28	*vig2*	FBgn0046214
mbtL2 T5	3R	11240450	G-T	0.10	*scpr-B*	FBgn0037888
lgl_T5	3R	12129489	C-A	0.17		
lgl_T5	3R	21912789	C-A	0.12	*CG6690*	FBgn0038918
lgl_T5	3R	15512610	C-A	0.15		
mbtL2 T10A	3R	2157579	A-T	0.57	*CG45784*	FBgn0267431
mbtL2 T10B	3R	2157579	A-T	0.38	*CG45784*	FBgn0267431
bratL2 T0	3R	3906784	C-G	0.12		
lgl_T10	3R	27855370	G-T	0.10	*CG34353*	FBgn0085382
aura T5	3R	16301367	G-T	0.15	*gish*	FBgn0250823
aura T5	3R	14566209	C-G	0.20	*CG14852*	FBgn0038242
bratL1 T0	3R	2791491	G-C	0.11	*CG45783*	FBgn0267430
mbtL1 T5	3R	30993134	G-T	0.10	*CR46117*	FBgn0267786
aura T5	3R	22960200	G-T	0.14	*klg*	FBgn0017590
lgl_T5	3R	30438585	G-T	0.11	*CG15537*	FBgn0039770

lgl_T5	3R	11102797	G-T	0.13		
mbtL1 T10	3R	9654784	T-G	0.21	*CG9492*	FBgn0037726
aura T5	3R	17092801	G-T	0.13	*cher*	FBgn0014141
lgl_T5	3R	17759609	C-A	0.22	*TyrR*	FBgn0038542
lgl_T10	3R	7290600	G-T	0.12	*rn*	FBgn0267337
aura T5	3R	31721596	G-T	0.14	*ttk*	FBgn0003870
lgl_T5	3R	12394649	G-T	0.15	*CG10097*	FBgn0038033
lgl_T10	3R	22281312	C-A	0.24	*SKIP*	FBgn0051163
aura T5	3R	11431011	G-T	0.12	*RpL24-like*	FBgn0037899
lgl_T10	3R	24307254	C-A	0.10	*crb*	FBgn0259685
lgl_T5	3R	7922677	G-T	0.13	*lds*	FBgn0002542
lgl_T10	3R	31837923	C-A	0.13	*RhoGAP100F*	FBgn0039883
lgl_T5	3R	20838686	C-A	0.13	*Synd*	FBgn0053094
lgl_T10	3R	12382154	C-A	0.15	*CG10035*	FBgn0038028
lgl_T5	3R	21619838	G-T	0.20		
mbtL2 T10A	3R	2411253	T-A	0.38	*CG45784*	FBgn0267431
mbtL2 T10B	3R	2411253	T-A	0.42	*CG45784*	FBgn0267431
lgl_T10	3R	6824182	G-T	0.09	*Scr*	FBgn0003339
mbtL1 T5	3R	2757080	C-A	0.13	*CG45783*	FBgn0267430
lgl_T10	3R	31436954	G-T	0.11	*Gprk2*	FBgn0261988
mbtL1 T5	3R	21309702	G-T	0.16	*Hsromega*	FBgn0001234
lgl_T5	3R	20504672	C-A	0.13		
mbtL2 T0	3R	25367593	G-T	0.31	*Fur1*	FBgn0004509
aura T5	3R	26446221	C-A	0.16	*tx*	FBgn0263118
lgl_T10	3R	8249061	G-T	0.16	*CG15864*	FBgn0040528
lgl_T5	3R	8897280	C-A	0.13	*pyd*	FBgn0262614
aura T5	3R	28592636	C-A	0.27		
aura T5	3R	10159860	G-T	0.13	*CG6254*	FBgn0037794
mbtL2 T10A	3R	2741476	T-A	0.45	*CG45783*	FBgn0267430
aura T5	3R	10415111	G-T	0.16	*CG6345*	FBgn0037816
mbtL2 T10B	3R	2741476	T-A	0.43	*CG45783*	FBgn0267430
lgl_T5	3R	12627873	G-T	0.14		
aura T5	3R	14799419	C-A	0.12	*CG14856*	FBgn0038261
mbtL1 T5	3R	14548518	G-T	0.13	*Pde6*	FBgn0038237
aura T5	3R	20090765	G-T	0.16		
lgl_T5	3R	14419043	C-A	0.15	*cv-c*	FBgn0086901
mbtL1 T5	3R	5881591	C-A	0.18	*CG34113*	FBgn0083949
lgl_T10	3R	17073110	C-A	0.11	*mRpS11*	FBgn0038474
lgl_T5	3R	26449813	G-T	0.13	*Hex-t1*	FBgn0042711
lgl_T5	3R	8658289	G-T	0.10	*bel*	FBgn0263231
aura T5	3R	27874480	G-T	0.20	*CG34353*	FBgn0085382
lgl_T5	3R	26226746	G-T	0.10		
lgl_T5	3R	10742846	G-T	0.13	*TkR86C*	FBgn0004841
lgl_T10	3R	21254322	C-A	0.17	*ETHR*	FBgn0038874
lgl_T10	3R	29433341	G-T	0.10	*Ptp99A*	FBgn0004369
aura T5	3R	22679608	C-A	0.14	*wake*	FBgn0266418
lgl_T5	3R	13563324	G-T	0.14		
mbtL1 T5	3R	20929569	C-A	0.11	*CG31191*	FBgn0051191
lgl_T5	3R	6919116	G-T	0.20	*Antp*	FBgn0260642
aura T5	3R	19391486	G-T	0.13	*Ino80*	FBgn0086613
lgl_T10	3R	27472701	G-T	0.14		
lgl_T5	3R	9786520	G-T	0.10	*CG8500*	FBgn0037754
aura T5	3R	24359836	C-A	0.15	*CG18528*	FBgn0039189
lgl_T5	3R	14756029	G-T	0.20	*jvl*	FBgn0263929
mbtL1 T5	3R	26059463	C-A	0.16		
aura T5	3R	11631484	C-A	0.12	*CG14712*	FBgn0037924
bratL1 T10	3R	2470935	T-G	0.14	*CG45784*	FBgn0267431
bratL1 T10	3R	15319559	C-A	0.15	*CG6276*	FBgn0038316
mbtL1 T5	3R	10681284	G-T	0.14		
lgl_T10	3R	10295511	C-A	0.15	*CG43143*	FBgn0262617
lgl_T5	3R	19233144	G-T	0.14	*unc79*	FBgn0038693
aura T5	3R	18020211	C-A	0.17	*DNaseII*	FBgn0000477
lgl_T10	3R	10561973	C-A	0.13	*hth*	FBgn0001235

aura T5	3R	23288785	C-A	0.13	*CG4434*	FBgn0039071
lgl_T10	3R	26122053	C-A	0.12	*CG31324*	FBgn0051324
lgl_T10	3R	20547729	G-T	0.25	*Stat92E*	FBgn0016917
lgl_T10	3R	22019223	C-A	0.31	*CG6439*	FBgn0038922
lgl_T5	3R	18697951	G-T	0.31		
lgl_T5	3R	27921872	C-A	0.16	*BCAS2*	FBgn0039558
aura T5	3R	6567272	C-A	0.10	*dpr11*	FBgn0053202
aura T5	3R	24156799	C-A	0.20	*Myo95E*	FBgn0039157
mbtL1 T5	3R	11019499	G-T	0.12	*CG34114*	FBgn0083950
mbtL2 T10B	3R	25304404	G-T	0.10	*CG31436*	FBgn0051436
lgl_T10	3R	23497382	G-T	0.12		
mbtL1 T10	3R	26672790	G-T	0.29	*beat-VII*	FBgn0250908
aura T5	3R	22465361	G-T	0.11	*CG7054*	FBgn0038972
lgl_T5	3R	27738701	G-T	0.13	*Ets98B*	FBgn0005659
aura T5	3R	26827839	G-T	0.17	*Tl*	FBgn0262473
lgl_T5	3R	18862946	C-A	0.15		
lgl_T5	3R	22714330	G-T	0.14	*wge*	FBgn0051151
aura T5	3R	23974336	C-A	0.16	*CG31140*	FBgn0051140
lgl_T0	3R	30707487	C-A	0.18		
lgl_T5	3R	25285977	G-T	0.12	*CG31104*	FBgn0051104
mbtL1 T10	3R	30413613	C-A	0.11		
mbtL1 T5	3R	10839427	G-T	0.18	*Cad86C*	FBgn0261053
bratL1 T10	3R	31945350	G-T	0.65	*heph*	FBgn0011224
lgl_T10	3R	14015395	C-A	0.12	*rdx*	FBgn0264493
lgl_T10	3R	5028545	G-T	0.14		
lgl_T10	3R	31113569	G-T	0.13		
aura T5	3R	31874203	G-T	0.14	*heph*	FBgn0011224
aura T5	3R	26597332	G-T	0.17	*scrib*	FBgn0263289
lgl_T5	3R	30043988	G-T	0.10	*Sry-delta*	FBgn0003512
lgl_T5	3R	9183736	C-A	0.15	*pum*	FBgn0003165
mbtL1 T5	3R	22953426	G-T	0.15	*klg*	FBgn0017590
aura T5	3R	15248257	C-A	0.18	*CG6499*	FBgn0038309
lgl_T10	3R	31580860	C-A	0.11		
lgl_T10	3R	11807025	C-T	0.15	*ClC-a*	FBgn0051116
aura T5	3R	23104005	G-T	0.13	*CG13830*	FBgn0039054
aura T5	3R	13263714	C-A	0.11	*Ravus*	FBgn0038128
mbtL2 T10B	3R	6986324	G-T	0.20	*Antp*	FBgn0260642
lgl_T10	3R	6571879	G-T	0.13	*dpr11*	FBgn0053202
lgl_T5	3R	9036048	C-A	0.19	*neur*	FBgn0002932
lgl_T5	3R	15650272	C-A	0.15		
aura T5	3R	31052949	G-T	0.13	*l(3)03670*	FBgn0010808
lgl_T10	3R	22958374	C-A	0.12	*klg*	FBgn0017590
aura T5	3R	8349914	G-T	0.10	*Atg13*	FBgn0261108
mbtL1 T5	3R	13894538	C-A	0.13		
lgl_T5	3R	14554041	C-A	0.20	*Pde6*	FBgn0038237
lgl_T5	3R	28167853	C-A	0.11	*CG34354*	FBgn0085383
mbtL2 T5	3R	31713770	C-T	0.29		
lgl_T5	3R	14962508	G-T	0.25		
lgl_T5	3R	28300376	G-T	0.14		
lgl_T10	3R	27266427	C-A	0.13	*l(3)mbt*	FBgn0002441
lgl_T10	3R	16357750	G-T	0.17	*CG17931*	FBgn0038421
mbtL2 T5	3R	26021095	C-A	0.12		
lgl_T10	3R	7519215	C-A	0.17	*Ubc84D*	FBgn0017456
aura T5	3R	30916092	C-A	0.13	*Ptx1*	FBgn0020912
aura T5	3R	17806359	G-T	0.10	*CG17801*	FBgn0038550
aura T5	3R	11819909	G-T	0.12	*CoVa*	FBgn0019624
lgl_T5	3R	31826536	C-A	0.18	*RhoGAP100F*	FBgn0039883
lgl_T5	3R	20106088	C-A	0.17		
lgl_T10	3R	16217035	C-A	0.11		
lgl_T5	3R	15082232	C-A	0.15	*CG6966*	FBgn0038286
lgl_T10	3R	30960482	C-A	0.10	*CG15548*	FBgn0039812
lgl_T10	3R	29217851	G-T	0.18	*CR31044*	FBgn0051044
aura T5	3R	13380453	G-T	0.13	*CG8870*	FBgn0038144

mbtL1 T5	3R	2873362	C-A	0.15	*CG45783*	FBgn0267430
mbtL2 T5	3R	28394062	G-T	0.22		
mbtL1 T5	3R	5656178	C-A	0.17	*MED27*	FBgn0037359
mbtL2 T10A	3R	1135010	A-T	0.57	*CG45784*	FBgn0267431
lgl_T10	3R	5345876	G-T	0.13	*mtd*	FBgn0013576
lgl_T5	3R	28033253	C-A	0.11	*CG34362*	FBgn0085391
lgl_T5	3R	22023920	G-T	0.11	*mRpL35*	FBgn0038923
lgl_T5	3R	13271830	C-A	0.25	*CG8449*	FBgn0038129
lgl_T5	3R	20257432	G-T	0.10	*cic*	FBgn0262582
lgl_T10	3R	7019274	C-A	0.10		
lgl_T5	3R	7662422	G-T	0.15	*Nlg1*	FBgn0051146
mbtL1 T5	3R	21062085	G-T	0.11	*Cortactin*	FBgn0025865
lgl_T10	3R	8009640	C-A	0.12	*CG3223*	FBgn0037538
lgl_T5	3R	13162617	G-T	0.12	*timeout*	FBgn0038118
mbtL1 T5	3R	25293692	G-T	0.19	*CG31102*	FBgn0051102
aura T5	3R	4949017	C-A	0.18	*Ubc6*	FBgn0004436
aura T5	3R	23803157	C-A	0.12	*CG33111*	FBgn0053111
aura T5	3R	15350246	C-A	0.13	*CG42727*	FBgn0261680
lgl_T10	3R	7393299	C-A	0.19		
lgl_T10	3R	29057099	C-A	0.12	*Sirt7*	FBgn0039631
aura T5	3R	23388160	G-T	0.11		
lgl_T10	3R	21834920	G-T	0.19		
lgl_T10	3R	5233913	C-A	0.11	*Mms19*	FBgn0037301
lgl_T5	3R	18960410	G-T	0.09	*CG42613*	FBgn0261262
lgl_T10	3R	27951619	C-A	0.15	*Gp93*	FBgn0039562
mbtL1 T10	3R	28022897	C-A	0.13		
aura T5	3R	13043115	C-A	0.17	*CG11670*	FBgn0038114
mbtL2 T5	3R	31579384	G-T	0.13	*pygo*	FBgn0043900
mbtL2 T10B	3R	2839832	C-G	0.35	*CG45783*	FBgn0267430
aura T5	3R	21231977	C-A	0.13	*e*	FBgn0000527
lgl_T5	3R	31918957	G-T	0.16	*heph*	FBgn0011224
lgl_T10	3R	27566764	C-G	0.45	*wdb*	FBgn0027492
lgl_T5	3R	11527752	C-A	0.10		
lgl_T5	3R	31215350	G-T	0.17	*CG15561*	FBgn0039829
lgl_T5	3R	23789167	G-T	0.11	*mbc*	FBgn0015513
lgl_T5	3R	6281749	C-A	0.13	*Osi12*	FBgn0037419
lgl_T10	3R	5472376	C-A	0.11	*Snr1*	FBgn0011715
lgl_T10	3R	6911530	C-A	0.13	*Antp*	FBgn0260642
mbtL2 T5	3R	29053649	C-A	0.08	*CG11842*	FBgn0039629
aura T5	3R	4308141	C-A	0.16	*MP1*	FBgn0027930
lgl_T10	3R	6432993	G-T	0.14	*gpp*	FBgn0264495
lgl_T5	3R	9871238	G-T	0.13		
mbtL1 T10	3R	29819907	C-T	0.46	*neo*	FBgn0039704
lgl_T5	3R	10825453	C-A	0.12		
aura T5	3R	5031116	G-T	0.13		
lgl_T5	3R	26531489	G-T	0.15	*plum*	FBgn0039431
lgl_T5	3R	17488169	G-T	0.14	*Dscam3*	FBgn0261046
aura T5	3R	13123360	C-A	0.16	*2mit*	FBgn0260793
lgl_T5	3R	6376459	C-A	0.15	*CG42564*	FBgn0260766
aura T5	3R	29419114	C-A	0.20	*Ptp99A*	FBgn0004369
lgl_T5	3R	15221471	C-A	0.11	*CG12241*	FBgn0038304
lgl_T5	3R	10525863	G-T	0.21	*hth*	FBgn0001235
lgl_T5	3R	20582062	G-T	0.13	*CG16953*	FBgn0038809
mbtL1 T5	3R	23446303	C-A	0.11	*CG4374*	FBgn0039078
lgl_T5	3R	27204324	G-T	0.13		
lgl_T5	3R	30161069	G-T	0.14		
lgl_T5	3R	25385238	C-A	0.14	*Fur1*	FBgn0004509
lgl_T10	3R	31185132	G-T	0.14	*mesh*	FBgn0051004
mbtL2 T10A	3R	2839832	C-G	0.44	*CG45783*	FBgn0267430
lgl_T5	3R	14829384	G-T	0.12	*btsz*	FBgn0266756
mbtL1 T0	3R	10655146	G-T	0.13		
lgl_T5	3R	13337580	G-T	0.16		
lgl_T10	3R	10374669	G-T	0.27	*Rfx*	FBgn0020379

mbtL2 T10B	3R	25434814	C-G	0.34	*Fur1*	FBgn0004509
mbtL2 T10B	3R	25369453	G-T	0.10	*Fur1*	FBgn0004509
aura T5	3R	26661371	C-A	0.11	*beat-VII*	FBgn0250908
aura T5	3R	11695431	G-T	0.14		
lgl_T10	3R	14868191	C-A	0.10	*btsz*	FBgn0266756
lgl_T5	3R	15143538	C-A	0.11	*GlyS*	FBgn0266064
aura T5	3R	16361441	G-T	0.11	*CG14880*	FBgn0038422
aura T5	3R	28354701	C-A	0.11		
lgl_T5	3R	19043419	G-T	0.12	*CG6026*	FBgn0038676
mbtL2 T5	3R	22515436	C-A	0.13	*Octbeta1R*	FBgn0038980
mbtL2 T10B	3R	21713659	T-C	0.65	*CG31176*	FBgn0051176
aura T5	3R	28647839	C-A	0.27		
mbtL2 T5	3R	12335414	G-T	0.09		
lgl_T10	3R	5081875	G-T	0.12	*corto*	FBgn0010313
lgl_T10	3R	5135803	C-A	0.13		
lgl_T5	3R	14192748	G-T	0.12	*Dop1R1*	FBgn0011582
mbtL1 T5	3R	28739133	G-T	0.16	*AstA-R2*	FBgn0039595
lgl_T10	3R	29510265	C-A	0.14		
aura T5	3R	31455739	C-A	0.12	*Gprk2*	FBgn0261988
lgl_T5	3R	29895792	G-T	0.11	*CG31038*	FBgn0051038
lgl_T5	3R	17804652	G-T	0.13	*CG17802*	FBgn0038549
aura T5	3R	20135672	G-T	0.17		
lgl_T5	3R	30088869	G-T	0.11	*sima*	FBgn0266411
mbtL1 T5	3R	20108600	C-A	0.13		
aura T5	3R	27653051	G-T	0.18	*RpS10a*	FBgn0027494
mbtL1 T5	3R	22996325	C-A	0.11	*Nha2*	FBgn0263390
lgl_T10	3R	6348126	C-A	0.15		
lgl_T10	3R	21736333	G-T	0.12		
lgl_T10	3R	24046982	G-T	0.16	*Rox8*	FBgn0005649
lgl_T10	3R	5382540	C-A	0.12	*kkv*	FBgn0001311
mbtL2 T10A	3R	2458656	C-A	0.41	*CG45784*	FBgn0267431
mbtL2 T10B	3R	2458656	C-A	0.31	*CG45784*	FBgn0267431
lgl_T5	3R	31250737	G-T	0.13	*qless*	FBgn0051005
lgl_T5	3R	25419356	G-T	0.19	*Fur1*	FBgn0004509
bratL1 T10	3R	2504999	G-A	0.13		
lgl_T10	3R	16071820	C-A	0.13	*Mhcl*	FBgn0026059
lgl_T10	3R	15535466	G-T	0.13	*AOX1*	FBgn0267408
mbtL2 T10A	3R	2873314	T-A	0.41	*CG45783*	FBgn0267430
mbtL2 T10B	3R	2873314	T-A	0.48	*CG45783*	FBgn0267430
mbtL1 T5	3R	2906632	A-T	0.27	*CG45783*	FBgn0267430
aura T5	3R	31408366	G-T	0.14	*Gprk2*	FBgn0261988
lgl_T5	3R	4413424	G-T	0.13	*CG32944*	FBgn0052944
lgl_T5	3R	5463865	G-T	0.17	*PEK*	FBgn0037327
lgl_T10	3R	29462204	C-T	0.15	*Ptp99A*	FBgn0004369
lgl_T5	3R	20134584	G-T	0.15		
lgl_T5	3R	18987720	C-A	0.13	*gukh*	FBgn0026239
lgl_T5	3R	25313083	C-A	0.16	*CG31087*	FBgn0051087
mbtL1 T5	3R	20955103	C-A	0.15	*Atpalpha*	FBgn0002921
lgl_T5	3R	30464092	G-T	0.13	*CDase*	FBgn0039774
aura T5	3R	17831191	G-T	0.17	*tinc*	FBgn0261649
lgl_T10	3R	16096527	G-T	0.12	*Mhcl*	FBgn0026059
lgl_T10	3R	29081087	G-T	0.15	*CG11873*	FBgn0039633
lgl_T5	3R	10435407	G-T	0.10	*Cyp12e1*	FBgn0037817
lgl_T5	3R	6399099	G-T	0.13	*Dmtn*	FBgn0037443
aura T5	3R	29340150	C-A	0.11		
lgl_T10	3R	11663196	G-T	0.11	*CG42327*	FBgn0259227
mbtL2 T10A	3R	2490824	T-A	0.45	*CG45784*	FBgn0267431
mbtL2 T10B	3R	2490824	T-A	0.44	*CG45784*	FBgn0267431
mbtL2 T10A	3R	2768965	T-A	0.13	*CG45783*	FBgn0267430
aura T5	3R	10178891	G-T	0.11	*CG12814*	FBgn0037796
mbtL2 T5	3R	11259406	C-A	0.13		
lgl_T10	3R	29099664	C-A	0.10	*CG11873*	FBgn0039633
mbtL1 T10	3R	9673042	C-A	0.10	*CG9492*	FBgn0037726

aura T5	3R	26206212	G-T	0.13	*CR44320*	FBgn0265379
lgl_T5	3R	6297428	C-A	0.16		
mbtL2 T10B	3R	31713770	C-T	0.52		
mbtL2 T10A	3R	28356942	C-G	0.59		
mbtL2 T10B	3R	28356942	C-G	0.56		
lgl_T5	3R	20148966	C-A	0.14		
mbtL2 T10B	3R	2505099	T-A	0.11		
mbtL2 T10A	3R	2504930	A-G	0.14		
mbtL2 T10A	3R	1022224	G-C	0.13	*CG45784*	FBgn0267431
lgl_T10	3R	10309192	C-A	0.11	*CG6293*	FBgn0037807
bratL2 T5	3R	29532435	G-T	0.24		
mbtL1 T5	3R	908634	A-G	0.43	*CG45784*	FBgn0267431
lgl_T5	3R	10448125	G-T	0.22		
lgl_T5	3R	13349955	G-T	0.17		
mbtL2 T10A	3R	2422835	C-A	0.43	*CG45784*	FBgn0267431
mbtL2 T10B	3R	2422835	C-A	0.43	*CG45784*	FBgn0267431
lgl_T10	3R	16228326	G-T	0.23	*tara*	FBgn0040071
lgl_T5	3R	7555242	C-A	0.12		
lgl_T10	3R	29852300	C-A	0.10	*Cad99C*	FBgn0039709
mbtL2 T10A	3R	2468666	A-T	0.47	*CG45784*	FBgn0267431
mbtL2 T10B	3R	2468666	A-T	0.51	*CG45784*	FBgn0267431
mbtL2 T5	3R	21495333	C-A	0.09		
lgl_T5	3R	9580548	G-T	0.13	*CG8273*	FBgn0037716
aura T5	3R	23812453	G-T	0.09	*CG34355*	FBgn0085384
lgl_T10	3R	26480349	G-T	0.09	*plum*	FBgn0039431
aura T5	3R	31061448	G-T	0.21		
lgl_T10	3R	5480823	G-T	0.10	*ksr*	FBgn0015402
lgl_T5	3R	31926621	G-T	0.13	*heph*	FBgn0011224
lgl_T10	3R	18384783	C-A	0.10	*CG14309*	FBgn0038611
mbtL2 T10A	3R	2747208	T-A	0.50	*CG45783*	FBgn0267430
mbtL2 T10B	3R	2747208	T-A	0.68	*CG45783*	FBgn0267430
lgl_T5	3R	14761705	C-A	0.14	*jvl*	FBgn0263929
lgl_T5	3R	5434638	G-T	0.20	*CG14669*	FBgn0037326
lgl_T10	3R	7523419	G-T	0.11	*alpha-Est7*	FBgn0015575
lgl_T10	3R	5086038	G-T	0.12	*corto*	FBgn0010313
aura T5	3R	9921927	G-T	0.10	*Glut4EF*	FBgn0267336
lgl_T5	3R	11836011	C-A	0.13	*CG6959*	FBgn0037956
lgl_T5	3R	25422900	G-T	0.10	*Fur1*	FBgn0004509
mbtL2 T10A	3R	2876302	T-G	0.49	*CG45783*	FBgn0267430
mbtL2 T10B	3R	2876302	T-G	0.47	*CG45783*	FBgn0267430
lgl_T10	3R	27474624	G-T	0.12		
lgl_T10	3R	5384184	G-T	0.13	*kkv*	FBgn0001311
lgl_T10	3R	7291902	C-A	0.13	*rn*	FBgn0267337
lgl_T5	3R	14143874	G-T	0.20		
bratL1 T10	3R	2505760	G-A	0.19		
mbtL2 T10B	3R	2505811	A-T	0.20		
lgl_T5	3R	5776771	G-T	0.10	*CG2082*	FBgn0027608
bratL1 T0	3R	2506308	A-G	0.13		
lgl_T10	3R	29057647	C-A	0.15	*Sirt7*	FBgn0039631
mbtL2 T10A	3R	2505099	T-A	0.11		
bratL2 T5	3R	3906836	G-A	0.12		
mbtL2 T0	3R	13835406	T-A	0.15	*Nsf2*	FBgn0266464
aura T5	3R	3617174	C-T	0.16		
lgl_T0	3R	3617174	C-T	0.12		
lgl_T10	3R	3617174	C-T	0.16		
lgl_T5	3R	3617174	C-T	0.11		
lgl_T0	3R	25125618	A-G	0.31	*vig2*	FBgn0046214
lgl_T10	3R	25125618	A-G	0.20	*vig2*	FBgn0046214
lgl_T5	3R	25125618	A-G	0.16	*vig2*	FBgn0046214
bratL1 T10	3R	30073531	C-G	0.63	*sima*	FBgn0266411
aura T5	3R	3617195	G-T	0.18		
lgl_T10	3R	3617195	G-T	0.17		
lgl_T5	3R	3617195	G-T	0.11		

bratL1 T10	3R	30073549	C-G	0.59	*sima*	FBgn0266411
lgl_T5	3R	6711113	G-T	0.14	*pb*	FBgn0051481
mbtL2 T10A	3R	28356956	T-G	0.51		
mbtL2 T10B	3R	28356956	T-G	0.47		
mbtL2 T10A	3R	2839845	T-A	0.41	*CG45783*	FBgn0267430
mbtL2 T10B	3R	2839845	T-A	0.39	*CG45783*	FBgn0267430
mbtL1 T0	3R	25127443	A-T	0.21		
mbtL2 T0	3R	11241983	T-C	0.08	*scpr-A*	FBgn0037889
mbtL2 T10B	3R	2505820	G-C	0.22		
mbtL2 T10B	3R	1867095	C-G	0.17	*CG45784*	FBgn0267431
mbtL2 T10A	3R	1867099	A-C	0.13	*CG45784*	FBgn0267431
mbtL2 T10A	3R	25434818	T-G	0.21	*Fur1*	FBgn0004509
mbtL2 T10A	3R	28342284	C-G	0.42	*Moca-cyp*	FBgn0039581
mbtL2 T10B	3R	1867099	A-C	0.17	*CG45784*	FBgn0267431
mbtL2 T10B	3R	25434818	T-G	0.33	*Fur1*	FBgn0004509
mbtL2 T10B	3R	28342284	C-G	0.47	*Moca-cyp*	FBgn0039581
lgl_T0	3R	25125621	A-C	0.29		
lgl_T10	3R	25125621	A-C	0.19		
lgl_T5	3R	25125621	A-C	0.16		
mbtL2 T0	3R	25125523	G-A	0.33	*vig2*	FBgn0046214
mbtL2 T10A	3R	2505102	T-A	0.11		
mbtL2 T10A	3R	25125523	G-A	0.17	*vig2*	FBgn0046214
mbtL2 T10B	3R	2505102	T-A	0.11		
mbtL2 T10B	3R	25125523	G-A	0.18	*vig2*	FBgn0046214
mbtL2 T10A	3R	18734748	C-G	0.50	*Mekk1*	FBgn0024329
mbtL2 T10B	3R	18734748	C-G	0.29	*Mekk1*	FBgn0024329
bratL1 T10	3R	31945351	A-T	0.65	*heph*	FBgn0011224
bratL1 T10	3R	31945352	A-T	0.65	*heph*	FBgn0011224
mbtL2 T10A	3R	2157580	G-C	0.57	*CG45784*	FBgn0267431
mbtL2 T10A	3R	18734749	C-A	0.50	*Mekk1*	FBgn0024329
mbtL2 T10B	3R	2157580	G-C	0.38	*CG45784*	FBgn0267431
mbtL2 T10B	3R	18734749	C-A	0.27	*Mekk1*	FBgn0024329
bratL2 T5	3R	2792823	C-T	0.27	*CG45783*	FBgn0267430
bratL1 T5	3R	2504999	G-A	0.14		
bratL1 T10	3R	2270777	C-T	0.06	*CG45784*	FBgn0267431
mbtL1 T0	3R	2513397	A-G	0.14		
aura T5	3R	3298215	T-C	0.16		
bratL2 T0	3R	3617057	T-G	0.15		
mbtL2 T5	3R	1913113	C-A	0.13	*CG45784*	FBgn0267431
aura T0	3R	2687617	C-A	0.11	*CG45783*	FBgn0267430
lgl_T0	3R	805965	G-C	0.11		
lgl_T10	3R	805965	G-C	0.13		
mbtL2 T10A	3R	1008406	A-T	0.15	*CG45784*	FBgn0267431
mbtL1 T5	3R	895330	C-A	0.15	*CG45784*	FBgn0267431
bratL1 T0	3R	399962	A-G	0.18		
mbtL2 T10B	3R	1135010	A-T	0.49	*CG45784*	FBgn0267431
lgl_T5	3R	805965	G-C	0.12		
mbtL2 T0	3R	1055973	T-G	0.17	*CG45784*	FBgn0267431
mbtL1 T10	3R	908634	A-G	0.48	*CG45784*	FBgn0267431
mbtL1 T0	X	22930475	C-G	0.32		
mbtL1 T10	X	15340174	C-A	0.19	*HDAC6*	FBgn0026428
mbtL2 T0	X	22084330	C-G	0.19		
bratL1 T0	X	13794659	T-A	0.27	*inaE*	FBgn0261244
mbtL1 T5	X	22930475	C-G	0.17		
lgl_T0	X	20006321	G-T	0.11	*Dop2R*	FBgn0053517
mbtL2 T0	X	12111089	T-A	0.30	*CR43960*	FBgn0264675
bratL1 T5	X	21515910	T-C	0.36	*CR45082*	FBgn0266457
bratL1 T10	X	12127891	G-C	0.43	*Ten-a*	FBgn0267001
aura T0	X	23327342	T-A	0.12		
bratL2 T0	X	23431925	A-G	0.18		
bratL1 T5	X	10503505	G-T	0.27	*spri*	FBgn0085443
lgl_T0	X	11665873	C-A	0.18	*Ptp10D*	FBgn0004370
mbtL2 T10A	X	18438237	C-T	1.00	*CG32548*	FBgn0052548

aura T0	X	15984401	G-T	0.12	*CG8931*	FBgn0030717
bratL2 T5	X	23431925	A-G	0.14		
bratL2 T5	X	11383631	G-T	0.21	*dlg1*	FBgn0001624
mbtL2 T10A	X	12196454	C-A	0.27	*Ten-a*	FBgn0267001
bratL1 T0	X	19042388	G-A	0.22		
mbtL2 T10B	X	10061200	C-A	0.23	*CG15312*	FBgn0030174
mbtL1 T0	X	5282350	G-T	0.13		
aura T0	X	9576114	T-A	0.43	*CG42395*	FBgn0259741
bratL1 T0	X	4954109	C-A	0.24	*Ptp4E*	FBgn0004368
mbtL2 T5	X	18438237	C-T	0.15	*CG32548*	FBgn0052548
mbtL1 T5	X	5448859	C-A	0.22	*SPR*	FBgn0029768
mbtL1 T10	X	18953903	G-T	0.24	*CG43759*	FBgn0264090
aura T0	X	5017160	C-A	0.31	*CR44833*	FBgn0266096
mbtL2 T5	X	11968405	G-T	0.17	*cac*	FBgn0263111
bratL1 T5	X	13794644	C-T	0.18	*inaE*	FBgn0261244
mbtL2 T5	X	23383959	A-T	0.19		
lgl_T5	X	8260199	A-G	0.19	*CG1632*	FBgn0030027
mbtL2 T10A	X	21347023	C-G	1.00	*pen*	FBgn0015527
bratL1 T0	X	21762610	G-A	0.63	*flam*	FBgn0267704
mbtL2 T0	X	3796056	A-G	0.22	*Crg-1*	FBgn0021738
bratL2 T5	X	16363595	G-C	0.27	*nonA*	FBgn0004227
bratL2 T0	X	16361770	C-A	0.32	*nonA*	FBgn0004227
mbtL1 T5	X	12737818	C-A	0.21	*Smr*	FBgn0265523
mbtL2 T10B	X	18438237	C-T	1.00	*CG32548*	FBgn0052548
bratL1 T10	X	4259915	T-C	0.80		
lgl_T0	X	22414059	C-A	0.14		
lgl_T5	X	16116675	C-T	0.50		
lgl_T5	X	23352963	A-C	0.07		
mbtL2 T5	X	14300971	G-T	0.17		
lgl_T10	X	6595271	A-C	0.24	*Pat1*	FBgn0029878
lgl_T10	X	21110838	C-A	0.17	*CG11566*	FBgn0031159
lgl_T0	X	5509042	G-T	0.22		
mbtL2 T5	X	3562029	G-T	0.20	*CG32791*	FBgn0052791
mbtL2 T10B	X	15952001	G-T	0.16	*mmd*	FBgn0259110
mbtL2 T10B	X	21347023	C-G	0.97	*pen*	FBgn0015527
aura T5	X	4515552	C-A	0.13	*CR32773*	FBgn0052773
bratL2 T5	X	13404581	G-T	0.21		
lgl_T10	X	9554853	G-T	0.31	*RpS28b*	FBgn0030136
mbtL1 T5	X	10026600	G-T	0.25	*Cht6*	FBgn0263132
bratL1 T10	X	14000150	A-C	0.15		
bratL1 T5	X	23327342	T-A	0.08		
lgl_T10	X	22782236	G-T	0.17	*CR44997*	FBgn0266348
lgl_T5	X	13215300	C-A	0.16	*mew*	FBgn0004456
lgl_T5	X	21005682	G-A	0.20		
aura T5	X	11640772	G-T	0.14	*Ptp10D*	FBgn0004370
lgl_T10	X	18818439	C-G	0.93	*CG42450*	FBgn0259927
mbtL1 T5	X	14278512	G-C	0.23	*Muc12Ea*	FBgn0052602
mbtL2 T5	X	7139459	G-T	0.14	*CG9650*	FBgn0029939
mbtL2 T10B	X	13762922	C-A	0.18	*rdgB*	FBgn0003218
mbtL2 T10B	X	12292325	C-G	1.00	*Ten-a*	FBgn0267001
bratL2 T5	X	17781276	G-A	0.31		
lgl_T5	X	9785971	C-A	0.17	*CG32698*	FBgn0052698
lgl_T10	X	4284242	G-T	0.19		
mbtL2 T5	X	8389513	G-T	0.13		
aura T5	X	9118957	G-T	0.16		
lgl_T10	X	12252474	G-C	0.19	*Ten-a*	FBgn0267001
aura T5	X	7893191	C-A	0.11	*CG18624*	FBgn0029971
lgl_T5	X	10887074	G-T	0.19	*Ork1*	FBgn0017561
mbtL2 T5	X	19513313	G-T	0.24	*CG14200*	FBgn0031023
mbtL2 T5	X	4635495	T-G	0.19	*CG12179*	FBgn0025388
lgl_T5	X	3972854	C-A	0.33		
aura T5	X	19487345	G-T	0.12	*pcm*	FBgn0020261
bratL1 T10	X	15323089	C-A	0.67	*Top1*	FBgn0004924

mbtL2 T5	X	5666021	C-A	0.18		
mbtL2 T10B	X	23383888	A-G	0.15		
mbtL2 T10A	X	23368634	A-G	0.10		
mbtL2 T0	X	23368602	G-A	0.08		
lgl_T0	X	23352963	A-C	0.10		
lgl_T5	X	18135891	G-T	0.24	*ari-1*	FBgn0017418
mbtL2 T10B	X	10869187	G-T	0.22		
lgl_T10	X	7607150	C-A	0.25		
lgl_T10	X	17232717	G-A	0.13		
aura T5	X	22819046	C-A	0.17		
lgl_T10	X	15689610	T-C	0.38	*CR43132*	FBgn0262606
mbtL1 T5	X	7457236	C-A	0.20		
mbtL1 T5	X	6205816	G-A	0.18		
lgl_T10	X	16440694	G-T	0.16	*CG9947*	FBgn0030752
lgl_T10	X	13994871	C-A	0.15		
aura T5	X	21396377	G-A	0.20		
mbtL2 T10A	X	22084330	C-G	0.17		
mbtL2 T10B	X	19164853	G-T	0.20	*RhoGAP18B*	FBgn0261461
lgl_T10	X	1321389	G-T	0.21	*DAAM*	FBgn0025641
lgl_T5	X	1726479	G-T	0.13		
aura T5	X	6256101	G-T	0.11	*Spx*	FBgn0015818
lgl_T10	X	10238853	A-G	0.24	*Hk*	FBgn0263220
lgl_T5	X	11568387	G-T	0.15	*RpII215*	FBgn0003277
aura T5	X	20652969	C-A	0.12		
lgl_T5	X	2914876	A-C	1.00	*kirre*	FBgn0028369
aura T5	X	22041024	T-G	0.10		
lgl_T5	X	18771361	G-T	0.19	*CCKLR-17D3*	FBgn0030954
aura T5	X	12882496	G-T	0.15	*Pde9*	FBgn0259171
lgl_T10	X	10855514	G-A	0.20	*sofe*	FBgn0030242
lgl_T5	X	4588619	G-T	0.20	*CR32773*	FBgn0052773
aura T0	X	10191130	C-A	0.16	*CR44894*	FBgn0266199
lgl_T5	X	16730153	C-A	0.20	*CG9634*	FBgn0027528
lgl_T10	X	13247742	C-A	0.21	*mew*	FBgn0004456
lgl_T5	X	19375510	C-A	0.17	*kek5*	FBgn0031016
mbtL2 T5	X	20117074	C-A	0.14		
aura T5	X	15771832	C-A	0.10	*CG33172*	FBgn0053172
mbtL2 T10B	X	22084330	C-G	0.16		
lgl_T10	X	23352957	G-T	0.07		
aura T5	X	18260016	C-A	0.10		
lgl_T5	X	13766898	G-T	0.13	*rdgB*	FBgn0003218
aura T5	X	13844742	C-A	0.09	*AMPdeam*	FBgn0052626
lgl_T5	X	5082818	C-A	0.27	*CG32767*	FBgn0052767
lgl_T5	X	17215979	G-T	0.20	*CG5162*	FBgn0030828
lgl_T10	X	14691100	G-T	0.18	*NetB*	FBgn0015774
aura T5	X	10042102	C-A	0.10	*Cht6*	FBgn0263132
aura T5	X	14298819	G-T	0.11		
mbtL1 T5	X	6657490	G-T	0.15	*l(1)G0148*	FBgn0028360
aura T5	X	12085603	C-A	0.15	*CR43960*	FBgn0264675
lgl_T10	X	2895442	C-A	0.18	*kirre*	FBgn0028369
aura T5	X	13339738	C-A	0.12	*CG15747*	FBgn0030474
lgl_T10	X	1867815	C-A	0.19	*Pex5*	FBgn0023516
mbtL1 T0	X	23352699	T-C	0.10		
aura T5	X	5210738	C-A	0.15	*CG15465*	FBgn0029746
aura T5	X	15026340	G-T	0.13	*be*	FBgn0052594
mbtL1 T5	X	23346866	C-A	0.05		
bratL2 T5	X	13794659	T-A	0.27	*inaE*	FBgn0261244
bratL1 T10	X	4649211	C-T	0.16	*Torsin*	FBgn0025615
aura T5	X	9506809	G-T	0.22	*CG32700*	FBgn0267253
mbtL1 T5	X	8122004	C-A	0.25	*cyr*	FBgn0030001
lgl_T10	X	12637203	C-A	0.17	*Tomosyn*	FBgn0030412
lgl_T5	X	2097434	G-T	0.19	*csw*	FBgn0000382
aura T5	X	16211062	C-A	0.42	*disco*	FBgn0000459
aura T5	X	19976237	C-A	0.12		

aura T5	X	16790686	C-A	0.16	*Rrp45*	FBgn0030789
aura T5	X	5564729	T-C	0.43		
lgl_T10	X	2216876	G-T	0.25	*Vinc*	FBgn0004397
mbtL2 T0	X	22425859	A-T	0.21		
mbtL2 T10A	X	22425859	A-T	0.14		
aura T5	X	23327319	T-G	0.08		
lgl_T5	X	1003121	G-T	0.14		
mbtL2 T10B	X	22386795	T-A	0.11		
aura T5	X	17699328	G-T	0.14	*CG8188*	FBgn0030863
aura T5	X	17081479	C-A	0.16	*CG4955*	FBgn0030814
aura T5	X	14613359	G-T	0.16	*NetA*	FBgn0015773
bratL1 T10	X	14279565	G-A	0.20	*Muc12Ea*	FBgn0052602
mbtL1 T5	X	7735163	G-T	0.16	*Rab39*	FBgn0029959
aura T5	X	17357172	C-A	0.13		
aura T5	X	4785502	C-A	0.11	*CG6978*	FBgn0029727
aura T5	X	1865233	G-T	0.17	*CG14814*	FBgn0023515
lgl_T10	X	596096	G-T	0.19	*vnd*	FBgn0261930
lgl_T10	X	2450671	C-A	0.15	*boi*	FBgn0040388
aura T5	X	1051621	C-A	0.13	*CG14629*	FBgn0040398
lgl_T10	X	14913061	G-T	0.16	*Flo2*	FBgn0264078
aura T5	X	16431342	C-A	0.13	*hang*	FBgn0026575
mbtL2 T5	X	8605505	G-T	0.17	*Caf1-180*	FBgn0030054
mbtL2 T5	X	1165200	C-T	0.30		
lgl_T10	X	14188254	G-A	0.15		
aura T5	X	1441601	C-A	0.13	*futsch*	FBgn0259108
lgl_T5	X	5263034	G-T	0.21		
aura T5	X	22995904	G-T	0.17	*stnA*	FBgn0016976
lgl_T10	X	11022196	C-A	0.27	*CG42339*	FBgn0259241
aura T5	X	2394670	C-A	0.17		
aura T5	X	2230520	C-A	0.12	*pcx*	FBgn0003048
aura T5	X	15183744	C-A	0.12	*CG43737*	FBgn0263994
mbtL1 T5	X	10182834	C-A	0.20	*CR44894*	FBgn0266199
aura T5	X	6673438	G-T	0.14	*CG3198*	FBgn0029887
mbtL2 T5	X	1319170	G-T	0.14	*DAAM*	FBgn0025641
mbtL2 T10B	X	21499949	G-T	0.18	*CR45082*	FBgn0266457
aura T5	X	18412749	C-A	0.12		
aura T5	X	12233485	G-T	0.29	*Ten-a*	FBgn0267001
aura T5	X	1604118	G-T	0.15	*Mur2B*	FBgn0025390
aura T5	X	1249821	C-A	0.13	*Atf3*	FBgn0028550
aura T5	X	19614219	C-A	0.08	*Tyler*	FBgn0031038
lgl_T5	X	8381677	C-A	0.19		
lgl_T10	X	6713761	G-A	0.17	*CG14441*	FBgn0029895
aura T5	X	6519468	C-A	0.14	*CG42340*	FBgn0259242
bratL1 T10	X	4756264	G-C	0.44		
aura T5	X	6774297	C-A	0.13	*shf*	FBgn0003390
lgl_T10	X	6811098	C-T	0.50	*pod1*	FBgn0029903
mbtL2 T10A	X	12292325	C-G	0.95	*Ten-a*	FBgn0267001
lgl_T10	X	1412543	G-T	0.17	*futsch*	FBgn0259108
aura T5	X	6346456	G-T	0.13		
aura T5	X	1975147	C-A	0.16	*Hr4*	FBgn0264562
lgl_T5	X	2263323	G-T	0.14	*CG3071*	FBgn0023527
lgl_T5	X	17290783	G-T	0.17	*CG5445*	FBgn0030838
aura T5	X	15843562	C-A	0.16	*Aats-arg*	FBgn0027093
aura T5	X	9574918	G-T	0.18	*CG32699*	FBgn0052699
aura T5	X	1115242	G-T	0.14	*CG3655*	FBgn0040397
lgl_T10	X	11081568	C-A	0.15	*sev*	FBgn0003366
aura T5	X	2031512	C-A	0.21	*Actn*	FBgn0000667
aura T5	X	6402517	G-T	0.11		
lgl_T5	X	2185673	G-T	0.19		
aura T5	X	17398210	G-T	0.10	*B-H1*	FBgn0011758
mbtL2 T10B	X	22425859	A-T	0.20		
mbtL1 T5	X	23383973	A-C	0.16		
lgl_T5	X	2131600	G-T	0.19	*ph-p*	FBgn0004861

lgl_T10	X	14220317	C-A	0.19	*l(1)G0007*	FBgn0026713
mbtL1 T0	X	23383973	A-C	0.21		
aura T5	X	14326889	C-A	0.11	*dpr8*	FBgn0052600
aura T5	X	1468793	G-T	0.12	*CG14778*	FBgn0029580
mbtL1 T5	X	6683606	C-A	0.18	*CG4095*	FBgn0029890
mbtL1 T5	X	22956578	A-T	0.67		
aura T5	X	23353002	C-A	0.09		
aura T5	X	18435970	C-A	0.11	*CG32548*	FBgn0052548
aura T5	X	12256480	G-T	0.21	*Ten-a*	FBgn0267001
aura T5	X	2054222	C-A	0.10	*CG4313*	FBgn0025632
aura T5	X	1885389	C-A	0.11	*trr*	FBgn0023518
lgl_T10	X	1432533	G-T	0.18	*futsch*	FBgn0259108
aura T5	X	12900484	G-T	0.14	*CG32649*	FBgn0052649
mbtL2 T0	X	23383959	A-T	0.21		
mbtL2 T10A	X	23383941	C-T	0.09		
aura T5	X	2068543	G-T	0.13	*CG4199*	FBgn0025628
lgl_T5	X	682587	C-A	0.20		
aura T5	X	4794336	G-T	0.14		
aura T5	X	1888367	G-T	0.08	*trr*	FBgn0023518
aura T5	X	14329134	C-A	0.14	*dpr8*	FBgn0052600
aura T5	X	6404694	C-A	0.12		
bratL2 T0	X	16363595	G-C	0.16	*nonA*	FBgn0004227
bratL2 T0	X	16364008	T-C	0.11	*nonA*	FBgn0004227
mbtL2 T10B	X	23383959	A-T	0.24		
mbtL2 T10B	X	22425923	A-G	0.16		
mbtL2 T0	X	23368634	A-G	0.08		
aura T5	X	23327342	T-A	0.12		
bratL1 T0	X	13794685	T-A	0.24	*inaE*	FBgn0261244
bratL1 T5	X	13794685	T-A	0.18	*inaE*	FBgn0261244
bratL2 T0	X	13794685	T-A	0.23	*inaE*	FBgn0261244
bratL1 T5	X	13794659	T-A	0.19	*inaE*	FBgn0261244
bratL2 T0	X	13794659	T-A	0.22	*inaE*	FBgn0261244
bratL2 T0	X	16361784	T-A	0.35	*nonA*	FBgn0004227
bratL1 T10	X	15323101	C-G	0.68	*Top1*	FBgn0004924
bratL1 T0	X	13794669	C-T	0.26	*inaE*	FBgn0261244
bratL1 T5	X	13794669	C-T	0.17	*inaE*	FBgn0261244
bratL2 T0	X	13794669	C-T	0.24	*inaE*	FBgn0261244
aura T0	X	23327349	T-C	0.12		
aura T5	X	23327349	T-C	0.12		
lgl_T10	X	23352963	A-C	0.12		
lgl_T10	X	14188256	A-G	0.14		
lgl_T10	X	12252475	T-C	0.21	*Ten-a*	FBgn0267001
bratL2 T0	X	13794644	C-T	0.23	*inaE*	FBgn0261244
bratL1 T0	X	668712	C-A	0.22		
mbtL2 T0	X	1165279	C-G	0.47		
mbtL2 T10A	X	6732437	A-T	0.17	*CG3168*	FBgn0029896
mbtL1 T0	X	539025	G-T	0.19	*Appl*	FBgn0000108
aura T0	X	1502118	G-T	0.11	*Nmdar2*	FBgn0053513
bratL2 T5	X	5848225	G-A	0.33		
mbtL2 T10B	X	5269278	C-A	0.20		
bratL1 T5	X	4259915	T-C	0.33		
lgl_T0	X	3250473	C-A	0.22	*Sgs4*	FBgn0003374
mbtL1 T10	X	2909065	G-T	0.19	*kirre*	FBgn0028369
mbtL1 T5	X	1702997	C-A	0.17		
bratL1 T10	X	1790571	T-G	0.21	*CG42666*	FBgn0261548
mbtL2 T5	X	957483	G-T	0.18		
aura T5	X	827655	G-T	0.50	*CG43867*	FBgn0264449
lgl_T5	X	673545	G-T	0.20		
lgl_T10	X	361346	G-T	0.13	*CG32816*	FBgn0052816
mbtL1 T10	Y	3167809	C-A	0.17		
mbtL1 T5	Y	3163333	C-T	0.13		
lgl_T10	Y	3163348	A-T	0.08		
mbtL1 T0	Y	3163333	C-T	0.12		

mbtL2 T0	Y	3163018	C-T	0.18		
mbtL2 T10B	Y	2515649	G-T	0.11		
mbtL2 T10A	Y	2515649	G-T	0.14		
lgl_T5	Y	2512959	C-T	0.07		
lgl_T0	Y	2512959	C-T	0.08		
mbtL1 T10	Y	908053	A-G	0.12		
lgl_T0	Y	3163348	A-T	0.09		
lgl_T5	Y	3163348	A-T	0.09		
mbtL2 T10A	Y	3163018	C-T	0.19		
mbtL2 T10B	Y	3163018	C-T	0.13		
mbtL2 T5	Y	884921	C-G	0.04		
mbtL1 T0	Y	3604045	G-T	0.29	*Ory*	FBgn0046323
mbtL1 T10	Y	3603980	G-T	0.16	*Ory*	FBgn0046323
mbtL1 T10	Y	3363828	C-T	0.13		
lgl_T0	Y	959779	T-C	0.08		
lgl_T10	Y	932316	C-A	0.05		
mbtL2 T0	Y	3273370	G-C	0.09		
mbtL2 T10B	Y	3258376	G-A	0.06		
lgl_T0	Y	3250622	A-T	0.08		
lgl_T10	Y	3250622	A-T	0.10		
mbtL1 T5	Y	3250444	C-A	0.14		
lgl_T0	Y	1004303	A-G	0.32	*Su(Ste):CR45795*	FBgn0267445
mbtL2 T0	Y	932704	G-A	0.10		
mbtL2 T10B	Y	958628	T-G	0.16		
mbtL1 T10	Y	958628	T-G	0.21		
mbtL1 T0	Y	958628	T-G	0.13		
mbtL1 T5	Y	3286560	C-A	0.11		
mbtL2 T5	Y	919802	C-T	0.14		
mbtL2 T10A	Y	958628	T-G	0.21		
mbtL2 T5	Y	955703	G-A	0.10		
lgl_T10	Y	959779	T-C	0.08		
lgl_T5	Y	959779	T-C	0.12		
mbtL1 T5	Y	958628	T-G	0.13		
mbtL2 T10A	Y	919802	C-T	0.15		
mbtL2 T10B	Y	919802	C-T	0.17		
mbtL2 T0	Y	980107	A-G	0.08	*Su(Ste):CR42432*	FBgn0259863
mbtL1 T0	Y	908053	A-G	0.11		
mbtL1 T5	Y	908053	A-G	0.08		
mbtL2 T0	Y	945853	A-G	0.25		
mbtL2 T0	Y	958628	T-G	0.16		
mbtL2 T10A	Y	897274	C-G	0.12	*Su(Ste):CR41533*	FBgn0085673
mbtL1 T10	Y	919810	C-A	0.11		
mbtL1 T5	Y	919810	C-A	0.11		
mbtL1 T0	Y	919810	C-A	0.18		
mbtL1 T5	Y	893873	A-G	0.04	*Su(Ste):CR42407*	FBgn0259838
mbtL1 T5	Y	931331	G-C	0.09		
lgl_T5	Y	925481	A-T	0.07		
lgl_T5	Y	932316	C-A	0.06		
mbtL2 T10A	Y	925478	T-G	0.06		
mbtL2 T5	Y	925478	T-G	0.09		
mbtL2 T5	Y	959738	G-A	0.09		
mbtL1 T5	Y	882652	C-A	0.15		
mbtL1 T0	Y	960395	C-G	0.14		
mbtL1 T10	Y	960395	C-G	0.11		
mbtL1 T0	Y	921299	A-C	0.15		
mbtL1 T5	Y	921299	A-C	0.21		
mbtL1 T10	Y	921198	C-T	0.05		
mbtL2 T0	Y	959738	G-A	0.06		
mbtL2 T10A	Y	959738	G-A	0.09		
mbtL2 T10B	Y	959738	G-A	0.10		
mbtL2 T0	Y	960471	G-T	0.07		
mbtL2 T10A	Y	960395	C-G	0.09		
mbtL2 T10B	Y	960395	C-G	0.16		

lgl_T5	Y	960395	C-G	0.10		
mbtL1 T0	Y	908159	T-C	0.16		
mbtL2 T10A	Y	960471	G-T	0.09		
mbtL2 T0	Y	980124	G-C	0.07	*Su(Ste):CR42432*	FBgn0259863
mbtL2 T10A	Y	2515665	A-T	0.11		
mbtL2 T10B	Y	2515665	A-T	0.10		
mbtL2 T0	Y	3273377	G-A	0.09		
mbtL2 T10A	Y	2515671	G-T	0.11		
mbtL2 T10B	Y	2515671	G-T	0.10		
mbtL1 T5	Y	3286565	T-G	0.10		
mbtL2 T10A	Y	2515675	G-T	0.11		
mbtL2 T10B	Y	2515675	G-T	0.10		
mbtL2 T10A	Y	925481	A-T	0.08		
mbtL2 T5	Y	925481	A-T	0.11		
mbtL1 T10	Y	19244	C-A	0.21		
lgl_T5	Y	917170	G-T	0.04	*Su(Ste):CR42430*	FBgn0259861
mbtL2 T10B	Y	897274	C-G	0.21	*Su(Ste):CR41533*	FBgn0085673
mbtL1 T0	Y	893873	A-G	0.06	*Su(Ste):CR42407*	FBgn0259838
mbtL2 T0	Y	893842	G-A	0.04	*Su(Ste):CR42407*	FBgn0259838
mbtL2 T10A	Y	884874	T-G	0.06		
mbtL1 T5	Y	879253	C-A	0.10		
lgl_T0	Y	782496	A-C	0.16		
lgl_T10	Y	782450	G-T	0.24		
mbtL2 T5	Y	245125	C-A	0.12		

Table S3.
Catalogue of SNPs found in the cohort.

chromosome arm	start	end	width	sample
3R	2741476	3144138	402663	mbtL2_T10A
3R	2741476	3144138	402663	mbtL2_T10B
3R	2355404	2629611	274208	mbtL2_T10A
3R	2355404	2629611	274208	mbtL2_T10B
X	23322332	23483920	161589	mbtL2_T10B
3R	123488	262326	138839	mbtL2_T5
X	23322332	23457596	135265	mbtL2_T10A
3R	172978	298106	125129	bratL1 T0
3R	11465	117484	106020	bratL1 T0
3R	1794677	1895230	100554	mbtL2_T10B
3R	2109034	2207579	98546	mbtL2_T10A
3R	2109034	2207579	98546	mbtL2_T10B
2R	4967109	5064541	97433	mbtL1_T0
3R	2454645	2550805	96161	bratL1 T0
3R	2454645	2550564	95920	bratL1 T0
3R	3573060	3666406	93347	LGL T0
3R	3572568	3663606	91039	mbtL2_T0
3R	459727	544718	84992	bratL1 T0
3R	140180	224211	84032	bratL1 T0
3R	2452	86359	83908	mbtL2_T5
3R	1794677	1877857	83181	mbtL2_T10A
2R	6266463	6348983	82521	mbtL1_T10
3R	2791491	2873084	81594	bratL1 T0
X	22400384	22481366	80983	mbtL1_T10
3R	2236	77590	75355	bratL2_T5
3R	2479	77525	75047	bratL1 T0
3R	3148162	3221445	73284	mbtL2_T10B
3R	3148162	3216528	68367	mbtL2_T10A
4	1278464	1346828	68365	mbtL1_T0
3R	11562	78800	67239	bratL2_T0
3L	8712762	8776689	63928	bratL2_T0
2R	6287512	6349543	62032	mbtL2_T10B
3R	3603972	3663601	59630	mbtL2_T5
3R	3891	60726	56836	LGL T10
3L	27077496	27132033	54538	mbtL2_T0
2L	23086252	23139855	53604	mbtL2_T0
2R	6951	58387	51437	aura T0
2R	5730	56976	51247	aura T5
3R	1611863	1662549	50687	mbtL2_T10B
2L	23089369	23139999	50631	bratL1 T0
3R	3517458	3568081	50624	mbtL2_T10B
3R	2246192	2296450	50259	mbtL2_T10A
3R	2246192	2296450	50259	mbtL2_T10B
2R	4708707	4758762	50056	bratL2_T5
3R	3603972	3654008	50037	mbtL2_T10B
2R	4288047	4338082	50036	mbtL2_T0
2R	6279734	6329763	50030	mbtL2_T10A
3L	25496611	25546639	50029	mbtL2_T10A
2R	519386	569407	50022	mbtL2_T10A
2R	6279734	6329753	50020	mbtL1_T5
3R	3874569	3924587	50019	bratL2_T5
2R	2906777	2956794	50018	mbtL2_T0
3R	3603972	3653986	50015	mbtL2_T10A
3R	1611863	1661874	50012	mbtL2_T10A
3R	2496922	2546932	50011	bratL1 T5
2L	22625598	22675606	50009	bratL2_T0
X	22407550	22457553	50004	LGL T0
3R	1196858	1246860	50003	mbtL2_T10A
X	96854	146854	50001	bratL1 T0
3L	13782336	13832336	50001	bratL1 T0

3L	27076924	27126924	50001	bratL1 T0
2L	22514717	22564717	50001	bratL1 T0
3L	24633459	24683459	50001	bratL1 T5
3L	26867045	26917045	50001	bratL1 T5
2R	5099911	5149911	50001	bratL1 T5
2R	6269919	6319919	50001	bratL1 T5
X	13794644	13844644	50001	bratL2_T0
X	16361770	16411770	50001	bratL2_T0
3R	1612521	1662521	50001	bratL2_T0
3R	3017214	3067214	50001	bratL2_T0
3L	25016844	25066844	50001	bratL2_T0
3R	140452	190452	50001	bratL2_T5
3R	3022186	3072186	50001	bratL2_T5
3L	25503394	25553394	50001	LGL T0
3R	3573060	3623060	50001	LGL T10
3L	23058129	23108129	50001	mbtL1_T0
3L	23789199	23839199	50001	mbtL1_T0
3L	26477658	26527658	50001	mbtL1_T0
3L	27076727	27126727	50001	mbtL1_T0
2R	4207559	4257559	50001	mbtL1_T0
2R	6279753	6329753	50001	mbtL1_T0
3L	23789199	23839199	50001	mbtL1_T10
3L	27172800	27222800	50001	mbtL1_T10
4	284138	334138	50001	mbtL1_T10
3L	23058129	23108129	50001	mbtL1_T5
4	283833	333833	50001	mbtL1_T5
X	23345580	23395580	50001	mbtL2_T0
3R	2452	52452	50001	mbtL2_T0
3R	2261094	2311094	50001	mbtL2_T0
3L	23383282	23433282	50001	mbtL2_T0
3R	3518081	3568081	50001	mbtL2_T10A
X	22386795	22436795	50001	mbtL2_T10B
3R	214282	264282	50001	mbtL2_T10B
3L	24513867	24563867	50001	mbtL2_T10B
2R	2906777	2956777	50001	mbtL2_T10B
3R	264294	314294	50001	mbtL2_T5

Table S4.
SNP cluster analyses.

**SNPeff**	**mbtL1 T0**	**mbtL1 T5**	**mbtL1 T10**	**mbtL2 T0**	**mbtL2 T5**	**mbtL2 T10a**	**mbtL2 T10b**	**bratL1 T0**	**bratL1 T5**	**bratL1 T10**	**bratL2 T0**	**bratL2 T5**	**aura T0**	**aura T5**	**lgl T0**	**lgl T5**	**lgl T10**	
DOWNSTREAM	10	61	24	11	32	17	32	7	9	11	16	8	9	103	14	121	112	
INTERGENIC	12	28	10	21	18	18	22	10	6	7	6	5	5	40	11	45	37	
INTRON	20	64	32	19	38	56	60	9	9	29	5	4	14	93	13	143	107	
NON_SYNONYMOUS_CODING	4	36	9	10	27	21	19	1	2	6	4	4	8	75	2	96	89	EXOME
SPLICE_SITE_ACCEPTOR	-	1	-	-	-	-	-	-	-	-	-	-	-	-	-	-	3	
START_GAINED	-	1	1	-	2	-	-	-	-	-	-	-	-	4	-	4	1	
STOP_GAINED	-	1	1	-	-	-	-	-	-	-	-	-	1	-	-	2	1	
STOP_LOST	1	-	-	-	-	-	-	-	-	-	-	-	-	-	-	-	-	
SYNONYMOUS_CODING	4	16	9	10	10	4	2	-	2	3	1	2	6	48	3	34	51	
UPSTREAM	2	9	9	5	11	3	4	-	-	-	1	3	2	19	5	30	21	
UTR_3_PRIME	-	-	-	-	-	-	-	-	-	1	-	-	-	1	-	1	3	
UTR_5_PRIME	-	1	-	-	-	-	-	-	-	-	-	-	-	2	-	-	1	
SNPs in the whole genome	0.5	2.1	0.9	0.7	1.3	1.2	1.4	0.3	0.3	0.6	0.3	0.3	0.4	3.8	0.5	4.6	4.2	
SNPs in exome (all SNPs in our cohort)	0.3	1.8	0.7	0.7	1.3	0.8	0.7	0.0	0.1	0.3	0.2	0.2	0.5	4.0	0.2	4.3	5.3	
Exome (with sufficient coverage to call SNPs)	29	31	31	30	30	31	31	31	31	31	32	32	31	32	31	31	27	
SNPs freq≥0.1 (as in Lawrence et al., 2013)	0.3	1.6	0.4	0.3	0.3	0.5	0.5	0.0	0.1	0.3	0.1	0.2	0.4	3.2	0.1	3.6	3.9	

Table S5.
SNPs types found in the cohort.

	% of conserved SNPs (n/tot)	
	**T0 -> T5**	**T5 -> T10**
**mbtL1**	24.5 (13/53)	6.5 (14/218)
**mbtL2**	9 (7/76)	8 (11/138)
**bratL1**	26 (7/27)	8 (8/28)
**bratL2**	15 (5/33)	-
**aura**	11 (5/45)	-
**lgl**	25 (12/48)	3 (13/477)

Table S6.
Percentage SNPs passed on to later time points.
